# Honing in on bioluminescent milky seas from space

**DOI:** 10.1038/s41598-021-94823-z

**Published:** 2021-07-29

**Authors:** Steven D. Miller, Steven H. D. Haddock, William C. Straka, Curtis J. Seaman, Cynthia L. Combs, Menghua Wang, Wei Shi, SungHyun Nam

**Affiliations:** 1 Colorado State University, Fort Collins, CO 80523 USA; 2 Monterey Bay Aquarium Research Institute, Moss Landing, CA 95039 USA; 3 University of Wisconsin-Madison, Madison, WI 53706 USA; 4 National Oceanic and Atmospheric Administration, Center for Satellite Applications and Research, College Park, MD 20740 USA; 5 Seoul National University, Seoul, 08826 Republic of Korea

**Keywords:** Marine microbiology, Microbial ecology, Marine biology, Environmental impact

## Abstract

Milky seas are a rare form of marine bioluminescence where the nocturnal ocean surface produces a widespread, uniform and steady whitish glow. Mariners have compared their appearance to a daylit snowfield that extends to all horizons. Encountered most often in remote waters of the northwest Indian Ocean and the Maritime Continent, milky seas have eluded rigorous scientific inquiry, and thus little is known about their composition, formation mechanism, and role within the marine ecosystem. The Day/Night Band (DNB), a new-generation spaceborne low-light imager, holds potential to detect milky seas, but the capability has yet to be demonstrated. Here, we show initial examples of DNB-detected milky seas based on a multi-year (2012–2021) search. The massive bodies of glowing ocean, sometimes exceeding 100,000 km^2^ in size, persist for days to weeks, drift within doldrums amidst the prevailing sea surface currents, and align with narrow ranges of sea surface temperature and biomass in a way that suggests water mass isolation. These findings show how spaceborne assets can now help guide research vessels toward active milky seas to learn more about them.

## Introduction

Milky seas^1–4^ are understood as a kind of marine bioluminescence^5^ arising from a saprophytic relationship between luminous bacteria and microalgae that expresses on the macroscale. The bacterial colonies begin to glow when their populations attain a critical concentration (~ 10^8^ cells mL^−1^), with quorum sensing of a bacteria-secreted autoinducer triggering the bioluminescent response^1,6^. This process usually occurs on the microscopic scale, causing discrete particles to glow steadily; here, for the assumed purpose of luring a fish, whose gut offers an ideal habitat to sustain the growing bacterial colony, to consume the particle^7,8^. When the same process occurs *en masse*—scaled up to billions upon trillions of colonized particles distributed across thousands of square km of ocean surface, the visual effect is a steadily and uniformly glowing sea.

Our main awareness of milky seas draws primarily from mariner accounts^2^ with 235 sightings catalogued over the period 1915–1993 (~ 3 per year), concentrated in (and biased toward) the major shipping lanes. Additional previously unreported encounters are recounted in Supplementary Discussion 1. Although reported sporadically across the world’s ocean, milky seas are most common to the northwest Indian Ocean and the waters around Indonesia—areas characterized by deep upwelling, high primary production, and warm surface waters. A commonality among the ship reports is a widespread and steady glow in the absence of mechanical stimulus (e.g., breaking waves or ship wake). These characteristics distinguish milky seas from other, more common displays of ocean surface bioluminescence caused by dinoflagellates, which produce only a localized, transient flash of light (~ 0.1 s, approximately 10-times brighter than luminous bacteria), and whose sparkle serves as a burglar-alarm defense mechanism to startle away predators or draw the attention of larger predators^5^—an intent opposite to the postulated bacterial function of host attraction.

Despite an abundance of highly productive waters worldwide^9^, milky seas are rare. The lone research vessel encounter happened by chance in the Arabian Sea, east of Socotra, in July 1985^3^. Their water samples identified the luminous bacterium *V. harveyi* (a free-living species common to these waters^10^) colonizing upon the microalgae *Phaocystis*. This finding led to the hypothesis of milky seas forming in association with large surface slicks of organic material. In January 1995, the *S.S. Lima*^11^ traversed a milky sea ~ 90 km offshore that was later confirmed from space^4,12^ by the Defense Meteorological Satellite Program (DMSP) Operational Linescan System (OLS). Despite its poor quality, the OLS imagery revealed a ~ 15,400 km^2^ comma-shaped luminous region whose boundaries matched the *S.S. Lima*’s entry and exit coordinates. The glowing swath of ocean persisted for several consecutive nights, wrapping slowly around the eastern side of a cold-core eddy.

For lack of detailed observations, many elementary questions about milky sea structure, composition, and formation processes remain unanswered. Addressing these questions requires a practical way to identify, intercept, and measure milky seas in situ. Earth-orbiting satellites, capable of surveying the global ocean, could serve as an alert and targeting system for milky seas. However, the crude imagery quality of low-light sensors like the OLS is insufficient to identify events proactively and independent of surface confirmation.

**Figure 1:**
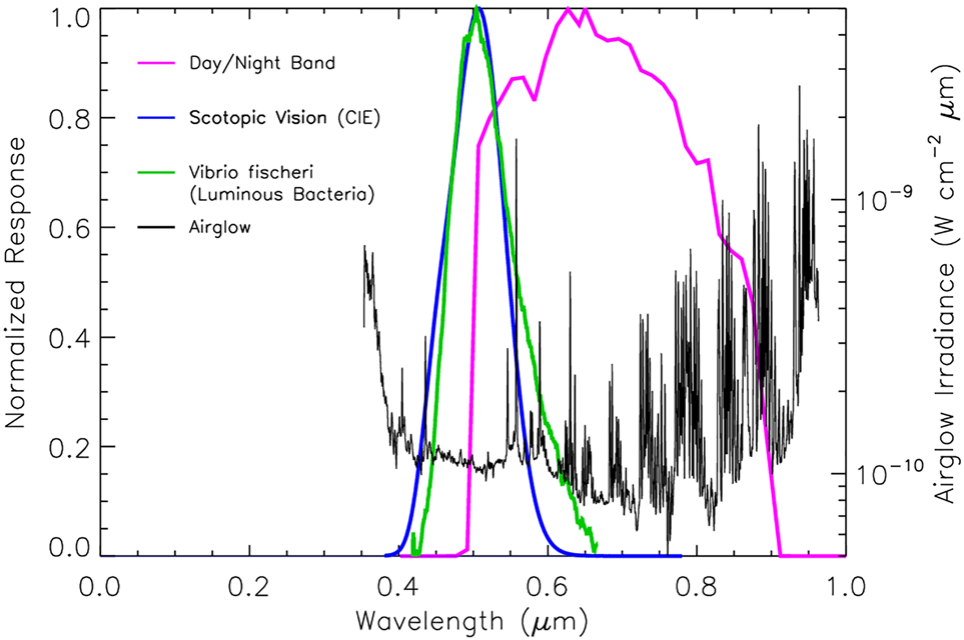
Comparing the relative response of the Day/Night Band (magenta line) and night time human vision (scotopic; blue line) to the normalized emission spectra for the luminous bacteria *Vibrio fischeri* (green line) and atmospheric airglow emissions (black line).

The Day/Night Band (DNB), part of the Visible Infrared Imaging Radiometer Suite (VIIRS^13^), offers new hope for the autonomous detection of milky seas. The DNB is a low-light visible/near-infrared (500–900 nm spectral bandpass; Fig. 1) scanning radiometer offering global coverage once per day, built-up from composites of its 3060 km-wide imagery swath. It is carried on two National Oceanic and Atmospheric Administration (NOAA) operational satellites: the *Suomi* National Polar-orbiting Partnership (*Suomi* NPP; launched in October 2011) and the Joint Polar Satellite System series (JPSS; its first member, NOAA-20, launched in November 2017). These two satellites fly in the same 834 km altitude sun-synchronous orbital plane, displaced ½ orbit (~ 50 min) from one another, providing a ~ 1330 local daytime overpass and corresponding ~ 0130 local nighttime overpass. Global DNB data are available from NOAA in near real-time—within 1–2 h of observation—or in real-time when collected via direct-readout by a terrestrial receiver that is within line-of-sight of the satellite.

The DNB offers several key advances over the OLS, including higher spatial resolution (0.742 km pixels vs. ~ 5 km for the OLS), 256-times greater detail in brightness gradation, and calibration that permits quantitative analyses^14^. Importantly, the DNB’s noise-floor (i.e., where the signal-to-noise ratio, SNR, is ~ 1) is 5 × 10^–11^ W cm^−2^ sr^−1^. This sensitivity is roughly one-hundred times greater than that of the OLS, and enables detection of light that is about ten-thousand times fainter than reflected moonlight and ~ 1 billion times fainter than reflected sunlight.

Despite these advances, the DNB has several limitations from the standpoint of milky sea detection. Moonlight, which is 2–3 orders of magnitude brighter than bioluminescent signals, dominates roughly half the nights of the ~ 29.5 day lunar cycle. Figure 1 shows that the DNB’s spectral response is also sensitive to mesospheric airglow emissions^15^, which occur as both reflected light off the clouds and as direct upwelling emissions to space^16^. Atmospheric gravity waves modulate the intensity of the latter^17^, forming patterns of brightness having spatial scales similar to those expected from milky seas. However, unlike milky seas, cloud and airglow patterns are ephemeral and do not persist over multiple nights. The DNB’s spectral response overlaps only partially with the blue/green-light bioluminescent emissions (Fig. 1), offering about 2-times less overlap to those emissions than the OLS^4^ and ~ 4 times less than the human eye’s scotopic (dark-adapted) vision response. There is decreasing sensitivity from nadir toward scan-edge of the DNB image swath, due to a physically decreasing detector size^18^ and an increasing path through the airglow^19^. These many caveats raised doubts as to whether the DNB would be able to detect milky seas at all.

## Results

We processed DNB imagery from three key regions per the historical record of mariner sightings^2^—the northwest Indian Ocean (5° S–20° N, 40–70° E) and Indonesian waters surrounding Java (15° S–0°, 100–115° E) and the Banda Sea (11–1° S, 120–135° E). Our search window spanned 2012–2021, during the periods December–March and July–September corresponding to the peak modes of ship sightings. Data processing and detection criteria are described in “Methods” section.

Our search yielded 12 DNB-detected events (listed in Table 1) whose properties met the strict criteria for milky seas. Physically unexplainable in terms of thermal emissions (which would require scene temperatures exceeding 600 K), uncorrelated with clouds/airglow, invisible during the day, and persistent over multiple consecutive nights, these luminous bodies drifted and evolved in ways that were consistent with the analyzed ocean surface currents. The start and end dates of detection were in many cases bound by the observable periods as defined by the lunar cycle. Here, we highlight three exemplary cases, with additional details for all cases summarized in Supplementary Discussion 2.

**Table 1:** Day/Night Band detected milky sea events identified in this study.

Case year and region	Center lat/lon	Start obs	End obs		Area (km^2^)
2013 Socotra	15 N/58 E	31 Jul	13 Aug		9000
2014 Banda	5S/126 E	20 Aug	24 Aug		18,000
2015 Somalia Phase 1	0/44 E	15 Jan	28 Jan		23,000
2015 Somalia Phase 2	0/50 E	21 Jan	26 Jan		60,000
2015 Banda	5S/129 E	12 Aug	18 Aug		30,000
2015 Socotra Phase 1	10 N/53 E	07 Sep	11 Sep		750
2015 Socotra Phase 2	11 N/52 E	12 Sep	20 Sep		12,000
2017 Somalia	2 N/47 E	21 Jan	31 Jan		17,000
2018 Somalia Phase 1	2 N/47 E	12 Jan	19 Jan		30,000
2018 Somalia Phase 2	5 N/55 E	19 Jan	24 Jan		15,000
2019 Somalia	2 N/50 E	28 Jan	07 Feb		100,000
2019 Java Phase 1	9 S/110 E	25 Jul	09 Aug		100,000
2019 Java Phase 2	9 S/110 E	25 Aug	07 Sep		50,000
2019 Banda	5 S/127 E	26 Jul	04 Aug		60,000
2021 Socotra/Somalia Phase 1	11 S/58 E	07 Jan	22 Jan		10,000
2021 Socotra/Somalia Phase 2	7 N/52 E	15 Jan	18 Jan		20,000
2021 Socotra	8 N/56 E	07 Feb	20 Feb		6000

Approximate centroid locations and spatial area estimates correspond to the maximum observed size of each event. Full case details are provided in Supplementary Discussion 2.

### Socotra, July/August 2013

**Figure 2:**
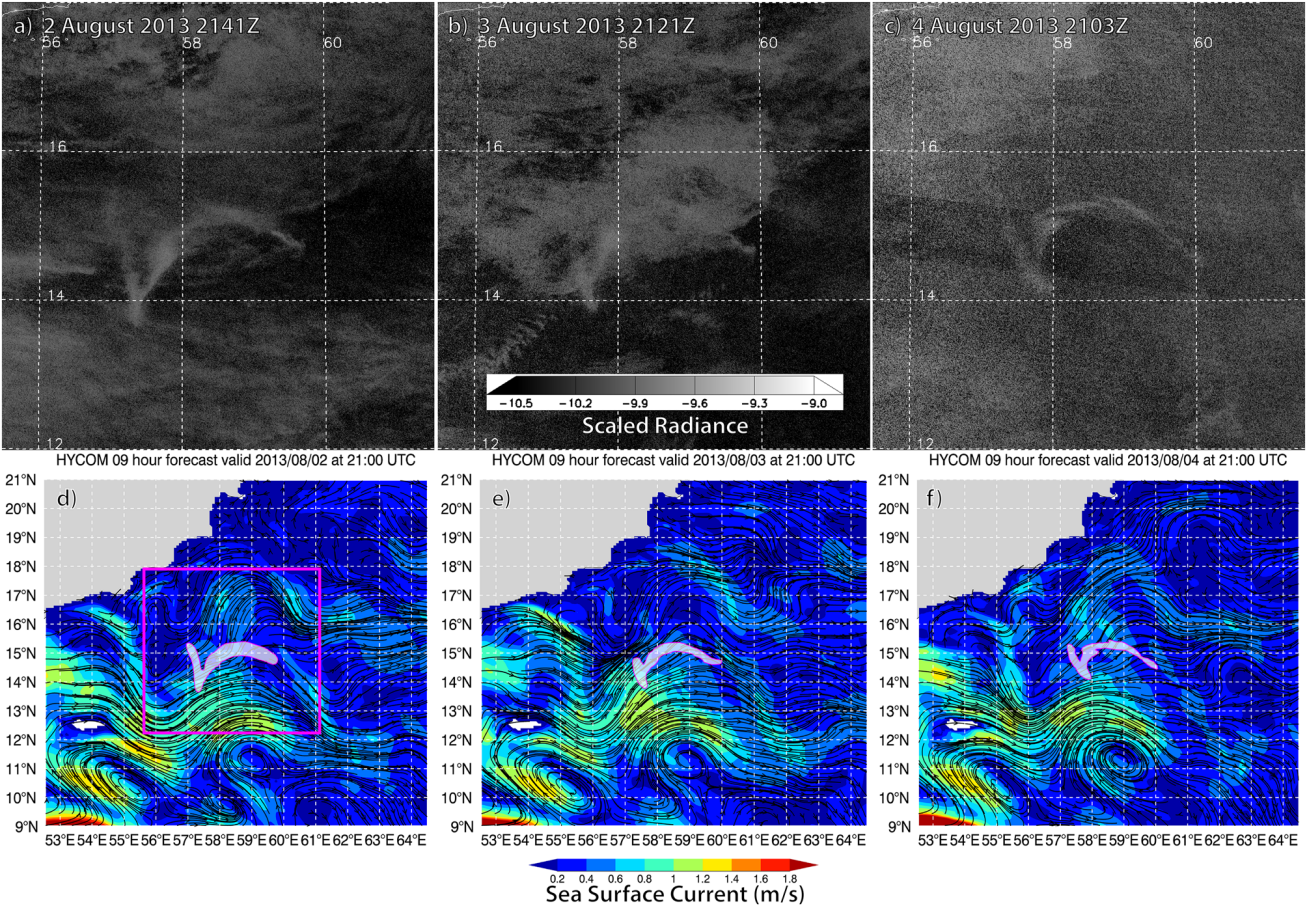
Three-night sequence over 2–4 August 2013 of a bioluminescent milky sea in the Arabian Sea for **(a–c)** DNB log_10_—scaled radiance imagery (W cm^−2^ sr^−1^), showing a ~ 9000 km^2^ luminous body persisting amidst the ephemeral cloud cover, and **(d–f)** a pan-out of HYCOM sea surface currents (magenta box in **(d)** corresponds to domain of **(a–c)**, with approximate location of the luminous body noted) shown for comparison against the body’s observed structural evolution and drift.

On 31 July 2013, the *Suomi* NPP DNB detected a luminous body with well-defined boundaries (Fig. 2), located east of Socotra in the northwest Indian Ocean, at (14.0° N, 57.0° E). Uncorrelated with the observed cloud field (Fig. 2a–c), the body drifted northeast with the currents at ~ 0.44 m s^−1^, stretching and curving in a manner consistent with the analysed ocean-surface currents (Fig. 2d–f), which showed a clockwise-rotating eddy located to its south.

Whereas DNB imagery showed only dark ocean during the daytime overpasses of the same location, these glowing waters persisted on successive nights over a two-week period. By 2 August the milky sea covered ~ 9000 km^2^ (involving roughly 5 × 10^21^ to 5 × 10^22^ luminous bacteria, per “Methods” section). The DNB lost sight of the milky sea on 14 August due to moonlight, and it was not seen again in the following moon-free period.

*Suomi* NPP’s daytime chlorophyll-a (Chla) retrievals, a proxy for the amount of organic material in the surface waters, showed structural similarities to the milky sea, but were more widespread (Supplementary Fig. S1). Moreover, the most elevated regions of Chla (> 1 mg m^−3^) occurred not directly atop, but adjacent to the most luminous waters—a recurring property among the cases documented in this research which may indicate regions of algal stress where (potentially luminous) bacteria would proliferate. On several nights, a faint signature of the luminous body was detectable beneath analyzed cloud cover; its light scattering upward through the clouds in a way similar to the behaviour of city lights in DNB imagery.

### Somali Sea, January 2018

**Figure 3:**
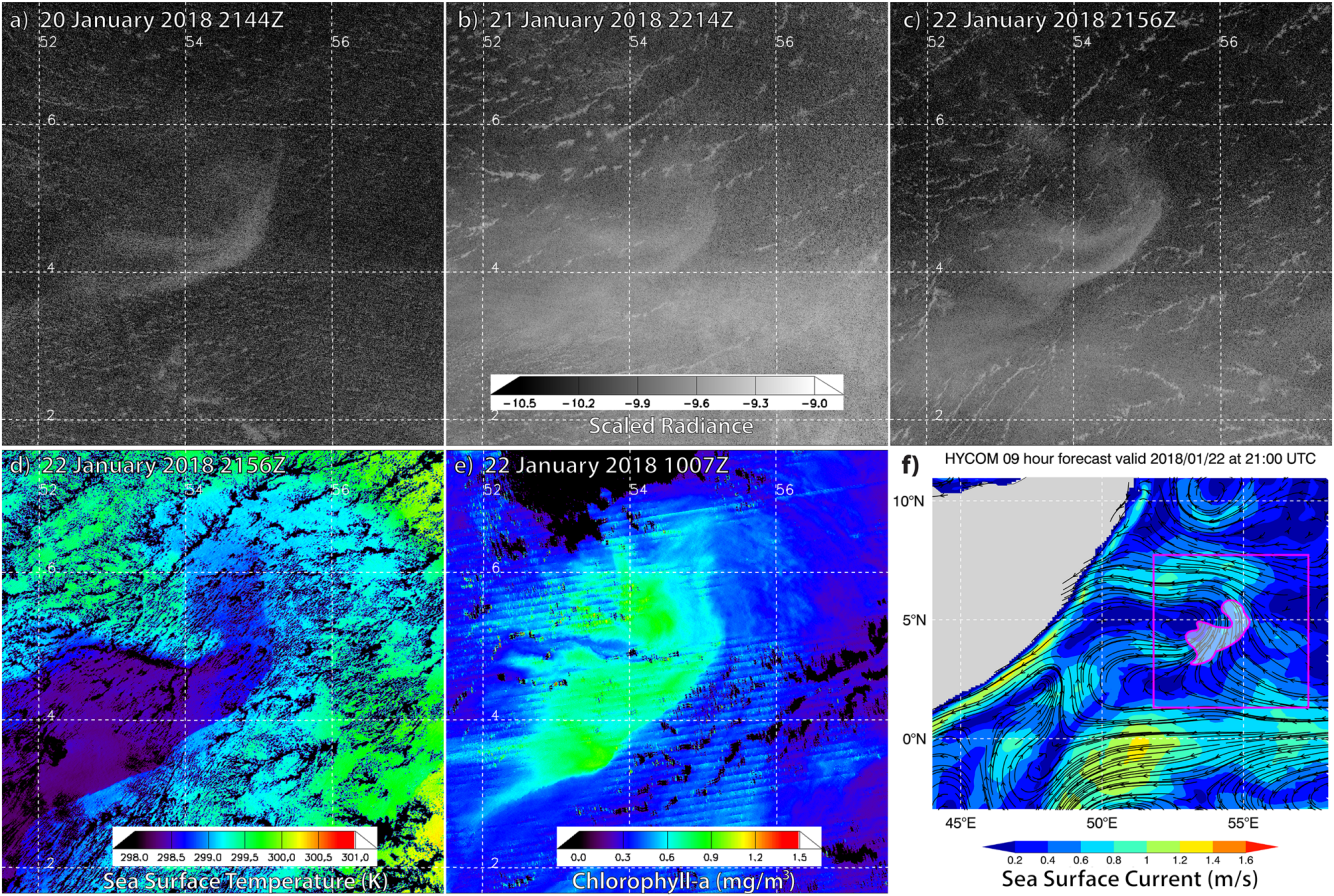
Three-night sequence over 20–22 January 2018 of a bioluminescent milky sea in the Somali Sea for **(a–c)** DNB log_10_ – scaled radiance imagery (W cm^−2^ sr^−1^), showing a ~ 15,000 km^2^ luminous feature persistent amidst the variable cloud field. Focusing on 22 January, **(d)** VIIRS-derived night time SST (K; with cloud cover in black) at 2156Z, **(e)** daytime VIIRS-retrieved Chla (mg m^−3^) at 1007Z, and **(f)** pan-out of HYCOM sea surface currents at 2100Z with approximate location of DNB-observed luminous body.

On 12 January 2018, both *Suomi* NPP and NOAA-20 captured a luminous structure offshore of southern Somalia. Over the next 5 days it stretched into a narrow filament that paralleled the Somali coast, mirroring the behaviour of other winter-mode Somali Sea cases described in Supplementary Discussion 2. Over 18–23 January, the luminous filament extended east/northeast, forming a comma-shape (Fig. 3a–c), with a sharply-defined southeastern edge and gradually fading brightness on its northwestern side. By 20 January, it spanned ~ 15,000 km^2^, suggesting involvement of roughly 8 × 10^21^ to 8 × 10^22^ bacteria. Its scale, shape, time, and location were similar to the *Lima*-sighted milky sea^4,14^, as well as to a subset of the surface reports in Supplementary Discussion 1.

Comparing the luminous body to satellite retrievals of Sea Surface Temperature (SST; Fig. 3d) showed its eastern boundary aligned with the edge of an oceanic front, residing within relatively cool (298–299 K) waters that extended northeast from the Somali coastal upwelling zone. These cooler SSTs corresponded to elevated Chla values in the range of ~ 0.5–1.0 mg m^−3^ (Fig. 3e). As in the 2013 Socotra case, the area of elevated Chla was more extensive than the luminous region. Ocean surface currents (Fig. 3f) showed the body’s eastern boundary embedded within counter-clockwise flow and drifting north/northwest at ~ 0.8 m s^−1^.

### Java, July–September 2019

**Figure 4:**
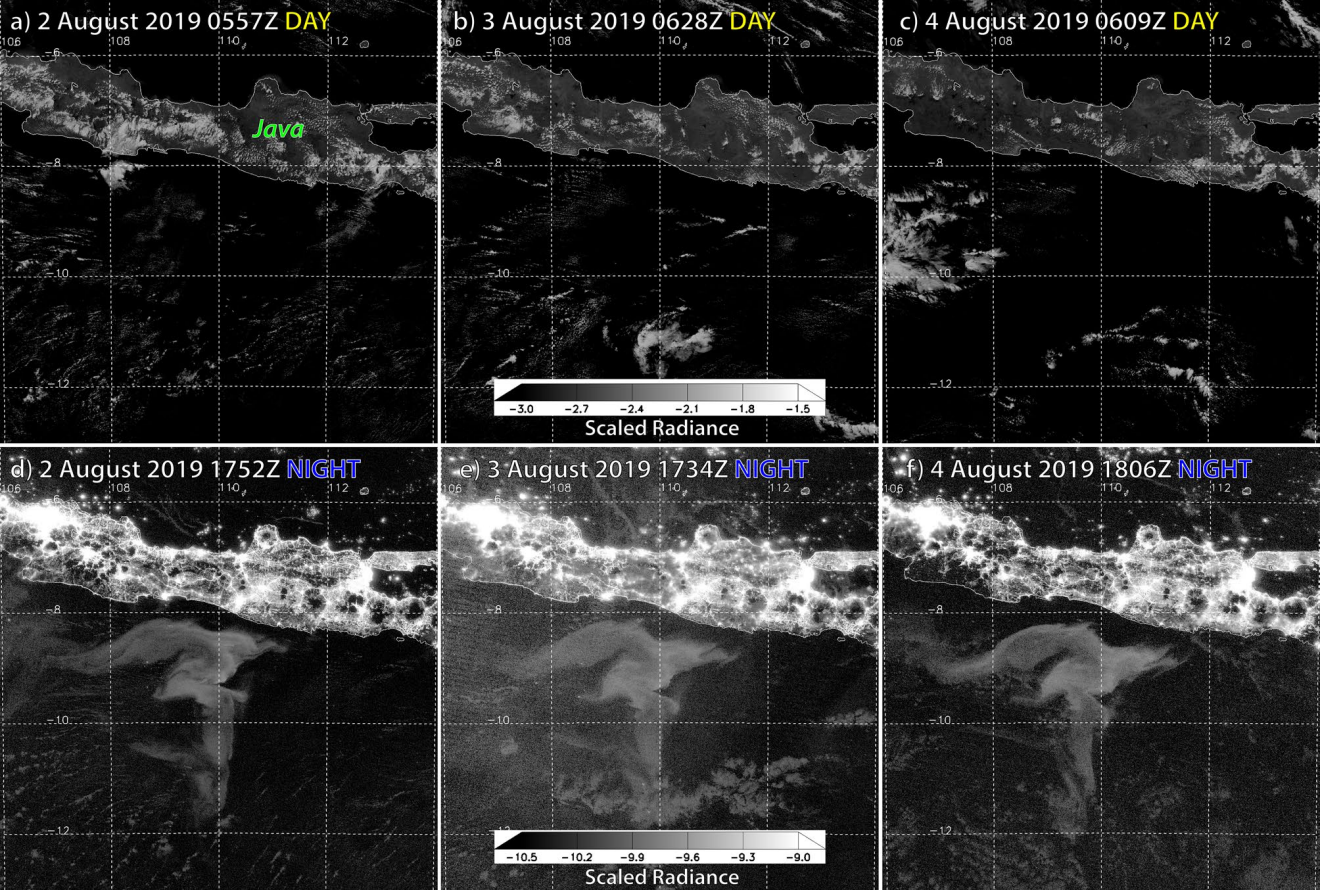
Day/night comparison of DNB log_10_—scaled radiance imagery (W cm^−2^ sr^−1^) of a bioluminescent milky sea near Java for the period 2–4 August 2019 for **(a–c)** daytime imagery, and **(d–f)** night time imagery. The amorphous luminous body, located immediately south of Java and detectable only at night, covered ~ 100,000 km^2^ of ocean surface. Bright patches seen over Java in **(d–f)** are city lights.

The DNB detected a large milky sea in the east Indian Ocean, immediately south of Java, Indonesia in 2019. The event spanned two complete moon-free cycles (26 July–9 August, and 25 August–7 September). On the night of 25 July, the DNB detected a luminous anomaly south of Surakarta, Java, near 9.5° S, 111° E. The detection amidst moderate moonlight conditions suggested a particularly strong source of emission. Imagery on subsequent moonless nights confirmed that the initial detection was in fact part of a much larger milky sea, spanning ~ 100,000 km^2^—approximately the same size as Iceland. A milky sea of this scale suggests involvement of roughly 6 × 10^22^ to 6 × 10^23^ luminous bacteria, which would qualify as the largest event on record. Undetectable during the day, the contiguous feature reappeared in nightly imagery throughout the two observable moon-free periods (Fig. 4).

Situated within quiescent, low-shear waters (Supplementary Fig. S2) between counter-clockwise-spinning warm-core eddies to its southeast and southwest (Fig. 4), this massive milky sea rotated clockwise like a cog between gears, its centre near 9.0° S, 110.0° E. By 30 July, its northern boundary approached within 25 km of the Java coast, and a 500 km^2^ area of its core was so bright that certain infrared-detected cumulus clouds appeared in the DNB imagery as dark, attenuating objects in contrast to the glowing waters below them (Supplementary Fig. S3).

The DNB radiances measured in the brightest areas of these luminous waters approached Crescent- to Quarter-Moon illumination levels. Based on scotopic vision sensitivity to bioluminescent emission (Supplementary Fig. S4) and direct comparisons against legacy OLS imagery (Supplementary Fig. S5), portions of this milky sea may have appeared visually bright to dark-adapted human vision—perhaps even attaining the classical snowfield effect described in the historical mariner accounts.

After losing sight of the luminous body on 10 August due to moonlight contamination, the DNB recaptured it on 25 August and tracked it for 2 weeks thereafter, as described in Supplementary Discussion 2. The longevity of this event, which lasted for at least 45 nights, by far eclipsed all other cases encountered in this study—indicating that significant milky seas offer a reasonable time window for reaching them if a rapid-response team is on the ready.

**Figure 5:**
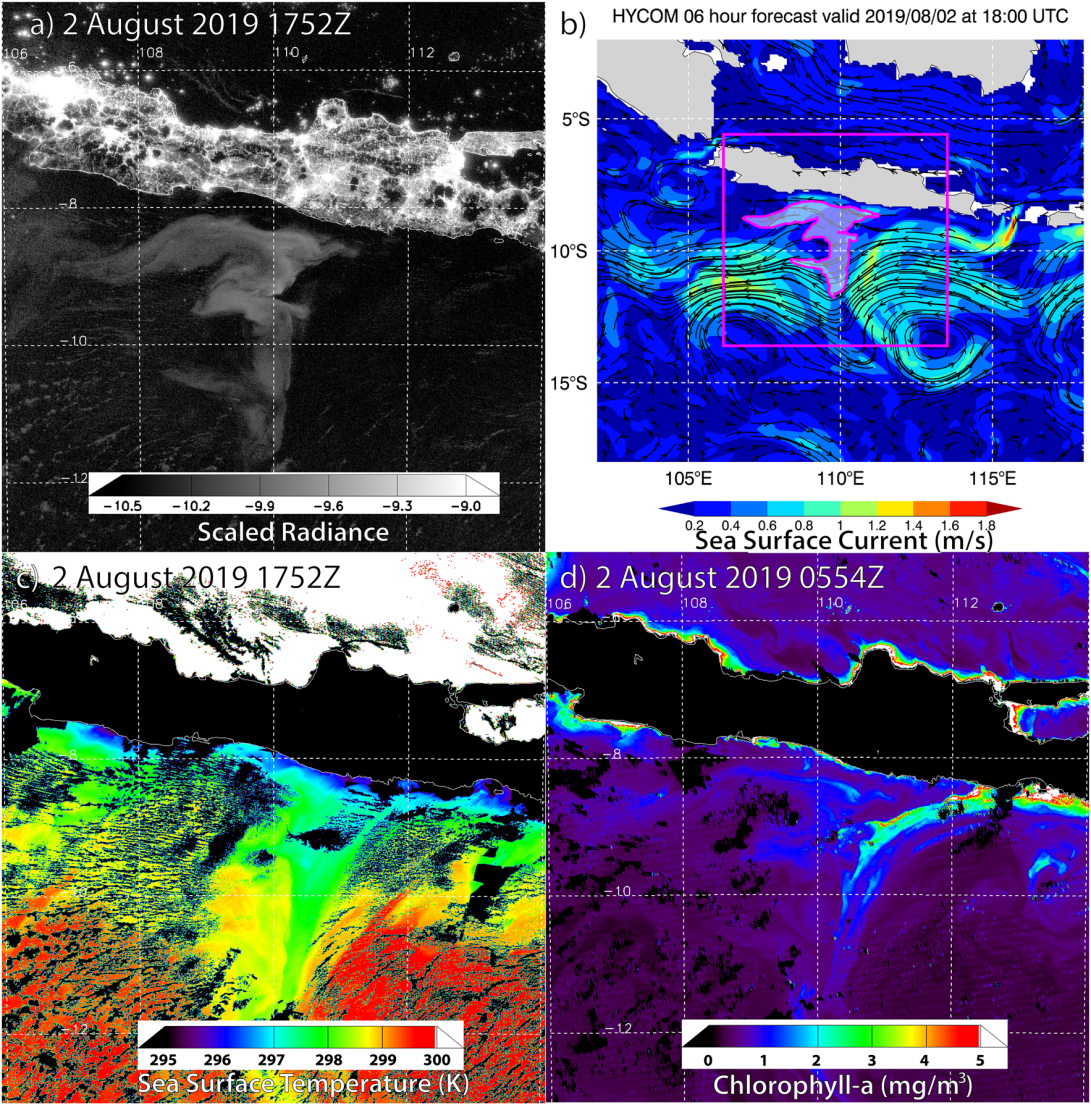
Multi-parameter analysis of the 2019 Java milky sea on 2 August 2019 for **(a)** night time DNB log_10_—scaled radiance imagery (W cm^−2^ sr^−1^) at 1752Z, **(b)** pan-out of HYCOM sea surface currents valid at 1800Z (magenta box shows domain of **(a)**, with approximate location of luminous body shaded), **(c)** VIIRS-retrieved SST (with cloud cover and land surfaces in black) valid at 1752Z, and **(d)** daytime VIIRS-retrieved Chla at 0554Z.

Satellite retrievals of ocean surface properties for the 2019 Java case (Fig. 5) showed cooler waters and elevated Chla along the Java coast. A stream of higher Chla (> 2 mg m^−3^), embedded within a tongue of these cooler waters, extended southward and immediately east of the luminous body, following the flow of the south-eastern eddy (Fig. 5b). At this time, the luminous waters were confined to a narrow range of SST over 298 ± 1 K (~ 25 ± 1 °C) and moderate Chla over 1 ± 0.5 mg m^−3^, demarcated from the surrounding waters at abrupt boundaries defined by coastal upwelling to the north and the two eddies to the south.

**Figure 6:**
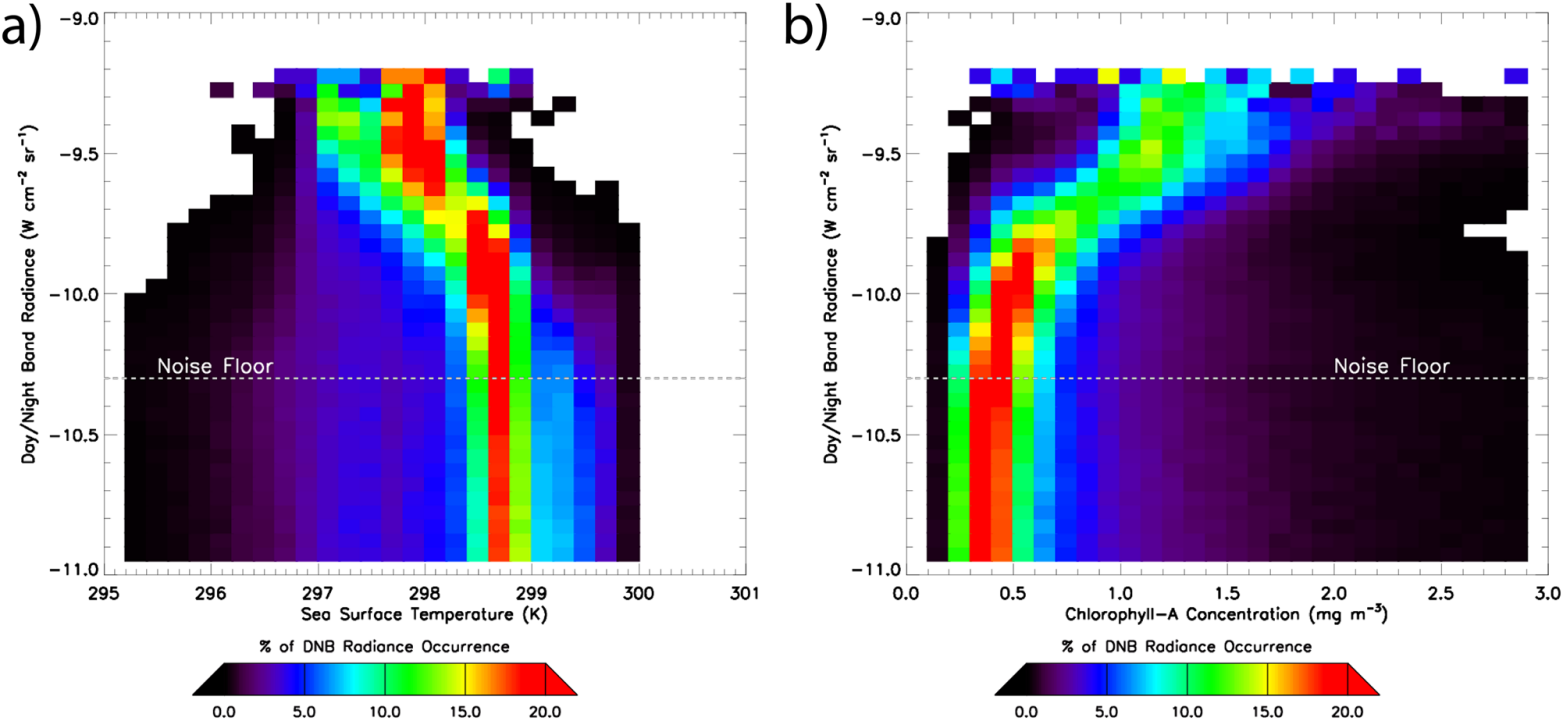
Relationship between DNB-measured milky sea radiances and ocean surface fields for the 2019 Java case (27 July–5 August, centred on the luminous body). Radiance-specific distributions (i.e., for a given radiance level, each row sums to 100%) are shown as a function of **(a)** SST and **(b)** Chla. The DNB noise floor (where SNR = 1) is drawn as a horizontal dashed line.

Figure 6 relates DNB radiance, SST, and Chla for 10 nights (27 July–5 August) of the 2019 Java case, centred on the luminous body. DNB radiances correlated positively with Chla over 0.5–1.5 mg m^−3^, and negatively with SST over 297–299 K. The brightest milky sea waters corresponded to asymptotic SST values of ~ 298 K and Chla of ~ 1.2 mg m^−3^, respectively, and notably, did not overlap with the strongest parts of the algal bloom. The SST values, hovering around 298 K for most DNB-detected cases in this study, may hold significance, as this temperature regime promotes rapid growth of *V. harveyi* and *P. leiognathi* over a wide range of ocean salinity values^20^.

## Discussion

**Figure 7:**
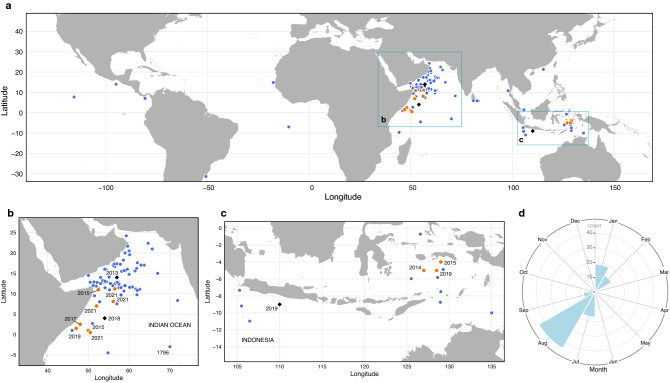
Compilation of historical (1796–2010) ship reports (Supplementary Table S1) and satellite detections of milky seas. Ship-report locations (blue) and the centroid locations of satellite-based DNB detections (black diamonds are cases highlighted in the main manuscript, and orange circles are cases presented in Supplementary Discussion 2) are shown for **(a)** global, **(b)** northwest Indian Ocean, and **(c)** Indonesia region. **(d)** Shows the temporal frequency of occurrence for all data.

Based on historical ship reports and the distribution of DNB-detected events reported here (Fig. 7), modes of peak milky sea activity in the northwest Indian Ocean resonate with the southwest (summer) and northeast (winter) monsoons^2^. Strong upwelling of cool, nutrient-rich waters along the Somali coast during these modes is understood to play an important preconditioning role for primary production. Maritime Continent region events are less common and demonstrate no clear monsoonal linkage. If these milky seas resemble their northwest Indian Ocean counterparts, other physical mechanisms promoting enhanced upwelling and primary production must be at play.

Here, the Indian Ocean Dipole (IOD^21^) offers a plausible explanation. Like the El Niño, the IOD is an aperiodic basin-scale air-sea coupled oscillation characterized by an east–west SST gradient between the western equatorial (50–70° E, 10 S–10° N) and southeastern (90–110° E, 10S–10° N) regions of the Indian Ocean, with an accompanying basin-scale solenoidal circulation in the atmosphere. The IOD begins in early-to-mid austral summer and peaks in the late-fall/early-winter. During its positive phase, the IOD corresponds to warm/wet conditions with warm pooling waters on the western side of the Indian Ocean, and cool/dry conditions with strong easterly winds on the eastern side. These winds generate upwelling of cool, nutrient-rich coastal waters which drift offshore with the currents, leading to algal blooms over a broad region^22^, and potentially, conditions favourable for milky sea genesis.

In mid-2019, the IOD was in a strong positive phase—the largest since the 1997–1998 season^23^. Associated strong upwelling throughout the eastern Indian Ocean produced Chla concentrations in the fall of 2019 that were 70–80% higher than the same period of a normal year, and the bloom lasted until the spring of 2020. These conditions may have primed the entire Indonesian region for milky sea activity. Supporting this hypothesis, concurrent to the 2019 Java event was another DNB-detected event in the Banda Sea, ~ 2000 km away (Supplementary Discussion 2). The simultaneity raises suspicion of a regional-scale causality like the IOD. The aperiodic nature of the IOD may also help to explain the relative infrequency of Maritime Continent milky sea reports compared to those of the northwest Indian Ocean, which follow an almost clock-like modality with the Indian Monsoon.

### A natural flask?

The current hypothesis for milky sea morphology—the surface-slick—cannot explain many mariner accounts of the uniform glow persisting under wind-roughened seas (where slicks would break up), light emanating from below the surface even as bucket samples of water were drawn, and the lack of a dark ship wake (which would disrupt a slick). The ensemble of accounts suggests that milky sea light can emanate from the water volume as opposed to being confined to a biofilm skin-layer.

Satellite observations and ancillary data for the DNB-identified milky seas suggest that the luminous areas corresponded to isolated water masses of uniform SST and moderate Chla—situated in the doldrums between eddies, straddling stronger adjacent currents, and aligned with the cool-side of oceanic fronts. Such waters, if bounded from below by the thermocline and laterally by density or shear layers, and if containing a blend of organic nutrients at temperature and pH levels favorable to luminous bacteria proliferation, could serve as a kind of isolated incubator for milky seas—analogous to how bacteria can be grown within the controlled environment of a flask in a laboratory.

Even a few bacteria can produce light detectable to highly sensitive photometers^24,25^, but typically they do not begin to emit such light until they reach critical populations of ~ 10^8^ cells mL^−1^; then they turn on rapidly (within hours) and emit steadily. This critical population is a proxy for autoinducer reaching concentrations sufficient to trigger bacterial quorum sensing. In a milky sea scenario, diffusion of autoinducer from super-critical bacterial populations within the confines of the natural flask could trigger a bioluminescent response from sub-critical bacterial populations nearby—producing a glowing volume of water. Alternatively, a process such as marine snow that involves bacteria-colonized organic particles could achieve the volumetric emission effect within the flask medium.

The flask hypothesis would reconcile cases where the surface-slick idea breaks down. For example, a well-mixed, isolated layer could maintain its bulk properties if it encountered a swell, or if winds roughened its surface. Since the bulk properties of a well-mixed volume would not be modified dramatically by a vessel disrupting its surface, it could maintain its steady glow in the ship’s wake as well.

### The path forward

To evaluate these process, composition, and structural hypotheses, including the natural flask as proposed here, we must be able to sample milky seas in situ across space and time. Research vessels properly equipped to collect water column measurements of temperature, organic content, chemistry, and bioluminescence production from the surface to a depth of at least 30 m, would enable these evaluations. Observations collected at various horizontal locations within, at the perimeters, and outside of a milky sea, would provide important context to the satellite imagery.

Gaining access to remote events poses an inherent challenge to advancing milky sea research. Various modes of detecting oceanographic-scale bioluminescence, including autonomous underwater vehicles (AUVs) equipped with bathyphotometers^26,27^, could enable multi-week sampling of target areas that are logistically difficult or unsafe to access and monitor on a regular basis. Deploying these assets to the right place in time will rely on our dark-adapted eyes in the sky to light the way.

## Methods

On moonless nights (for the ~ 0130 local time sun-synchronous satellite orbit, a period spanning from two nights after Last Quarter lunar phase until two nights after First Quarter), DNB data (in-band radiance units of W cm^−2^ sr^−1^) were log_10_-scaled and displayed as imagery over the range [− 10.5, − 9.0]. Nightly sequences of these images were examined for contiguous structures exceeding the background (SNR > 3) and distinct from the infrared-based VIIRS-identified cloud field.

Candidates identified as anomalous were then monitored for multi-night persistence to further distinguish them from ephemeral clouds and airglow. Daytime DNB imagery was used to confirm these same areas as being dark ocean, ruling out a reflective suspension (e.g., sediments). Motion and evolution of the luminous bodies were compared against short-term forecasts (up to 12 h) of sea surface currents from the U.S. Navy Hybrid Coordinate Ocean Model (HYCOM; www.hycom.org) model. Surface deformation fields^28^ derived from the HYCOM currents, including total strain, relative vorticity, the Okubo-Weiss parameter, and the effective Coriolis frequency, were computed to analyse the water state vis-à-vis the observed morphology of the luminous bodies and determine their relationship to surrounding waters (Supplementary Discussion 2).

For cases from 2018 onward, DNB imagery from the *Suomi* NPP and NOAA-20 satellite pair (180° opposing-azimuth views at 50 min separation) were checked for advection and parallax shift. Atmospheric features (e.g., clouds and airglow) can produce large (> 10 km) spatial displacements when viewed obliquely from opposing azimuth angles due to parallax shift and drift over the 50 min sampling interval. In contrast, ocean surface (milky sea) features drifting at < 1 m s^−1^ produce small (< 0–3 km) displacements due to zero parallax and small advection over this same interval (e.g., Supplementary Fig. S6).

Operationally-produced VIIRS retrievals of SST^29^ and Chla^30^ were used to relate the DNB-observed luminous bodies to oceanic structure (e.g., fronts and eddies) and algal blooms, respectively. When available, ship lights (also detectable by the DNB^31^) were inspected for signs of clouds (e.g., Supplementary Fig. S7); in clear-sky conditions, ship lights in DNB imagery appear as distinct point sources, while intervening clouds diffuse these lights due to multiple scattering.

Rough estimates of total bacterial cell population for milky seas assumed a quorum sensing threshold^1^ range of ~ 10^7^ to 10^8^ cells mL^−1^. This threshold range was adjusted upward by a factor 5.6 to account for the relatively lower (~ 11%) detection efficiency of the DNB band-pass to a luminous bacterial emission source^4^. Per the hypothesis of a surface slick, the estimates of total population assume that bacteria reside in a 1 cm-thick biofilm of surface water, and use the DNB-observed areal extent of the milky sea. Considering the variations among strains and species of luminous bacteria, their non-linear production rate and sensitivity to autoinducer, and stability of the autoinducer itself, these total cell population estimates should be considered as very rough.

To gauge relative sensitivity between the DNB sensor and dark-adapted (scotopic) human vision, their respective spectral response functions were convolved with a representative luminous bacterial emission spectrum (*V. fischeri*) and compared. While the human-to-DNB responsivity ratio of 4.15 does not provide a direct translation between the appearance of DNB imagery and the visual perception of a milky sea, it does indicate the potential improvement of a future DNB-like sensor optimized for bioluminescence detection.

## Supplementary Information

Supplementary Information 1.

Supplementary Information 2.

Supplementary Information 3.

Supplementary Information 4.

Supplementary Information 5.

Supplementary Information 6.

## Data Availability

Satellite data supporting this research are available from the NOAA Comprehensive Large Array-data Stewardship System (CLASS; www.class.noaa.gov). Processed data supporting the findings of this study are included in Supplementary Discussion 2.
