# Supplementary material for: Honing in on bioluminescent milky seas from space
Source: Sci Rep. 2021 Jul 29;11:15443. doi: 10.1038/s41598-021-94823-z (PMC8322353; doi:10.1038/s41598-021-94823-z)
Supplement: Supplementary file 1 — Supplementary Information 1. [file 41598_2021_94823_MOESM1_ESM.docx]

**Supplementary Discussion 1: *Milky Sea* *Eye Witness Accounts***

Table of Contents

[1. Introduction 1](#_Toc74928002)

[2. Chronology of Reported Milky Sea Sightings 2](#_Toc74928003)

[2.1 1864 Somali Sea 2](#_Toc74928004)

[2.2 1968 East Central Pacific 3](#_Toc74928005)

[2.3 1972 Somali Sea 3](#_Toc74928006)

[2.4 1980 Arabian Sea 4](#_Toc74928007)

[2.5 1981/82 Gulf of Thailand 5](#_Toc74928008)

[2.6 1982 Gulf of Oman 5](#_Toc74928009)

[2.7 1984 Arabian Sea 6](#_Toc74928010)

[2.8 1990 West Indian Ocean 6](#_Toc74928011)

[2.9 1991 Arafura Sea, Maritime Continent 7](#_Toc74928012)

[2.10 1992 Banda Sea, Maritime Continent 7](#_Toc74928013)

[2.11 1995 Banda Sea, Maritime Continent 8](#_Toc74928014)

[2.12 Andaman Sea, Maritime Continent 8](#_Toc74928015)

[2.13 1999 East Pacific 9](#_Toc74928016)

[2.14 2000 Gulf of Aden, Arabian Sea 9](#_Toc74928017)

[2.15 2002 Arabian Sea 10](#_Toc74928018)

[2.16 2002 East Pacific 11](#_Toc74928019)

[2.17 2002 Gulf of Aden, Arabian Sea 11](#_Toc74928020)

[2.18 2010 Arabian Sea 11](#_Toc74928021)

[3. Summary 12](#_Toc74928028)

# Introduction

This Supplemental document contains eyewitness encounters with possible milky seas and/or other unusual bioluminescent phenomena. The reports were brought to our attention by members of the maritime community, both recreational and professional. Informed consent was obtained to publish these accounts in an online open-access publication. Some reports are contemporary, and others occurred many years ago but were either never recorded or were embedded within broader topics. For example, a milky sea encounter logged by the Captain of the Confederate Steam Ship (CSS) *Alabama* is recounted here despite its original inclusion as part of the *Project Gutenberg*; the account itself was buried deep within an ~800 page memoir.

The sources vary widely in background, ranging from private citizens to Naval Officers. All must be considered as subjective, anecdotal, and the possibility of fabrication in some cases cannot be ruled out fully. Many share similar characteristics, both amongst each other, with previously reported encounters. In other cases, new and unique details emerged, helping to build upon our currently limited understanding of milky sea structure, and providing an opportunity to form new/refined hypotheses. The reports are listed here in chronological order. Best available information on date and location of each sighting, when conveyed and by whom, is followed by an abridged description of the encounters, appended by our interpretations and commentary where applicable.

# Chronology of Reported Milky Sea Sightings

## 2.1 1864 Somali Sea

**Date:** 30 January 1864

**Location:** Somalia ~(2.72 N, 51 E)

**Reporter:**  Capt. Raphael Semmes (CSS *Alabama*), *Memoirs of Service Afloat, During the War Between the States*, p. 732

**Account:** At about eight P. M., there being no moon, but the sky being clear, and the stars shining brightly, we suddenly passed from the deep blue water in which we had been sailing, into a patch of water so white that it startled me; so much did it appear like a shoal. (…) The patch was extensive. We were several hours in running through it. Around the horizon there was a subdued glare, or flush, as though there were a distant illumination going on, whilst overhead there was a lurid, dark sky, in which the stars paled. The whole face of nature seemed changed, and with but little stretch of the imagination, the *Alabama* might have been conceived to be a phantom ship, lighted up by the sickly and unearthly glare of a phantom sea, and gliding on under the pale stars one knew not whither.

**Notes:** Several (~3) hours of traverse at 8 kt translates to ~45 km (or ~27 miles)—consistent with the Captain’s reference to an area ~20 miles square. Its timing and location compares to S.S. *Lima* in 1995 (Miller et al., 2005), and more recent events detected by the DNB, and fits squarely within the northeast monsoonal (wintertime) mode of milky sea sightings.

## 2.2 1968 East Central Pacific

**Date:** 1968, Midnight-4 AM local time

**Location:** Azuero Peninsula, Panama, Pacific side ~(7.0 N, 80.7 W)

**Reporter:** *Anonymous,* US Navy (USN)

**Account:** About a mile from me off to starboard, the sea was suddenly so bright and glowing that it attracted my attention. The glow just kept growing larger, and it was soon apparent that it would engulf the sea in front of my bow, even though I was moving away at 10 knots. The milky glow expanded and got beneath our hull and kept right on growing. From at least a mile to starboard of us, and to a mile to port from us, the sea in all directions was glowing. We just drifted for about 30 minutes with this glow beneath us. We hauled in a few buckets of water, to see what we could see, but nothing special. Then it started to dim, and in about 5 minutes it was no longer glowing at all. It didn’t move off; it just turned itself off.

**Notes:** A rare East Pacific sighting. It is among the few reports that describes a transient and migratory behavior of steady/uniform glow; in most milky sea reports the ships cross in/out of relatively stationary glowing waters. It is unclear whether the event was indeed a milky sea that involved a rapid expansion of quorum sensing, or some other phenomenon.

## 2.3 1972 Somali Sea

**Date of Sighting: ~**Jan/Feb 1972

**Location:** Offshore of Somalia, near equator ~(0 N, 54 E)

**Reporter:** Capt. Kazim Albayrak, (relayed by his son, Murat Albayrak)

**Account:** My mother saw my father rushing down from the bridge into their cabin, flipping excitedly  through various books and asked if something was wrong. My father simply said “*look through the porthole.*” When she peered outside the sea was milky white as far as the eye could see. There was no moon to confuse what they saw. They were sailing in a sea of milk. They took samples from the sea to see if it was a bioluminescence of some sort and if the water in the bucket would also be the same color. It was quite the same ordinary old sea when in the bucket. This situation lasted until the morning and they never experienced it again.

**Notes:** A classic milky sea description, occurring in a region and at a time-of-year of historically high frequency (wintertime northeast monsoon) for the this region as reported by Herring and Watson (1993). The location and timing of this event holds similarities with the CSS *Alabama* (1864) account, the S.S. *Lima* (1995) account, and several more recent DNB observations reported by the current manuscript.

## 2.4 1980 Arabian Sea

**Date of Sighting:**  ~Late May - Early June, 1980, ~7:30-11:30 PM local time

**Location:** Arabian Sea, near Socotra

**Reporters:** Lt. Chris Tolton & Reid Hinson (both USN, Retired)

**Accounts:** *Tolton*: (At) about 1930 – 2030 hrs, Reid Hinson announced something unusual out on the seas. Much of the crew went out on deck and we were amazed at what we saw. It was night already and dark. It was like we were in the "*Twilight Zone*" and peering at a negative of the real world. The seas were glowing with phosphorescence as far as you could see all around us. The ship was darker than the seas, the sky was darker than the seas (normally the seas are the darkest of all). The phosphorescence was uniform and a bit lighter green or "whiter" than the normal screw-generated green phosphorescence (kind of like the glow-in-the-dark plastic stars you can buy your kids).  There were no breaks in the phosphorescence even with the waves, i.e., I didn't see any "holes" of dark water but the wave foam was dark against the glowing water.  I don't know how deep it went, but it appeared to be deeper than just the surface water - more than several yards deep.

*Hinson*: My impression at that time was that there was a very low level of fog, the top of which was about 10 ft above the water.  The fog, coupled with fairly intense bioluminescence, looked like an illuminated blanket that was quite remarkable, almost surreal.

**Notes:** Observation of the foam upon the water appearing dark in contrast to the glowing water is consistent with the 1995 S*.S. Lima’s* description of kelp appearing black against the bright water. We checked the deck logs from USS *O’Brien* in the Naval archives but could not find an official entry logged for this encounter. It is not clear whether the perception of a fog layer was an optical illusion caused by the glowing surface, but the descriptions of light-attenuating seafoam and light emanating from depth contradicts the surface slick hypothesis for milky seas.

## 2.5 1981/82 Gulf of Thailand

**Date of Sighting:** Between 11/1981 - 12/1982

**Location:** Gulf of Thailand

**Reporter:** David Globehill (private mariner)

**Account:** The most striking event that I witnessed was the overall sea being a bright emerald green, electric blue at the crests of the waves (formed by the ship's wake/bow wave) with what I can only describe as a giant rotating helicopter blade affect rotating astern and either side of the vessel. It seemed to be resonant with the ship's propeller. Also within the flat calm sea there were frequent pin points of electric blue light.

**Notes:** This appears to be a combination of a classic milky sea (strong enough to be perceived in color) coupled with dinoflagellate-associated “phosphorescent wheel” effect.

## 2.6 1982 Gulf of Oman

**Date of Sighting:** 1982

**Location:** Gulf of Oman

**Reporter:**  Mr. Hywell Phillips, Royal Navy

**Account:** During 50 years on British military ships, he witnessed a milky sea per the classic description in 1982 in the Gulf of Oman.

**Notes:** Unfortunately, we missed our opportunity to speak with Mr. Phillips directly about his experiences. However, based on his long term of service in the Royal Navy, and additional descriptions he provided of separate encounters with phosphorescent wheels, we take his accounts as credible.

## 2.7 1984 Arabian Sea

**Date of Sighting:**  July/August 1984, 2200 – Midnight Local Time

**Location:** Off Masirah Island, offshore of Oman, Arabian Sea ~(20.5 N, 60 E)

**Reporter:**  Kris Kimmons (USN, Retired)

**Account:** One night the surface of the sea was phosphorescing brilliantly enough you could read (barely) on the bridge. The extent of the phenomenon was from horizon to horizon. Of note, we were working with a pilot who reported he was seeing it from horizon to horizon as well at 1000 feet. (The sea surface) had a very diffuse look. The spray (~15-20 kts blowing and ~3 foot chop) was phosphorescing as well so the surface looked like a soft fuzzy blanket. The light from the surface of the sea was brighter than full moonlight. The whole battle group was in the phosphorescent area. I went to bed and asked the other watch and they said it lasted pretty much the rest of the night.

**Notes:** A classic milky sea description, given its steady, diffuse, widespread and sustained appearance. It occurred during summer monsoon mode within the documented high-frequency sighting region of the northwest Indian Ocean. This is the only milky sea account that includes a simultaneous aircraft-based observation—which may be an important element of any future rapid-response deployment to a satellite-detected milky sea.

## 2.8 1990 West Indian Ocean

**Date of Sighting:**  ~Apr-Jun 1990

**Location:** Indian Ocean near Equator, east of Africa near Seychelles

**Reporter:** CDR Thomas Evanoff (USN, Retired)

**Account:** (The milky sea) went on for miles. It was so bright I could read my notebook while standing on the bridge wing. At the time, I was the ship's navigator and happened to have the deck watch that night. It was so unusual, I remember calling the Captain and having some crewmen come up from below deck to observe. The observation was near the Equator, well east of Africa in Indian Ocean, during transit to/from port visit in Seychelles.

**Notes:** Of particular interest is the April-June timeframe of this event, which places it off-mode with regard to the winter and summer Indian monsoons. The reference to being bright enough to read by from the ship deck is consistent with several with other eye-witness accounts of perceived milky sea visual brightness.

## 2.9 1991 Arafura Sea, Maritime Continent

**Date of Sighting:**  First Week of August 1991

**Location:** Off coast of New Guinea, Arafura Sea ~(10S, 135E)

**Reporter:** Joyce and Don Green (private mariners of the yacht *Windy Thoughts*)

**Account:** Suddenly, my senses were overcome with the most astounding sight! The sea was alive with a vast bright green glow of bioluminescence that was often experienced in the boat's wake---but this was an entirely new phenomenon. *Windy Thoughts* sailed into a sea that was literally alight from horizon to horizon with a bright luminous green glow as far as the eye could see. Amazingly, the sky around us was aglow as well. All was dead quiet, the wind and seas calmed considerably. And yet our speed increased as *Windy Thoughts* sailed along in silence, not a sound of a wave breaking on her hull, not even the sound of the bow wave as she cut through the water---and not a whisper of wind was heard. It was surreal.

**Notes:** A classic milky sea description, with color perception suggesting a particularly strong emission source. The suddenness of the milky sea’s appearance suggests that the vessel may have crossed a relatively sharp boundary between non-luminous and milky-sea waters (i.e., an oceanic frontal structure).

## 2.10 1992 Banda Sea, Maritime Continent

**Date of Sighting:**  ~27 July 1992

**Location:** Eastern Banda Sea ~(7.5 S, 129 E)

**Date of Filing:** 24 Sep 2016

**Reporter:**  Terry Cosgrove (private mariner)

**Account:** (During the) midnight to 4 AM watch the sea was glowing with a milky grey light which reflected off the cloud cover giving an illusion of being in a fog where there was no horizon - the fact that the sea was quite lumpy (2 to 2.5 meter swell which we couldn't see, only feel) made the whole experience quite surreal. This happened over two nights on the third night the luminescence was still there but only very faintly.

**Notes:** The event presents as a milky sea, but the milky-gray appearance of the water suggests a weaker light emission than the bright-white or even blue/green appearance of other accounts. The two nights of traverse implies the milky sea occupied a large portion of the eastern Banda Sea, indeed, with its more intense portion to the south (fading away as they traveled north). This description implies the luminous body may have been aligned with an oceanic front on its southern boundary.

## 2.11 1995 Banda Sea, Maritime Continent

**Date of Sighting:**  18 July 1995

**Location:** Banda Sea (5.827 S, 128.628 E)

**Reporter:** Stuart and Ann Yellen (private mariners of the yacht *Annie’s Song*)

**Account:** At around 2000 local time (…) we sailed into a totally white sea. It extended as far as we could see and the glow reflected up into the sky, obscuring the horizon. Even the sails were hard to distinguish. Porpoises swimming by were black shapes in the water. The brightness and the disorienting nature of the experience was very tiring on our eyes. It had disappeared by 0050 July 19. It wasn't as though we left it behind but that the white light was gradually turned off. We went from white to normal black in about 15 minutes.

**Notes:** This milky sea was in the east/central Banda Sea, and their traverse was about 50 km. The southern entry was a sharper front and their exit was a gradual fade, suggesting the milky sea may have been associated with an oceanic front, as in the 1992 Banda Sea account (2.10).

## 2.12 Andaman Sea, Maritime Continent

**Date of Sighting:**  7 March 1998

**Location:** Mergui Archipelago, Andaman Sea ~(10.8 S, 97.9 E)

**Reporter:** Robert Cogen (private mariner)

**Account:** On 7 March, 1998 we were sailing through the Mergui Archipelago. We stopped in the late afternoon in the Southeast Bay of Clara Island. We had seen considerable bioluminescence the night before in the Salet Galet at the western end of Lampi Island, but on 7 March it was pervasive, white, and continuous to the horizon after dark. The following night at Black Rock, 97 degrees 35' East and 11 degrees 22' North we saw it again.

**Notes:** A coastal zone event which matches the classic milky sea description in the high-frequency Maritime Continent region of sightings. The multi-night observation is also consistent with the satellite-detected cases of the current study.

## 2.13 1999 East Pacific

**Date of Sighting:** July 1999

**Location:** East Pacific, between Panama and Acapulco

**Reporter:** Capt. Chris Nolan, US Coast Guard

**Account:** In 1999, when I served as a cadet in the eastern Pacific, the Coast Guard cutter *Chase* sailed through an entire glowing surface of bioluminescence. The ocean, as far as you could see, looked like there were dive lights underneath the ship, shining up. Steady, greenish-white water. We steamed through for maybe twenty or thirty minutes. Two huge pools of it. My only memory at this point is a flat calm sea, motoring to the north and for a good 20 minutes.  Definitely not normal bioluminescence…it was solid to the horizon.

**Notes:** A rare East Pacific report, with color perception suggesting a stronger emission source. Two distinct “pools” is an unusual aspect of this account, although the reporter could not recall if they occurred on the same night or were observed on consecutive nights (and thus, a single persistent pool).

## 2.14 2000 Gulf of Aden, Arabian Sea

**Date of Sighting:** 24-25 February 2000

**Location:** Gulf of Aden, offshore of Al Mukallah, Yemen ~(14.5 N, 50 E)

**Reporter:** Bjorn Endresen and Merete Askvik (private mariners)

**Account:** I (Bjorn) was heading southeast on my way from Salalah, Oman to Al Mukallah, Yemen. One night we encountered the milky sea. (Merete) saw it first – she was on watch – and her initial thought was that the boat had caught fire on the inside, in the forepeak - and that the fire somehow lit up the ocean. It was not ordinary bioluminescence–I grew up in Norway and had seen that thousands of times before—it was a white, light, glowing ocean around us as far as we could see. The phenomenon didn't last very long, maybe an hour or two if I recall correctly. We definitely sailed into it. We also sailed out of it - the light didn't go out, it wasn't dawn, we just left the area where the ocean was lit. We do not remember any dark wake. I think we would have remembered it if we had seen it – as it would have been the opposite of most "normal" bioluminescence, where the wake is glowing. I have sailed about 35,000 miles and have only seen it that one time.

**Notes:** A classic milky sea description that comes from experienced sailors who are familiar with the more commonly encountered forms of marine bioluminescence (i.e., dinoflagellates). This event was observed close to shore (uncommon) and in a relatively narrow (~50 km; based on a 2 hr traverse at 7 kt) band along their direction of travel, but still extensive enough to produce the pan-horizon glow effect.

## 2.15 2002 Arabian Sea

**Date of Sighting:** 11 or 12 Feb 2002, 2100 – 0000 local time (16-19Z)

**Location:** Western Arabian Sea near India ~(8.35 N, 71.90 E)

**Reporter:** Tony Johnson (private mariner)

**Account:** I came up out of the cabin to take a look around a couple of nights before making landfall in Oman, and the whole sea was white, from horizon to horizon. It was a dark night and it was hard to tell what you were looking at, but it seemed as though the boat was flying gently on the top of a cloud. (Others) also made out dark shadows moving in the water, which (they) figured were fish.

**Notes:** Perception of dark shadows moving under water (possibly fish) may provide insight on the depth of the observed light emission. If the moving shapes were indeed fish near, but not at the surface, then their dark appearance implies a significant portion of the light came from below their swimming level. The leading hypothesis for milky seas is bacterial colonization on a surface slick/biofilm, but the current report (and others) suggests otherwise.

## 2.16 2002 East Pacific

**Date of Sighting:**  8 May 2002, 4-6 AM local

**Location:** East Pacific (7.7278 N, 115.6087 W)

**Date of Filing:** 18 June 2011

**Reporter:** Jeff Bowers (private mariner)

**Account:** We sailed for half an hour through (a milky sea). I was on watch, and during a routine check was alarmed to see white water ahead. As we approached, it was apparent that the seas were just illuminated; there was no breaking water or reef. We sailed for half an hour with white water for as far as I could see. Later we sailed out of it, with a line of white water behind.

**Notes:** A classic milky sea description from a region where they are not often observed. Based on the vessel’s and duration of observation, the patch traversed was ~6 - 7 km wide, with sharply defined boundaries on entry and exit.

## 2.17 2002 Gulf of Aden, Arabian Sea

**Date of Sighting:** Sep-Nov 2002

**Location:** Gulf of Aden/Arabian Sea, Gulf of Oman, Persian/Arabian Gulf

**Reporter:** CDR Thomas Evanoff (USN, Retired)

**Account:** Two observations between Sep-Nov 2002.  One occurred in the Gulf of Aden/Arabian Sea area.  The other was in the Gulf of Oman/Persian Gulf area.  These were not as bright, or cover as large an area as the 1990 event, but they did go on for several miles.

**Notes:** These locations and period of sighting correspond to known high-frequency areas for the northwest Indian Ocean milky seas during the latter portion of the summertime southeast monsoonal mode.

## 2.18 2010 Arabian Sea

**Date of Sighting:**  11-12 August 2010 / 2200-0100 local

**Location:** Arabian Sea ~(14.1 N, 65.9 E)

**Reporter:** Sam Scott and Abigail Alling (private mariners, S/V *Mir*)

**Account:**  (We) were on watch just after dinner, and for the previous few nights it had been pitch-dark during this time of our watch, but we couldn’t help but notice that it was strangely light out. No moon, cloudy above, so no stars either, why did the world seem to be glowing? The light gradually grew stronger, and it became clear that it was coming from the water. The whole sea around us seemed to be thick and opaque like milk, and was emitting a greenish-blue light. This was not your typical bioluminescence that is little sparkles of light caused by disturbances in the water. This was a constant glow that stretched from horizon to horizon.

Within this overwhelming glow would occasionally float past large (tubular) pieces of solid material that glowed even brighter than the rest, and when I attempted to catch them in a bucket or with a gaff hook they would just break apart like clumps of dirt. We floated in (this) sea for over three hours, and everyone was in disbelief at what we were seeing, and then suddenly we could see a line of black water ahead, and as quickly as it had started we passed out of (it), but for a little while still we could see an eerie glow behind us.

**Notes:** Location approximated from noon fix, average heading/speed over 12 hr extrapolated to position at local midnight. The report, matching the description of a milky sea, notes a gradual brightening, as opposed to a sharp front, upon entry, followed by a sharp discontinuity in brightness upon exit. This description is consistent with the satellite-observed luminous structures associated with oceanic fronts, which produce a sharp boundary at the frontal boundary and a gradual fading away from it. There was an interesting comment about brighter patches appearing tubular and breaking apart “*like clumps of dirt.*” There is a kind of planktonic organism, the Pyrosome, that lives in as a colony that takes on the shape of a tube. They are known to be bioluminescent, but it is not clear how they alone could account for the widespread and unbroken glow that was observed.

# Summary

Figure 6 of the main manuscript summarizes milky sea events based on the available historical reports and current observations from the DNB. The surface reported locations do not represent the full spatial extent of the events encountered. Likewise, the Day/Night Band points correspond to the centroids of the multi-day/week and drifting luminous bodies (per *Supplementary Discussion 2*) which in some cases exceeded 100,000 km^2^. As additional satellite detections occur, a more representative climatology will become possible—possibly revealing structure patterns that provide additional insights into the relationship between milky seas and other oceanographic parameters of relevance to their formation processes.

It is possible that there are more milky sea encounters yet to be revealed. Our hope is that this latest correspondence will help to uncover any additional historical sightings that can add to the record and be verified by low-light visible satellite observations. In modern times of rapid global communication, we may hope to be alerted to events in-progress, allowing for real-time satellite confirmation or even in situ observations. Recent contacts made with merchant vessel operators (*Supplementary Discussion 2, Section 7.2*) may bear such fruit in the future. Several of the locations mentioned in these accounts have yet to be examined in a comprehensive way from the archives of DNB imagery. These satellite data may reveal additional hot-beds for milky sea activity in remote waters across the world’s ocean.
