# Supplementary material for: Honing in on bioluminescent milky seas from space
Source: Sci Rep. 2021 Jul 29;11:15443. doi: 10.1038/s41598-021-94823-z (PMC8322353; doi:10.1038/s41598-021-94823-z)
Supplement: Supplementary file 2 — Supplementary Information 2. [file 41598_2021_94823_MOESM2_ESM.docx]

**Supplementary Discussion 2: *Day/Night Band-Detected Milky Sea Cases in the Northwest Indian Ocean and Maritime Continent; December 2012 – March 2021***

The following is a compendium of milky seas detected by Suomi-NPP and NOAA-20 VIIRS DNB satellite sensors during the survey period of December 2012 – March 2021 in the northwest Indian Ocean and in the east Indian Ocean around the Maritime Continent. These cases satisfied the criteria for being ocean-surface-based light emissions, as described in *Methods* of the main paper. The case descriptions are based on in-depth analyses of sea surface currents from the U.S. Navy Hybrid Coordinate Ocean Model (HYCOM) and satellite retrievals of Sea Surface Temperature (SST) and Chlorophyll-a (Chla). Annotated DNB animations for all milky sea cases are provided as Supplementary Movies 1-12.

Table of Contents

[1. Socotra, 31 July – 13 August 2013 2](#_Toc70362847)

[2. Banda Sea, 20-24 August 2014 2](#_Toc70362848)

[3. Somali Sea, 15-28 January 2015 4](#_Toc70362849)

[3.1 Phase 1: 15-20 January 2015 4](#_Toc70362850)

[3.2 Phase 2: 21-26 January 2015 7](#_Toc70362851)

[4. Banda Sea, 12-18 August 2015 10](#_Toc70362852)

[5. Socotra (Guardafui Channel), 7-20 September 2015 12](#_Toc70362853)

[5.1 Phase 1: 7-11 September 2015 12](#_Toc70362854)

[5.2 Phase 2: 12-20 September 12](#_Toc70362855)

[6. Somali Sea, 21-31 January 2017 17](#_Toc70362856)

[7. Somali Sea, 12-23 January 2018 20](#_Toc70362857)

[7.1 Phase 1: 12-19 January 2018 20](#_Toc70362858)

[7.2 Phase 2: 19-23 January 2018 23](#_Toc70362859)

[8. Somali Sea, 28 January – 7 February 2019 25](#_Toc70362860)

[9. Java Sea, July-August 2019 28](#_Toc70362861)

[9.1 Java Sea Phase 1: 25 July – 9 August 2019 28](#_Toc70362862)

[9.2 Java Sea Phase 2: 25 August – 7 September 2019 38](#_Toc70362863)

[10. Banda Sea , 26 July - 4 August 2019 42](#_Toc70362864)

[11. Socotra and Somali Sea , 7-22 January 2021 44](#_Toc70362865)

[12. Socotra, 7 - 20 February 2021 47](#_Toc70362866)

[13. Summary 50](#_Toc70362867)

# 1. Socotra, 31 July – 13 August 2013

Model currents information provided evidence for the luminous body’s association with the ocean surface, which tracked northeastward and curved with the streamlines of an eddy to its south. Based on its observed structure, the luminous body may have initially formed within the southern area of a weakly counter-clockwise rotating doldrums offshore of southern Oman at (14° N, 56° E), before subsequently becoming entrained within stronger eastward flow associated with the southern eddy.

*
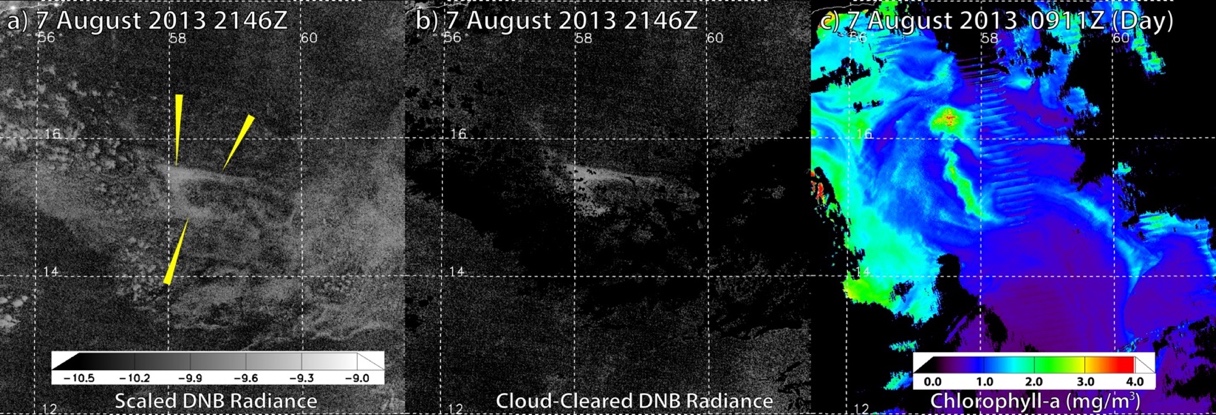
*

*Figure S2.1.1. 2013 Somali Sea milky sea as seen on 7 August. a) DNB scaled radiances (with luminous body denoted by yellow pointers. b) same as (a) but with infrared-identified cloud cover masked to black. c) VIIRS-retrieved Chla from ~12 hr earlier in that day, showing a similarly curved structure close to the luminous body.*

Extensive cloud cover over the northern Arabian Sea region precluded nighttime SST information. In fact, on several nights the steady and slowly drifting luminous feature was detectable in DNB imagery based on light scattering through the ephemeral clouds. Daytime overpasses of Suomi NPP observed only limited portions of the area under clear skies, allowing for Chla retrievals. An example from 7 August 2013 is shown in Figure S2.1.1. These retrievals revealed a region of elevated Chla values (~1.0-1.5 mg/m^3^) that resembled the shape of the luminous feature seen in nighttime DNB imagery. However, the luminous body did not correspond to the brightest Chla values, which exceeded 3-4 mg/m^3^ over significant portions of the domain. The association between luminous body and moderate values of Chla, and often adjacent to larger values of Chla, was a recurrent finding in cases encountered in this study.

# 2. Banda Sea, 20-24 August 2014

On 20 August, a comma-shaped luminous body appeared in DNB imagery, centered near (5° S, 127° E). It drifted eastward over four consecutive nights before fading from view after 24 August. Figure S2.2.1 shows the body on 20-21 August, when it was most prominent. The emission of this body was very close to the DNB noise floor. Thus it required multi-night tracking to detect its presence with confidence amidst the complex meteorological cloud field.

**
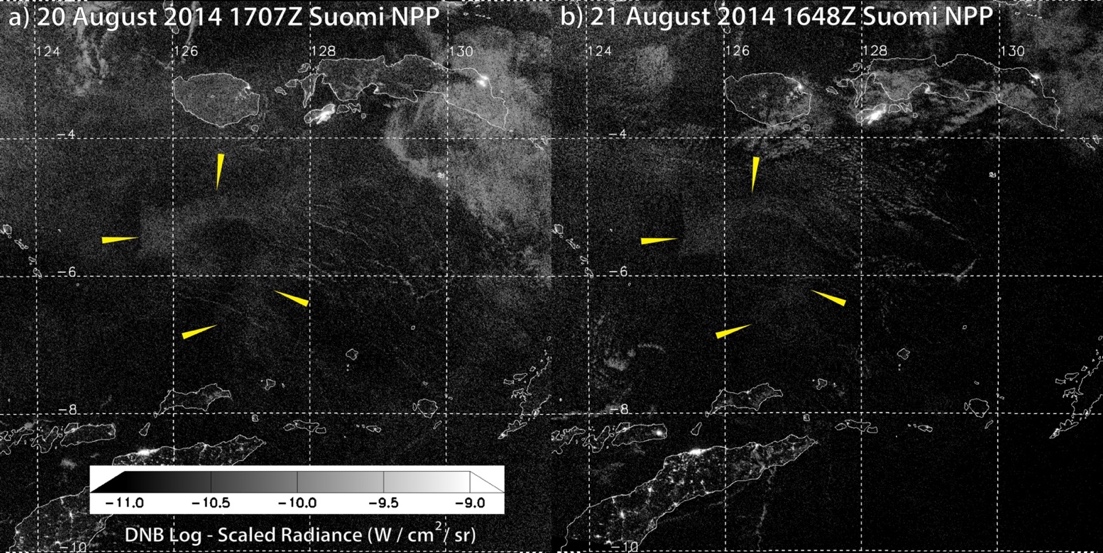
**

*Figure S2.2.1. A 2-night sequence of Day/Night Band log_10_-scaled (W cm^-2^ sr^-1^) radiance imagery showing a persistent comma-shaped weakly-emitting luminous feature (denoted by yellow pointers) in the central Banda Sea. The feature did not correlate with the cloud field and drifted slowly eastward over several nights.*


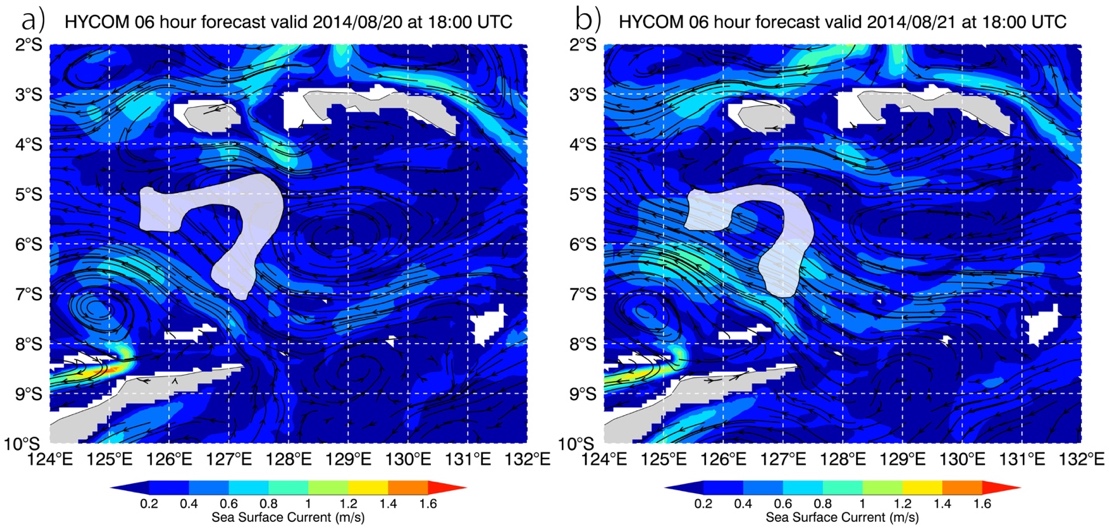


*Figure S2.2.2. HYCOM sea surface currents analysis valid at 1800 UTC on 20 (a) and 21 (b) August 2014. The approximate location of the luminous body, based on DNB observations, is shown as a shaded semitransparent object.*

Figure S2.2.2 shows that on 20-21 August the luminous body was embedded in a weak clockwise circulation that could explain its initially-observed curvature. To its south, and intersecting the southern portions of the body, was a ~200 km wide stream of southeasterly currents at ~0.5 m/s, which shifted the body west/northwest on 20 August. The currents weakened to ~0.2 m/s and became more zonal (easterly) by 23 August, and the DNB tracked the western-most portion of the luminous body westward until 24 August, after which it was no longer detectable (possibly due to its weak intensity and increased cloud cover, or else to the physical ending of the event).

**
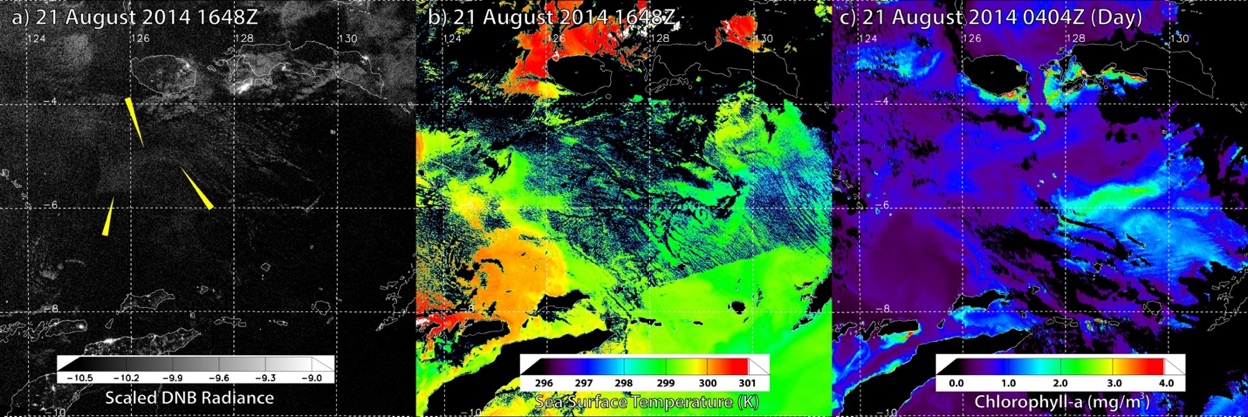
**

*Figure S2.2.3. Comparison of DNB log_10_-scaled (W cm^-2^ sr^-1^) radiance imagery (a) with retrievals of SST (b) and Chla (c; daytime) for the 2014 Banda Sea on 21 August. Chla retrievals show a comma-shaped structure with values of ~0.75 mg/m^3^ that matches approximately the weakly emitting yet persistent luminous body.*

Figure S2.2.3 shows one representative night of this sequence. The luminous body was embedded in weak westward flow, within SST of ~299 K. Its western boundary aligned with a very weak SST frontal gradient of ~0.5 K, with warmer waters to its west. The comma-shaped luminous body itself correlated positively with the structure of slightly elevated Chla with values of ~0.75 mg/m^3^ surrounded by values < 0.5 mg/m^3^.

This case occurred during the same mid-August timeframe as two other Banda Sea cases (2015 and 2019) and the Java case documented in this Supplementary Discussion. Unlike these other cases, this 2014 case corresponded to a negative phase of the Indian Ocean Dipole (IOD) and the associated Dipole Mode Index (DMI = -0.260), meaning that enhanced large-scale upwelling forces may not have been a factor. The underlying mechanisms of the Maritime Continent milky seas are not yet well understood, and warrant additional study.

# 3. Somali Sea, 15-28 January 2015

This 2015 Somali Sea event appeared in the Suomi NPP DNB imagery on the first available moonless night of the lunar cycle. It developed in a similar timeframe and location as the January 2018 event discussed in the main paper, during the northeast (Winter) monsoon. Its temporal continuity and observed rate of evolution on nights to follow suggests that it was established prior to its first detection on 15 January. The case is broken down into two sequential phases—a coastal event (Phase 1) followed by a subsequent offshore development (Phase 2).

## 3.1 Phase 1: 15-20 January 2015

On the night of 15 January, DNB imagery revealed a luminous body that did not correlate with the clouds. It initially appeared as an elongated (~750 km) filament, running roughly parallel to the Somali coastline. DNB radiance values (e.g., Figure S2.3.1) were at least 10 times higher than surrounding clear-sky ocean, per the detection criteria outlined in *Methods*. Its northern end, located offshore of southern Somalia near (2.0° N, 51.0° E), had diffuse boundaries and was ~100 km wide. Following the luminous filament southwestward toward the Somali coast, it narrowed to a width of ~20-25 km. It then paralleled the Somali coastline, maintaining a distance ~50 km offshore, from (0°, 44° E) to (2.5° S, 41.7° E). At its southernmost point, offshore of the Kenyan Lamu Archipelago, the filament curved counter-clockwise before fading near (4.0° S, 42.0° E).

*
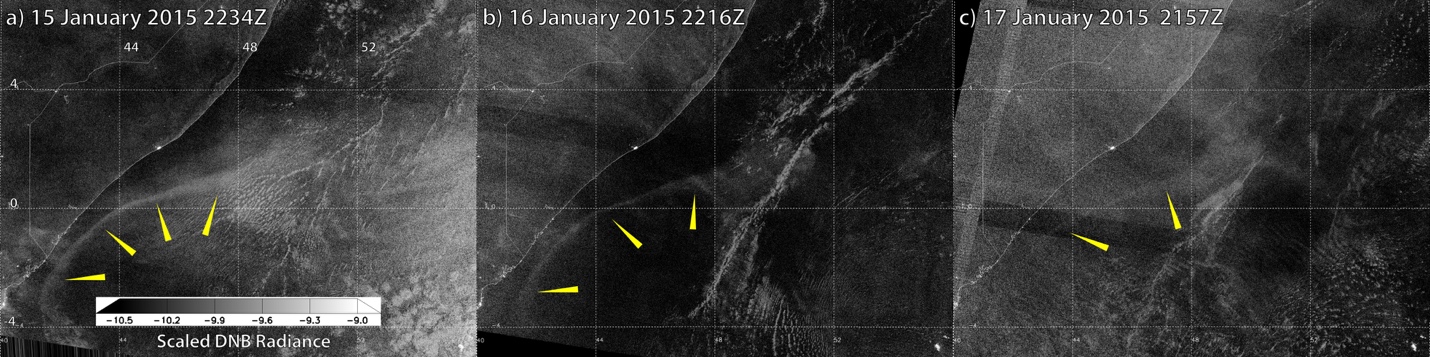
*

*Figure S2.3.1. A 3-night sequence of Day/Night Band log_10_-scaled (W cm^-2^ sr^-1^) radiance imagery for Phase 1 of the 2015 Somali Sea event, showing a persistent and slowly-evolving luminous body (denoted by yellow pointers) offshore of Somalia that did not match to the analyzed meteorological cloud field.*

The luminous filament drifted slowly offshore (i.e., eastward) over the following two nights, fading from DNB view after the 17 January overpass. However, on 25-27 January the filament was once again detectable in the same general region as before, as it fell into a more favorable part of the DNB swath (near sensor nadir). Thus the Phase 1 and Phase 2 luminous bodies were in fact concurrent.

HYCOM sea surface currents data during Phase 1 (Figure S2.3.2) revealed strong surface flow (reaching ~2 m/s) moving southwestward along the Somali coast, characteristic of the northeast (Winter) Monsoon. A counterclockwise eddy at its southern extent, offshore of the Somali Kenyan border and centered near (2.5° S, 44° E), matched generally with the DNB-observed luminous body in this area. The main area of the luminous body was located in a weak-current region between the strong coastal current and a counter-clockwise rotating eddy centered near (4° S, 50° E). Over the period of 15-17 January, the coastal currents zone broadened, consistent with a gradual offshore drift of the DNB-observed luminous body over that same period.


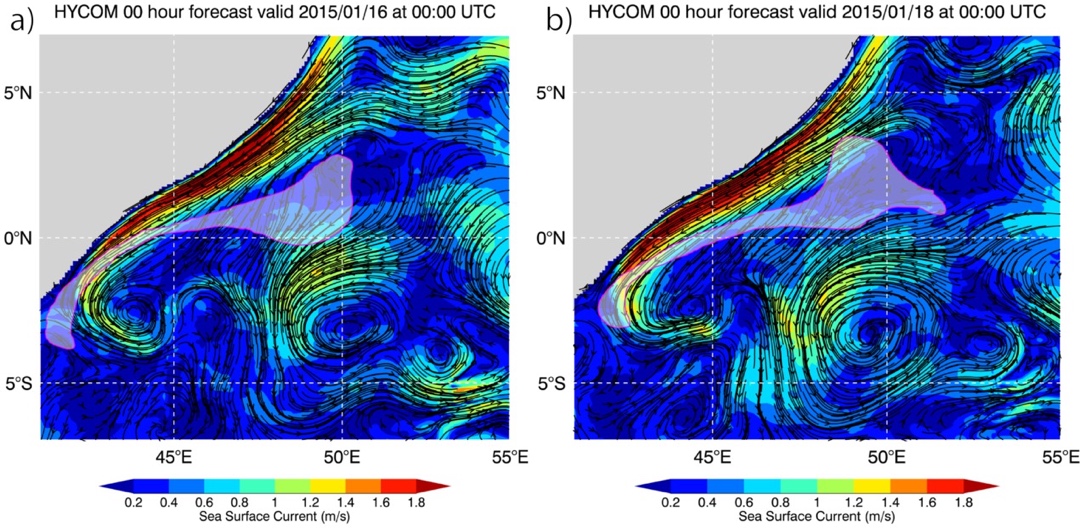


*Figure S2.3.2. HYCOM sea surface currents analysis valid at 0000 UTC on 16 (a) and 18 (b) January 2015, for comparison against Fig. S2.3.1. Approximate location of luminous body shown as shaded object. Northeasterly flow along the Somali/Kenyan coast corresponds to the northeast monsoon. Offshore curvature of the bright feature in Fig. S2.3.1 corresponds approximately with the counterclockwise eddy centered near (2.5° S, 44° E).*

VIIRS retrievals of SST and Chla revealed that the luminous body was aligned with the northwestern boundary of a tongue of relatively cooler waters (297-298 K, compared to surrounding waters that were 1-2 K warmer). Figure S2.3.3 shows SST corresponding to the DNB imagery on 16 January 2015, and Chla data roughly 36 hr later, when daytime imagery offered a cloud-free view of the region. These cooler waters corresponded to elevated values of Chla (1-2 mg/m^3^, compared to surrounding Chla values of ~0.5 mg/m^3^). While these Chla data were ~36 hours removed from the DNB image time, the HYCOM currents exhibited only small variation in the main circulation features over this period, such that these Chla values were considered representative of the luminous body’s environment roughly 37 hr prior.


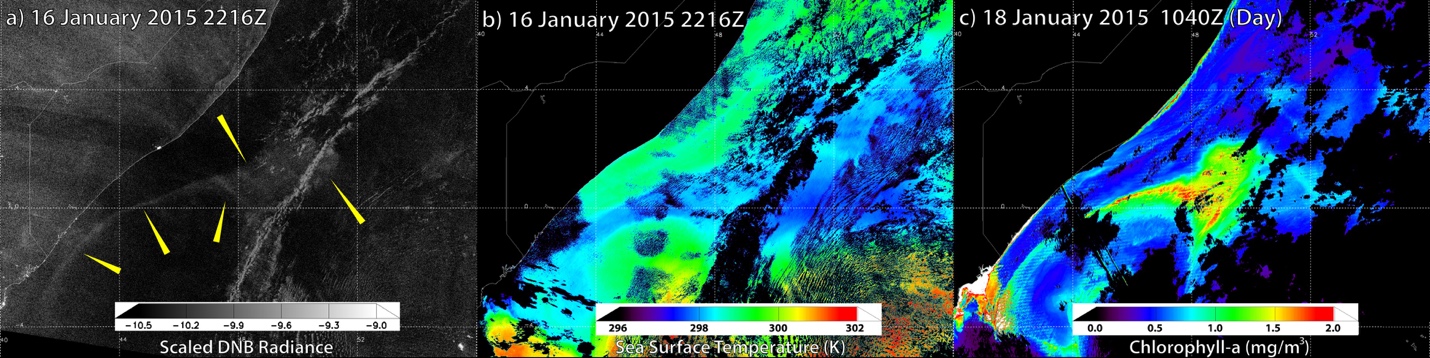


*Figure S2.3.3. Comparison of DNB log_10_-scaled (W cm^-2^ sr^-1^) radiance imagery (a) with retrievals of SST (b) and Chla (c; daytime) for the 2015 Somali Sea on 16 January. Chla retrievals were taken from the nearest-available clear-sky day (18 January) showing an arc of elevated values that resembled both the SST field and the luminous body.*

## 3.2 Phase 2: 21-26 January 2015

Phase 2 of the 2015 Somali Sea milky sea event began on 21 January, when a “*crescent*”-shaped luminous body was apparent in the DNB nighttime imagery centered near (0.0°, 48.0° E). Its initial identification amidst the widespread cloud and airglow structures on this first night was challenging, but the persistence and slow drift of the *crescent* as a contiguous body over ensuing nights enabled decoupling from those ephemeral contaminants, per *Methods*. Figure S2.3.4 shows the *crescent* as observed over six nights from 21-27 January (24 January was skipped due to poor DNB scan-edge coverage). The night of 22 January offered the highest quality DNB imagery, when the *crescent’s* southwestern point was located near (2.0° S, 48.0° E) and its broader northeastern end was located near (2.0° N, 52.0° W)—a linear span of over 600 km.


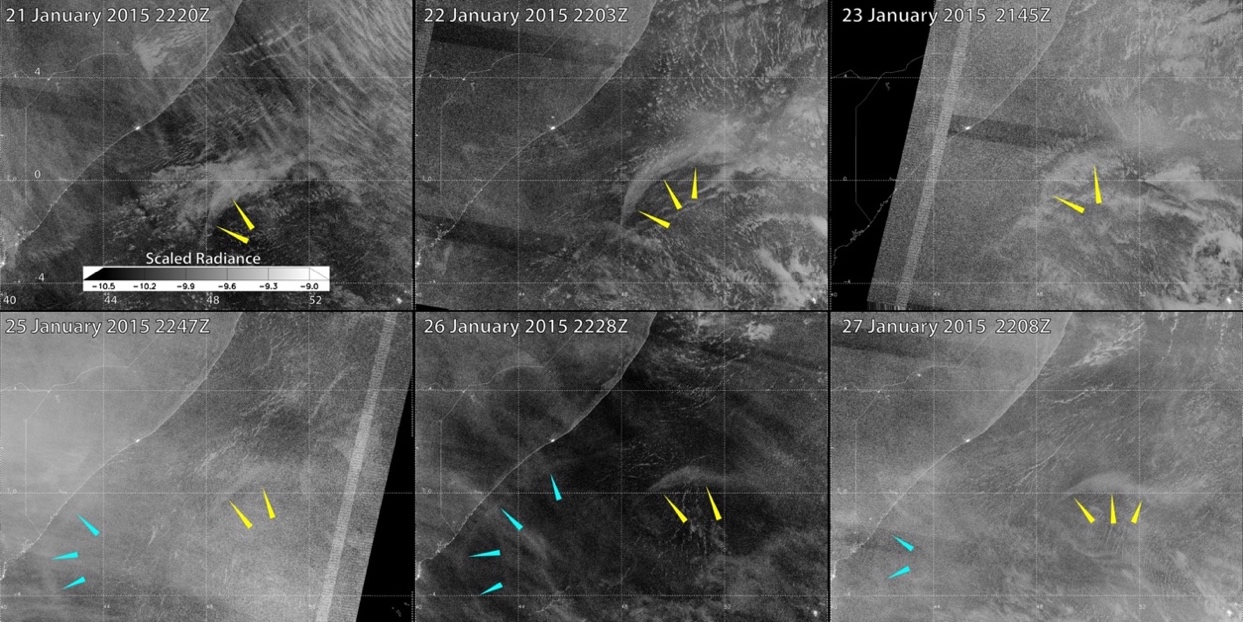


*Figure S2.3.4. A 6-night sequence of Day/Night Band log_10_-scaled (W cm^-2^ sr^-1^) radiance imagery from the 2015 Somali Sea milky sea, showing a persistent “crescent”-shaped luminous body offshore of Somalia (yellow pointers) which did not correspond to the meteorological cloud field. A lingering portion of the Phase 1 filamentary structure is also noted (blue pointers).*


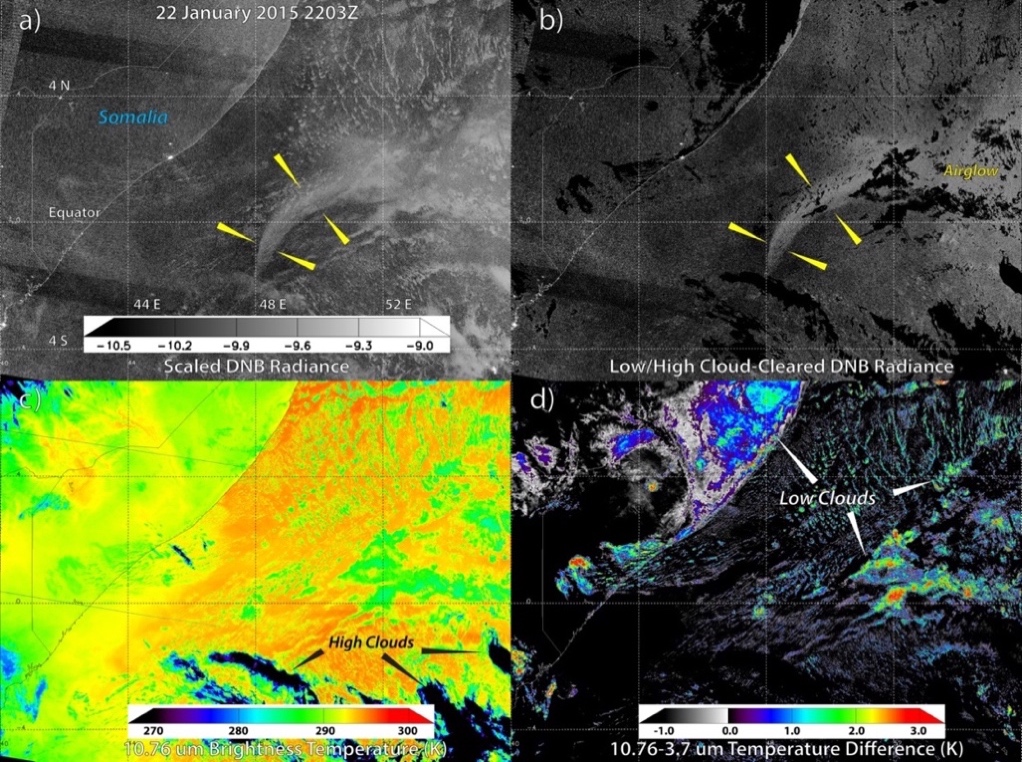


*Figure S2.3.5. a) Nighttime DNB log_10_-scaled (W cm^-2^ sr^-1^) radiance imagery from 22 January 2015, 2203 UTC, b) residual DNB imagery from (a) after masking of infrared-detected clouds via c) thermal infrared imagery (high clouds in blue/purple), and d) 10.7 - 3.7 μm VIIRS brightness temperature difference (with positive/colored values corresponding to low-clouds).*

Figure S2.3.5 shows how the DNB-observed luminous *crescent* on 22 January was distinguished from the meteorological cloud field. In many cases documented in this study, a signature of the luminous bodies was still discernible beneath cloud cover, due to visible light scattering through the cloud of low to moderate optical thickness. The area of the luminous *crescent* was ~60,000 km^2^, approximately the same size as the state of West Virginia. The *crescent* drifted north over 21-25 January, and by the end of this period its southern point had drifted northeast to (1.0° S, 49.0° N). Between 25-27 January, it underwent slow clockwise rotation and southward drift.

HYCOM analyses showed moderate (0.8 - 1.0 m/s) easterly equatorial currents on 20-22 January. This flow transitioned to weaker (0.5 - 0.8 m/s) northeasterly currents by 24-27 January. Unlike all other cases analyzed in this study, the *crescent* feature’s eastward drift in this case contradicted the HYCOM currents. However, additional analysis of SST and Chla suggest that surface flow in the vicinity of the bright feature was indeed tracking eastward—pointing to either an error in this particular HYCOM analysis (or small spatial shift—noting that a region of doldrums occurred immediately to the north) or our own misinterpretation of the effect these currents would have on the propagation of the luminous body for this case.

Distributions of SST and Chla during Phase 2 (e.g., Figure S2.3.7) show a significant and persistent algal bloom originating near the Somali/Kenyan coast and drifting east/northeast, confined to a relatively cool water (SST < 298 K) region. Daily retrievals of these ocean surface parameters showed the bloom drifting toward the east/northeast over the Phase 2 period, suggesting that the luminous body (which tracked with these parameters) moved with prevailing currents for this area, despite what was inferred from the HYCOM currents field. Here, the bright feature appears along the southern and eastern flanks of the Chla bloom. As with the Phase 1 feature, the Phase 2 luminous body did not correspond to the coolest SST and highest values of Chla, but instead resided along the peripheries and was associated with relatively moderate levels of SST (~298 K) and Chla (~0.5-1 mg/m^3^).


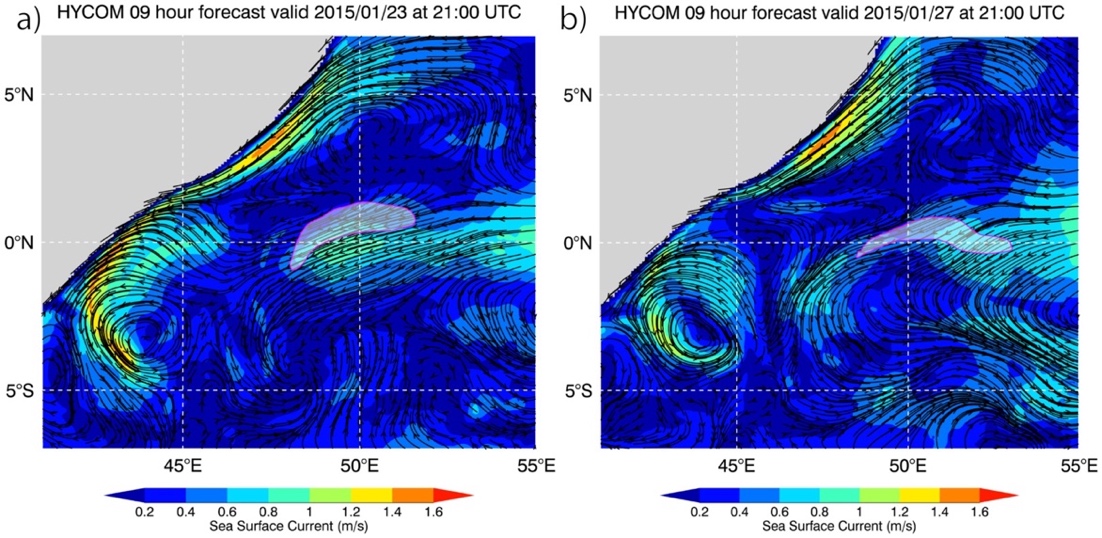


*Figure S2.3.6. HYCOM sea surface currents analysis valid at 0000 UTC on 23 (a) and 27 (b) January 2015. Approximate location of luminous body shown as shaded object.*


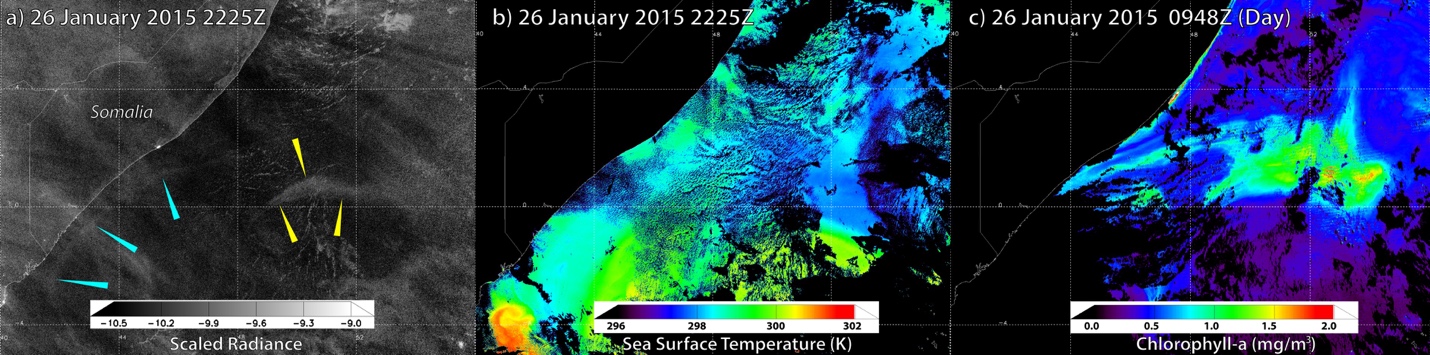


*Figure S2.3.7. Comparison of a) DNB log_10_-scaled (W cm^-2^ sr^-1^) radiances with matching retrievals of b) SST and c) Chla (daytime) for the 2015 Somali Sea event on 26 January. The Phase 2 crescent in DNB imagery (yellow pointers) aligned with the southern perimeter of relatively cool SST and elevated Chla. Blue pointers in DNB imagery show the lingering Phase 1 feature.*

Over the nights of 27-28 January the eastern side of the *crescent* connected with a faint “*S*”-shaped luminous body to its east/northeast, centered on the 53° E meridian and extending from 0.5° S to 4.0° N. HYCOM surface currents analyses suggest that the center of the “*S*” occurred along a bifurcation of streamlines in the westward-flowing currents at (2.5° N, 53.0° E), with the northern and southern portions flowing in opposite directions. The “*S*” occurred along the boundary of cool SST and elevated Chla, with values similar to those noted for the *crescent*. After 28 January, moonlight ended DNB monitoring of this event, and no luminous bodies were detected in the following moon-free cycle, two weeks later.

# 4. Banda Sea, 12-18 August 2015

This case occurred in the same mid-August timeframe as the other two Banda Sea cases (2014 and 2019) reported on in this study. At this time, the IOD was in a pronounced positive phase (DMI = 0.680), conducive to stronger upwelling over the region and associated higher primary production. A challenge encountered here, common to all the Banda Sea cases, was widespread cloud cover. These clouds reflect the downwelling atmospheric airglow and starlight, producing features that can obscure or confuse the interpretation of any underlying luminous features also present in DNB imagery. Thus, positive detection of luminous bodies require persistence over multiple nights to distinguish them with higher confidence, per *Methods*. If future low-light sensors offer multispectral capability at the sensitivity levels required for milky sea detection, then color information may assist in this discrimination.

*
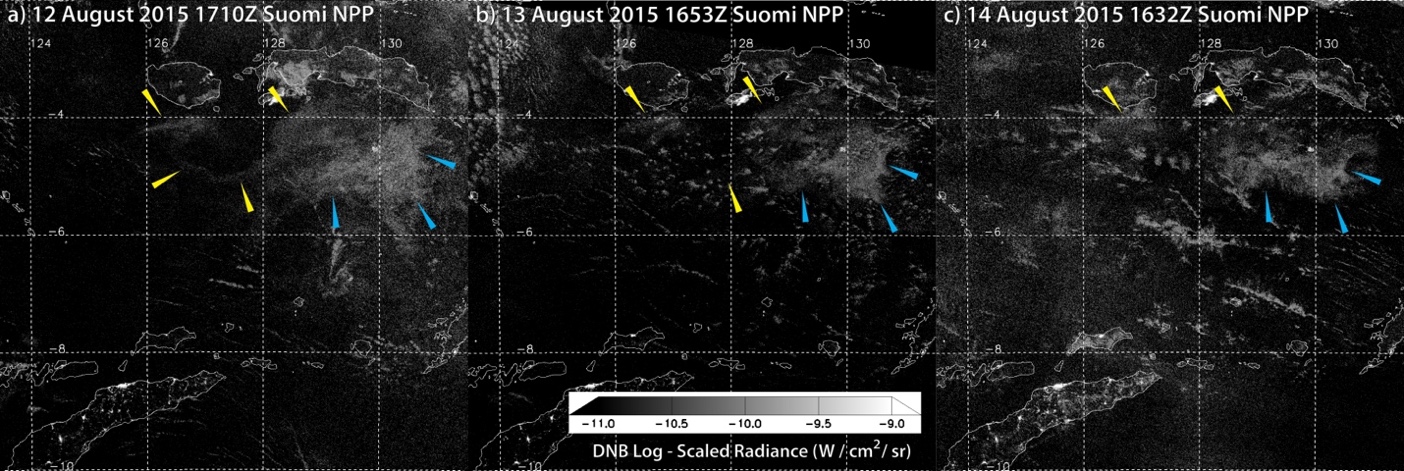
*

*Figure S2.4.1. Day/Night Band observations of weakly emitting yet persistent luminous features, showing the inverted “Ω”-shape (yellow pointers) and a broader luminous body to the east (blue pointers) for 12-14 August dates of the 2015 Banda Sea case. These features did not correlate with the cloud field.*

Figure S2.4.1 shows a three-night sequence of DNB observations with various luminous bodies noted. These bodies persisted and slowly drifted over the course of the full 7-night sequence. Two primary objects were identified—one appearing as an inverted “*Ω*,” and a larger amorphous area near (4.5° S, 129° E). The larger area was commingled with variable cloudiness during this sequence, but as in other cases the upwelling light from the luminous surface diffused through the conservatively scattering clouds, permitting its detection. The location of the event is consistent with past reports of milky seas in the Banda Sea encountered (Supplementary Discussion 1).

*
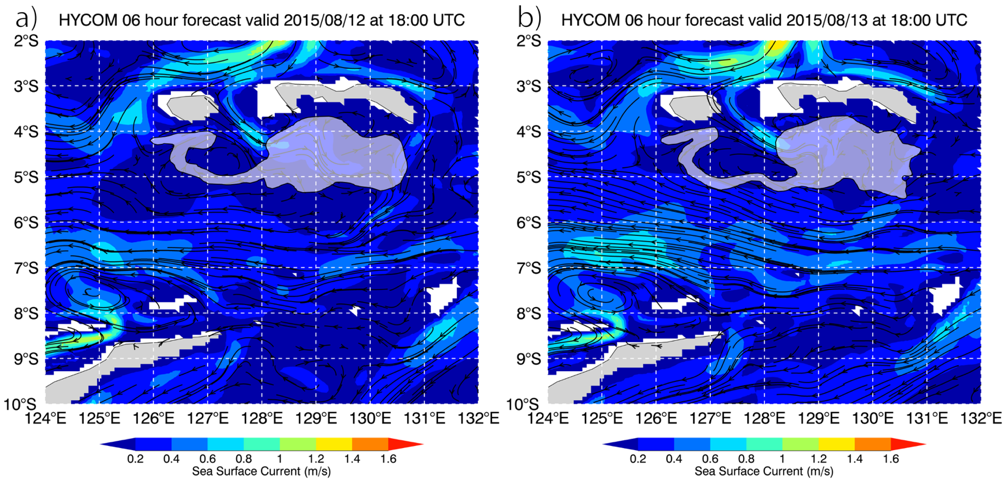
*

*Figure S2.4.2. HYCOM sea surface currents analysis valid at 1800 UTC on 12 (a) and 13 (b) August 2015. Approximate location of luminous body shown as shaded object.*

HYCOM data (e.g., Figure S2.4.2) showed that the luminous bodies formed in a large area of weak currents. The inverted “Ω” was situated within a weak clockwise-rotating eddy south of Buru, Maluku. The larger luminous area to the east exhibited a clockwise rotation which could be explained in part by northeasterly currents passing by Seram, Maluku.

*
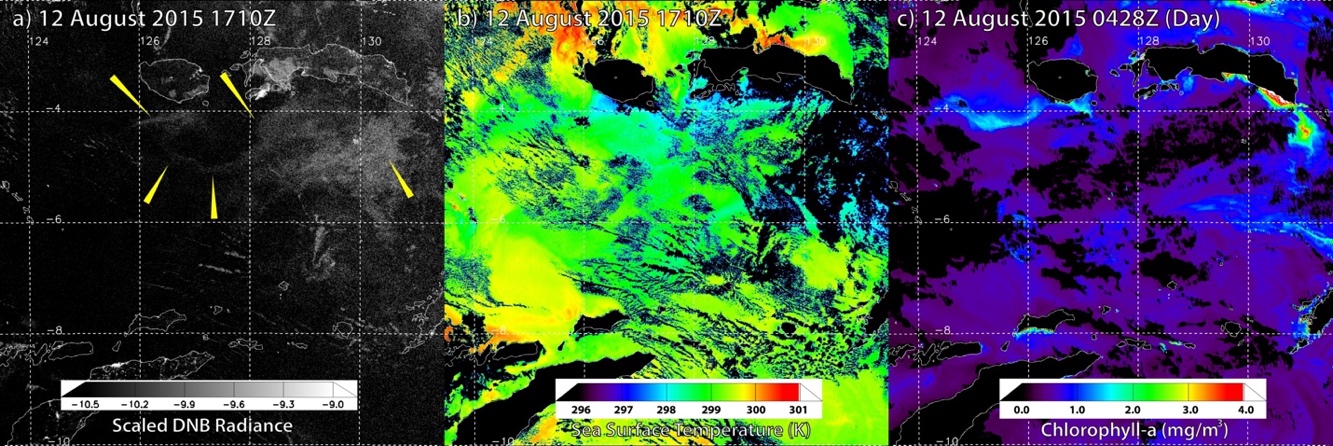
*

*Figure S2.4.3. Comparison of DNB imagery (a) with retrievals of SST (b) and Chla (c; daytime) for the 2015 Banda Sea on 12 August. Chla retrievals showed elevated (> 2 mg/m^3^) values in cooler (~297.5K) waters to the north of the inverted Ω structure and to the east of the large eastern luminous body, while the luminous bodies themselves had values of ~0.5 mg/m^3^ and were embedded in SST of ~299K.*

Satellite retrievals showed cooler waters (SST ~297-298K) and elevated Chla (~1.5 mg/m^3^) to the north of the inverted “Ω,” on the southern shore of the island of Buru, Maluku (Figure S2.4.3). The larger eastern body bordered a cooler area of water moving in from the east. Both luminous bodies were embedded in SST of ~299K, and while the Chla values corresponding to the bodies were ~0.5 mg/m^3^, each body flanked much higher values of Chla, with values exceeding 2 mg/m^3^ immediately to the north of the inverted *“*Ω” and immediately to the east of the larger eastern body. These associations were a consistent theme of the cases encountered in this study.

# 5. Socotra (Guardafui Channel), 7-20 September 2015

Unique among the cases documented in this study, this event occurred in the Guardafui Channel, located between the Horn of Africa and the island of Socotra. Although historical reports of milky seas are dominated by the southwest (Summer) monsoonal mode, this case was among only two events (the other being the July-August 2013 Socotra case) detected by the DNB over the 2012-2021 survey period. Our discussion of this case is partitioned into two phases—a weaker luminosity initial phase and a stronger secondary phase.

## 5.1 Phase 1: 7-11 September 2015

The Socotra event was first detected on 7 September, 2222 UTC, when the DNB observed near (10.0° N, 53.0° E) a horizontally-oriented luminous body of dimension ~10 x 50 km. By 8 September 2200 UTC the body had drifted north by ~10 km, and a second, triangular-shaped (~25 km on a side) body appeared ~30 km to the north. Infrared-based cloud detection tests did not identify either of these bodies. By 9 September 2143 UTC both bodies had drifted northward together for an additional ~10 km, but then poor-quality DNB imagery on the following two nights (9-10 September) precluded their tracking. By 11 September, the two bodies appeared to have merged into a diffuse “*comma”*-shaped luminous body whose brightest portion was centered on (11.0° N, 52.5° E).

HYCOM sea surface currents indicated strong northeastward-flowing coastal currents immediately south of the Phase 1 observed features. This flow, with speeds up to 2.6 m/s, curved offshore of Somalia to the east near 10° N. As it headed offshore, the flow forked near (11.5° N, 53.0° E). The western fork moved north/northwest and passed through the gap between the Horn of Africa and Socotra. This surface currents pattern could explain the observed northward drift of the two luminous bodies.

SST and Chla retrievals during Phase 1 show that the luminous bodies corresponded to relatively cool SSTs (298 K) and moderate levels of Chla (~1 mg/m^3^) near the coast, properties consistent with other cases examined in this study. Significantly cooler waters and higher values of Chla were present closer to the coastline—the result of strong Eckman-related upwelling tied to the northeastward currents of the Summer Monsoon.

## 5.2 Phase 2: 12-20 September

Phase 2 of the Socotra case began on 12 September, when at 2228 UTC in the evening the DNB detected a faint oblate luminous body under clear sky conditions, centered on (11.0° N, 51.8° E). DNB imagery on the following night of 13 September revealed a better view of same body, which now appeared more as a “*teardrop*”-shape, centered at 52.0° E, having a narrow northern tip at (11.5° N, 51.8° E) and a oblate southern end at (10.0° N, 51.8° E). Figure S2.5.1 shows a six-night sequence of Phase 2. Near its center, the *teardrop* was ~100 km wide, and its total area was ~15,000 km^2^. It remained intact and in this form through the night of 16 September, drifting ~30 km to the east of its initial position. On 17 September, despite cirrus overcast skies, the feature was again prominent (seen via forward-scattering of upwelling light). By this time, its shape had changed into a vertically-oriented ellipse. The ellipse had counter-clockwise hook-shaped outcroppings on its northwest edge, as if material was being sheared off. The luminous body continued in a slow east/northeast drift, reaching (11.0° N, 52.5° E) by 18 September, when a similar hooked outcropping appeared on its southeastern edge.

A near-nadir overpass on 19 September at 2156 UTC (lower-right panel of Fig. S2.5.1) offered a clear DNB view of the luminous body. At this time, it was centered at (11.0° N, 52.5° E) and had a sharply defined and notably brighter eastern flank. Both its northwestern and southeastern flanks maintained hook-shaped outcroppings, as seen on the previous two nights. DNB imagery on the next night (20 September 2138 UTC) was contaminated by cloud cover that complicated feature identification, and moonlight entered the region on subsequent nights. There were no signs of the Phase 2 luminous body when moon-free conditions returned two weeks later.

HYCOM sea surface currents (Figure S2.5.2) showed a doldrum of slowly counterclockwise rotating waters between the Somali coast and the western fork of the aforementioned northbound current. It was within these doldrums that the Phase 2 luminous body first appeared. By 13 September, a weak east/northeastward drift had developed within these doldrums, consistent with the observed drift direction of the luminous body. As the body approached the stronger flow of the northbound-split current, increasing horizontal gradients in current on both its northern and southern peripheries could explain the hook-shaped outcroppings that began to appear on 18-19 September, in terms of a shearing-off of material.


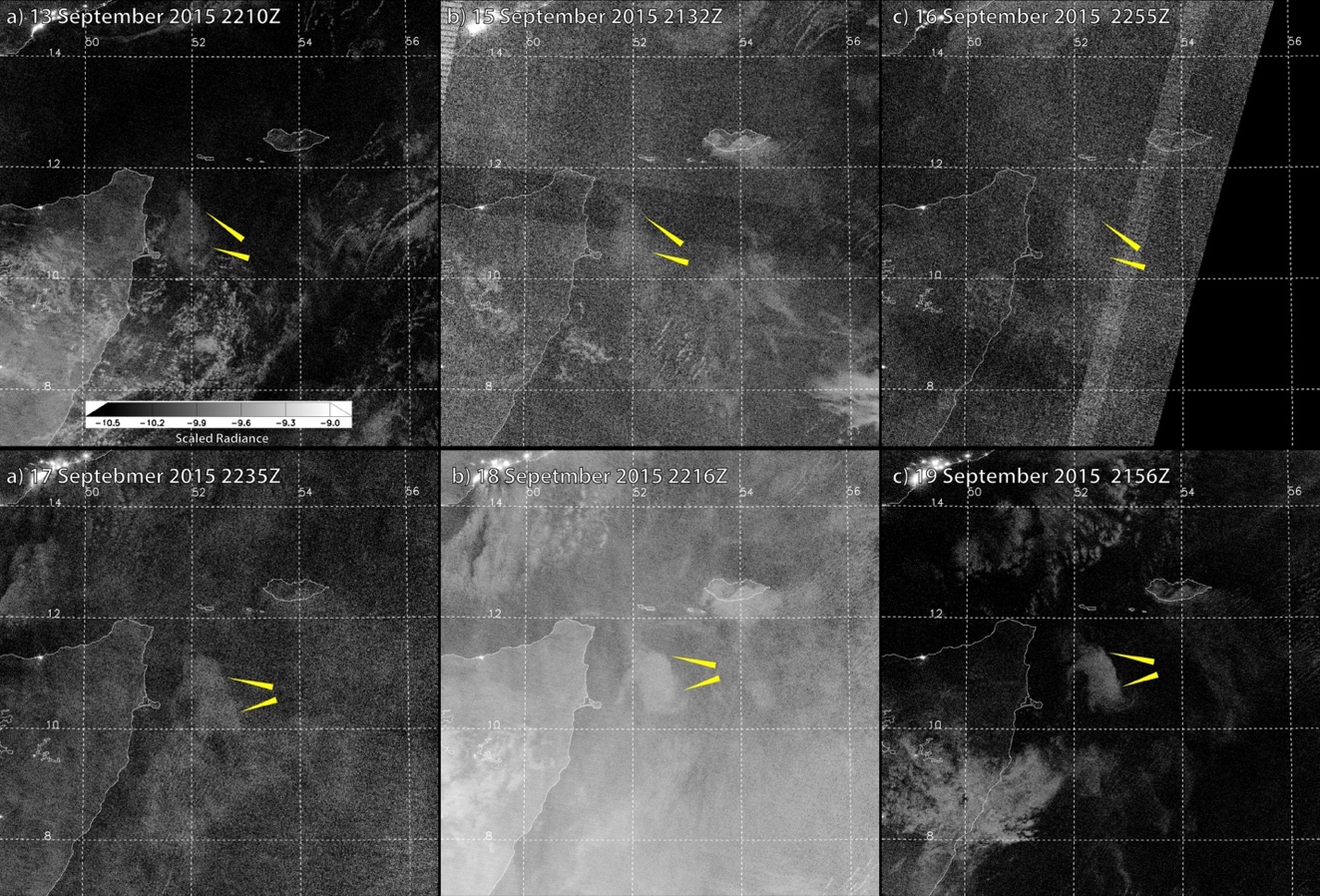


*Figure S2.5.1. A 6-night sequence of Day/Night Band imagery for the September 2015 Socotra milky sea, showing a luminous body off the Horn of Africa (yellow pointers) which did not correspond to the meteorological cloud field.*

SST and Chla retrievals showed a region of cooler waters (~3-4 K cooler than the surrounding waters) in the HYCOM-analyzed region of doldrums where the luminous body resided. Elevated values of Chla (exceeding 2 mg/m^3^) existed along the coastline but were not significant (~0.5-1 mg/m^3^) in the central portion of these doldrums. The specific date of 18 September is shown in Figure S2.5.3. Here, as in other cases, the doldrums were characterized by SST values near 298 K and low to moderate levels of Chla near 0.5 mg/m^3^. Notably, the SST revealed a structure reminiscent of the DNB luminous body’s boundary. A similar alignment was observed between the SST and the body’s boundaries on adjacent nights as well, suggesting a decoupling of these waters from the surrounding region and supporting the *natural flask* hypothesis as postulated in the main paper.


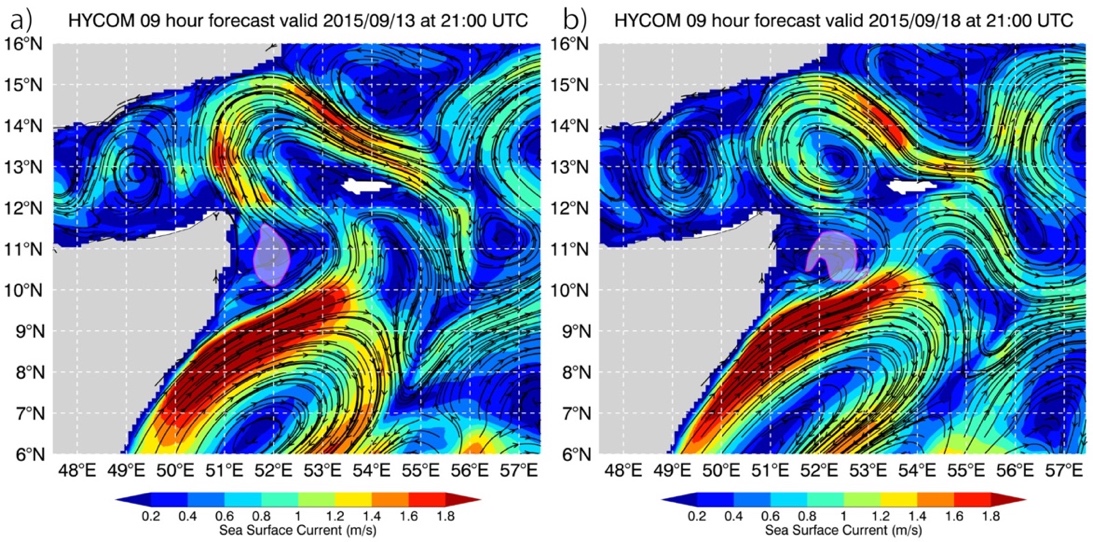


Figure S2.5.2 *HYCOM sea surface currents analysis valid at 2100 UTC on 13 (a) and 18 (b) September 2015, for comparison against the feature identified in Figure S2.5.1. The approximate locations of the luminous body on these two nights are shown as shaded objects.*


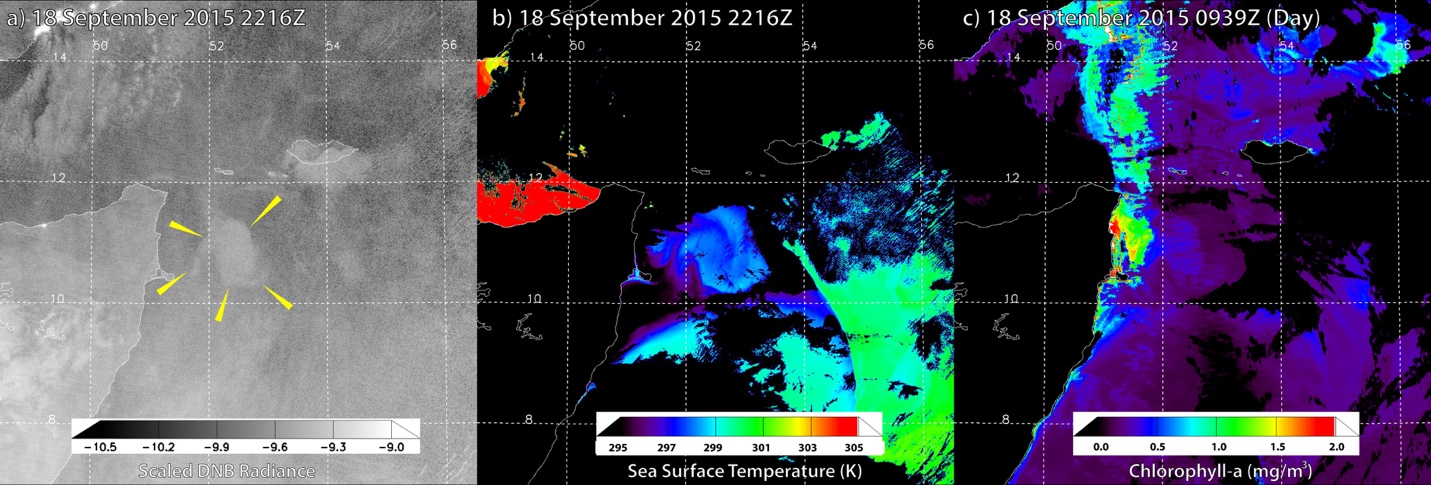


*Figure S2.5.3. Comparison of DNB imagery (a) with matching retrievals of SST (b) and Chla (c; daytime) for the 2015 Socotra event on 18 September. The luminous body in DNB imagery (denoted by yellow pointers) appears to share some structural similarities to the SST field, and correlates with a weakly-elevated (~0.5 mg/m^3^) structure of Chla, while much larger values of Chla occur along the immediate coast.*

HYCOM surface current velocity data were used to compute deformation parameters, useful in comparing against the observed morphology and evolution of the luminous bodies. These fields, derived from zonal (U) and meridional (V) horizontal current velocity components:

$U=\left( U,V \right)$, (1)

include the normal, shear, and total strain (S_n_, S_s_, and S_t_, respectively):

$S_{n}=\frac{\partial U}{\partial x}-\frac{\partial V}{\partial y}$ ,

$S_{s}=\frac{\partial V}{\partial x}+\frac{\partial U}{\partial y}$ ,

$S_{t}=\sqrt{\left( S_{n}^{2}+S_{s}^{2} \right)}$ , (2)

the relative vorticity:

$\zeta= \frac{\partial V}{\partial x}-\frac{\partial U}{\partial y}$ (3)

the Okubo-Weiss parameter:

$\alpha^{2}=\left( S_{n}^{2}+S_{s}^{2}+\zeta^{2} \right)/4$ (4)

and the effective Coriolis frequency (*f_eff_*), derived from the Coriolis parameter

$f=2\Omega\sin\varphi$ (5)

as:

$f_{\text{eff}}=\sqrt{\left( f+\zeta/2 \right)^{2}-\left( S_{n}^{2}+S_{s}^{2} \right)/4}$ , (6)

where *f* is the Coriolis parameter, Ω is the rotation rate of earth (7.2921 × 10^−5^ rad s^-1^) and ϕ is latitude.

The HYCOM deformation fields were used to examine the cause of the hook-shaped outcroppings and assess the degree to which the waters were decoupled from the surrounding flow. Figure 2.5.4 shows an example of several of the fields corresponding to the 19 September 2015, 2156Z DNB overpass (which exhibited the hook-shaped outcroppings on its northwest and southeast sides). The fields show that the luminous body resided in a calm region characterized by low strain and weak cyclonic flow, with stronger positive vorticity along the body’s perimeter. This structure supports the shedding of cyclonic (counter-clockwise) oriented filaments, as observed. The fields also confirm the isolation of these luminous waters, supporting the premise of the natural flask hypothesis.


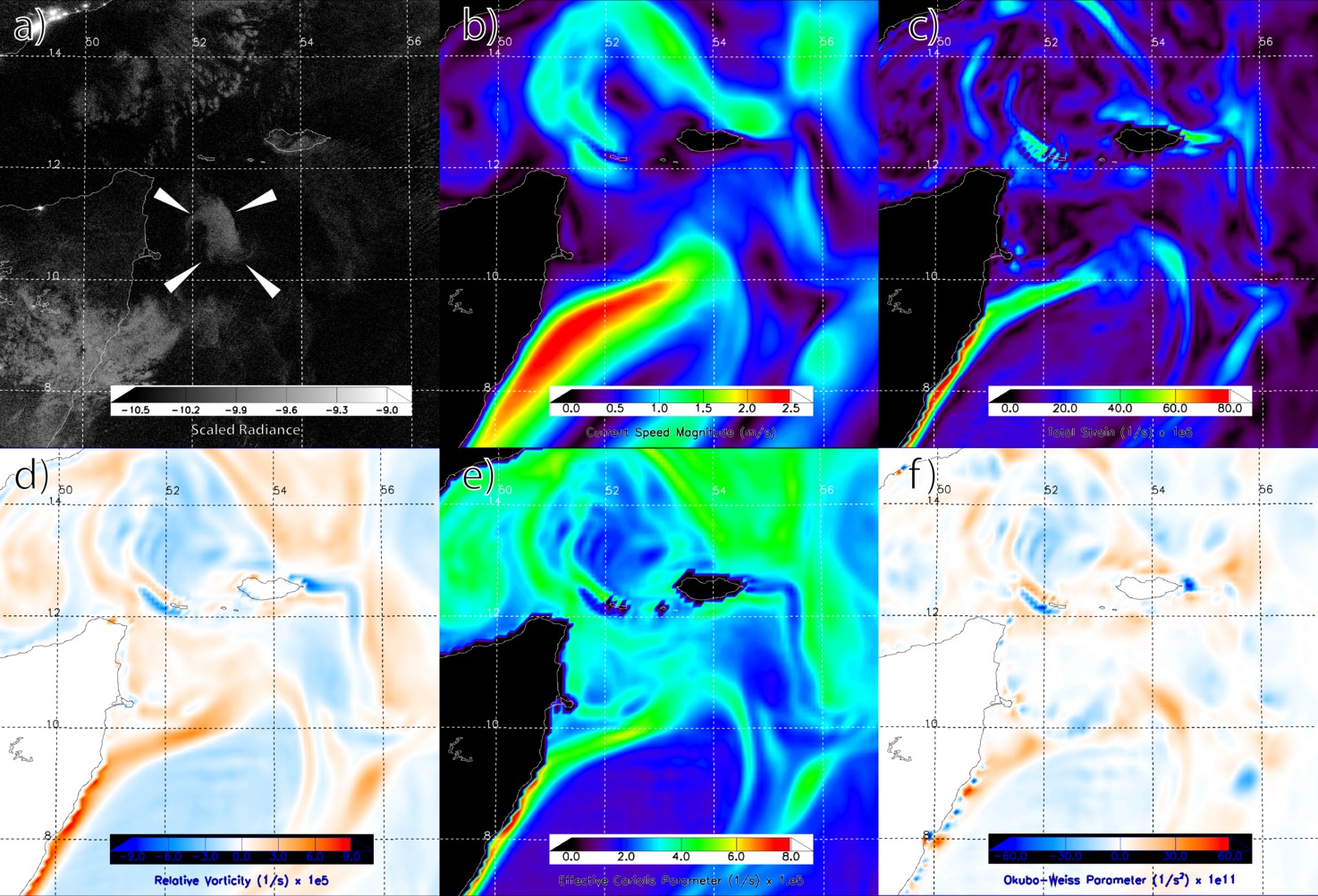


*Figure S2.5.4. VIIRS Day/Night Band imagery from 19 September 2015, 2156Z (a; white pointers denote the location of the luminous body) Socotra milky sea, along with HYCOM analyzed surface current speed (b), total strain (c), relative vorticity (d), effective Coriolis frequency (e), and Okubo-Weiss parameter (f).*

# 6. Somali Sea, 21-31 January 2017

This event bore strong similarities to the January 2015 Somali Sea milky sea case study in terms of its area of formation, timeframe, and structural details of the luminous body itself. While there were weak-signal indications of it extending further offshore, the body was most distinguishable close to the Somali coast over its duration, and thus is characterized here as a single-phase event.

Figure S2.6.1 shows four nights of DNB imagery selected from the full 11-night sequence. On 21 January a narrow ‘*wishbone*’-shaped luminous body appeared in the DNB 2205 UTC imagery. Its vertex was located near (2.0° N, 47.0° E). One of its arms extended eastward and the other to the northeast (paralleling the Somali coast), each being roughly 20 km wide and extending for ~350 km. The total area of the *wishbone* on this date was ~17,000 km^2^. Parts of this body were evident on 22 January, followed by two consecutive nights (23-24 January) of poor-quality (scan-edge) DNB imagery when it was missed. It reappeared in DNB imagery on the night of 25 January, in roughly the same location and orientation as before.


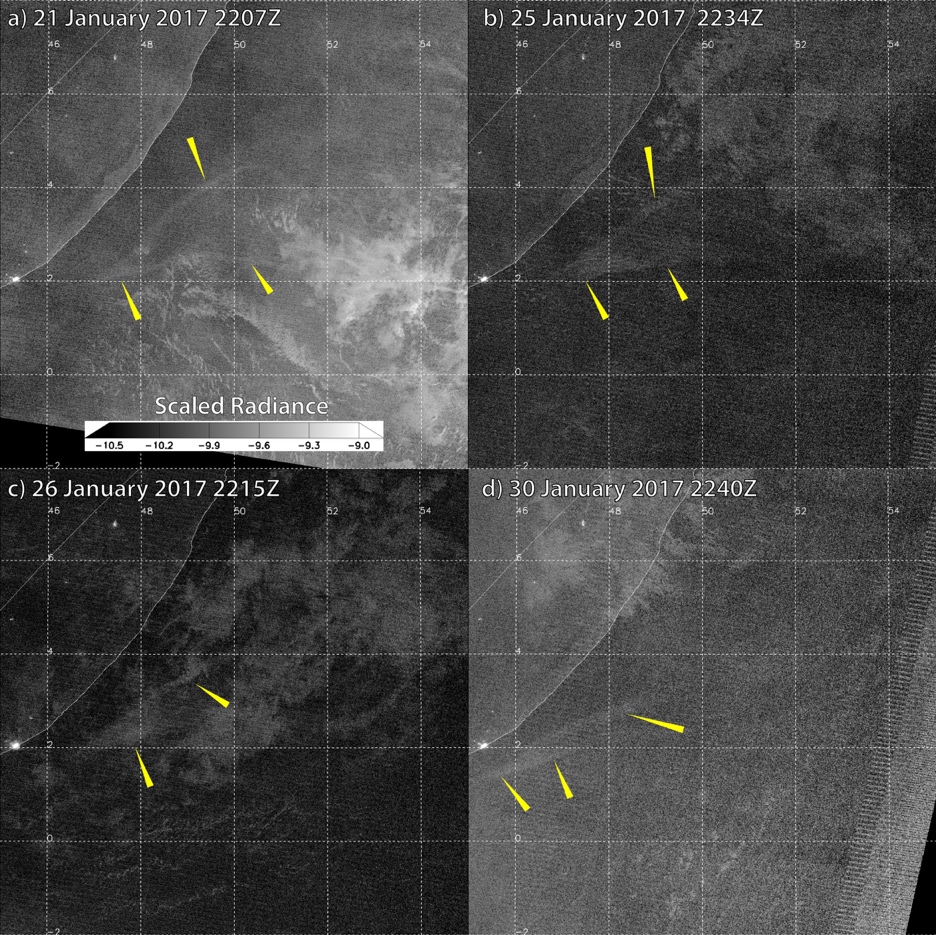


*Figure S2.6.1. Day/Night Band imagery for the January 2017 Somali Sea milky sea, showing a persistent “wishbone”-shaped luminous body offshore of southern Somalia which persisted over multiple nights and did not correspond to the meteorological cloud field.*

Over the following two nights of 26-27 January the arm of the luminous body that was closer to the Somali coast drifted slowly eastward (i.e., offshore). On the following 2 nights of 28-29 January the DNB once again suffered poor-quality (scan-edge) imagery. The luminous body reappeared in more nadir-viewing overpasses on 30-31 January. By this time, it had stretched into more of a ‘*hairpin*’ shape, its two appendages now running in parallel. Its vertex had drifted to the southwest; ~60 km south of Mogadishu, Somalia, near (1.5° N, 45.3° E).


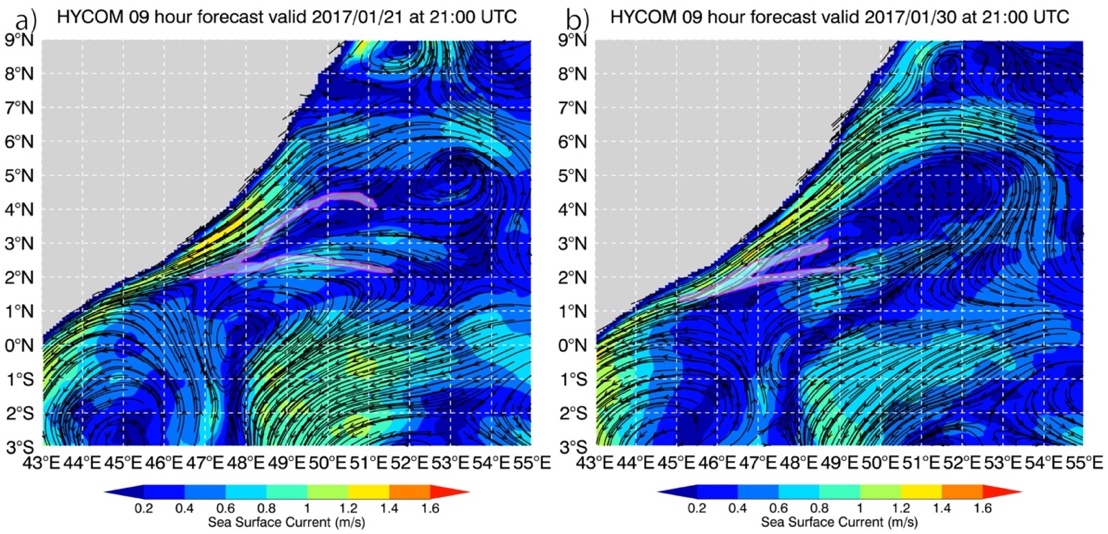


Figure S2.6.2 *HYCOM sea surface currents analysis valid at 2100 UTC on 21 (a) and 30 (b) January 2017.*

HYCOM data (e.g., Figure S2.6.2) show moderate (1.0 - 1.2 m/s) southwestward currents close to the Somali coast. A deformation zone in the currents was present near (2° N, 47° E). The luminous body was bounded by the southwestern perimeter of these features; with flow associated with the northern arm of the *wishbone* apparently moving southwest along the coast, and flow in the southern (east-west oriented) arm moving eastward. The stretching and southwestward propagation of the entire feature during its latter stages could be due to its entrainment within the stronger coastal currents. A slight shift of the counterclockwise swirl toward the Somali coast on 24-26 January correlated with the luminous body’s observed southwestward drift and stretching.

***
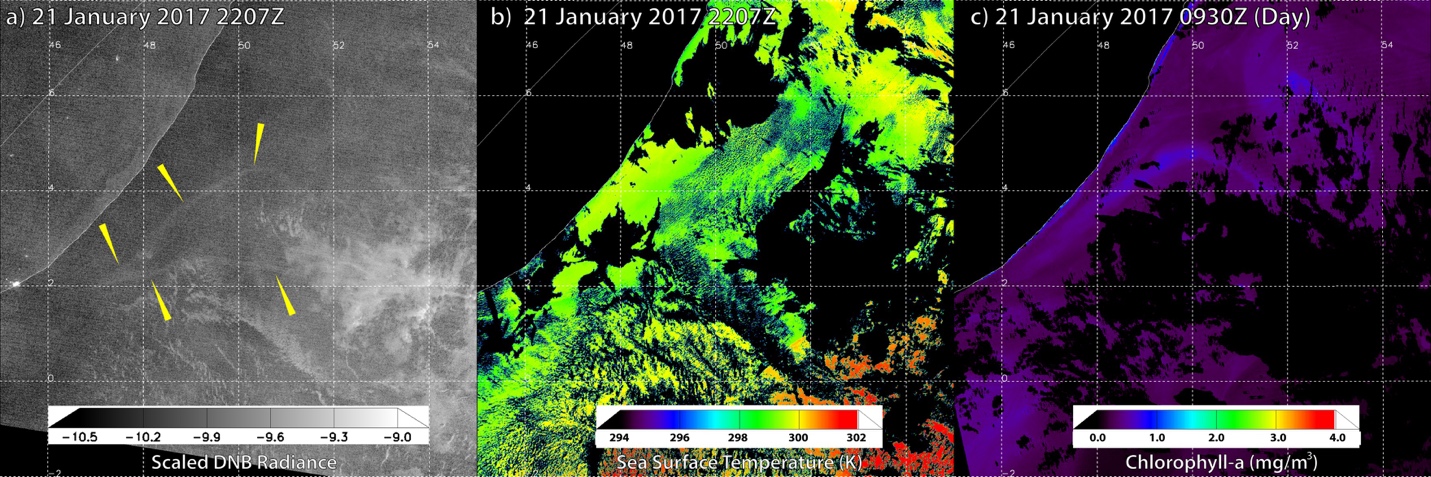
***

*Figure S2.6.3. Comparison of DNB imagery (a) with matching retrievals of SST (b) and Chla (c; daytime) for the 2017 Somalia event on 21 January. The ‘wishbone’ luminous body seen in DNB imagery yellow pointers) shared structural similarities to an area of relatively cool SST and was adjacent to slightly elevated values of Chla.*

Retrievals of SST and Chla for this case (e.g., Figure S2.6.3) show that the luminous body resided in a region of relatively cooler waters near 298 K; a value that appears to hold some degree of significance to the cases encountered in this study. While Chla levels were unremarkable, a filamentary structure in 0.6 – 0.7 mg/m^3^ range aligned with the HYCOM surface currents. The northern arm of the *wishbone* flanked the southern perimeter of this Chla structure, and as with other cases, did not align directly atop the largest Chla values.

# 7. Somali Sea, 12-23 January 2018

The 12-23 January 2018 Somali Sea case began in a similar way as the January 2015 and the January 2017 cases for this same region, suggesting a recurring pattern of formation whose details are not yet well understood. The event transpired as two phases—an initial event close to the Somali coast and a second more substantial event developing subsequently further offshore (and detailed in the main paper).

## 7.1 Phase 1: 12-19 January 2018

On 12 January, the DNB detected the first signs of a diffuse-edged luminous body near (1.9° N, 47.2° E), ~200 km offshore of Mogadishu, Somalia. On the following night, 13 January, the sky cleared over the area, revealing a ‘*wishbone*’ shaped structure (Figure S2.7.1). The structure was strikingly similar in both shape and orientation to the January 2017 Somali Sea case. It was characterized by two narrow (~15 km wide) filaments, each extending and broadening toward the northeast. The western filament was located ~150 km offshore of Somalia, and ran roughly parallel to the coast at an azimuth angle (clockwise with respect to due north) of 50° and extending northeastward for nearly 500 km. The northern extent of this coastal arm rotated clockwise through an angle of ~180° with a ~50 km radius of curvature centered at (4.0° N, 50.4° E). The eastern filament was oriented at 68° azimuth. Its northern extent merged into cloud cover on this night.

*
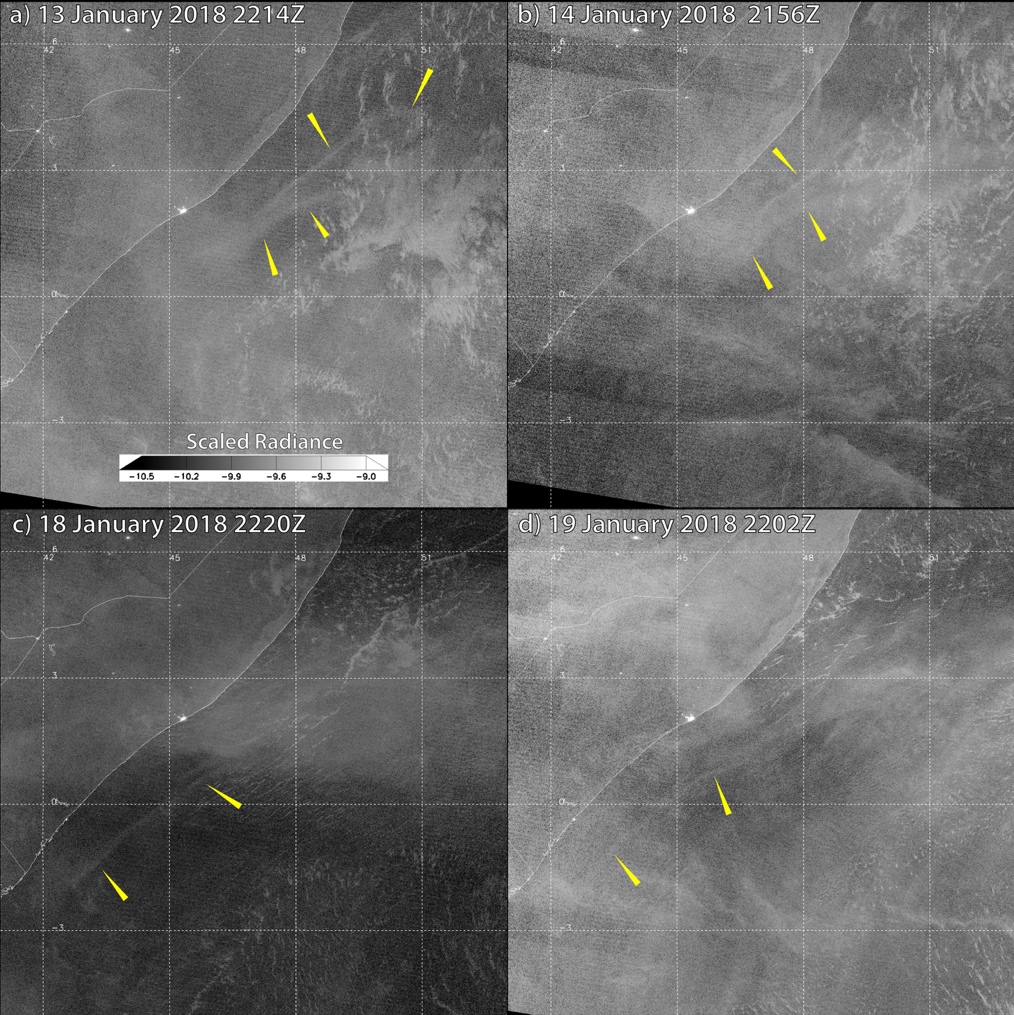
*

*Figure S2.7.1. Day/Night Band imagery for selected nights of the January 2018 Somali Sea milky sea, showing a persistent wishbone-shaped luminous body that evolved into a single, elongated filament. The luminous body did not correspond to the meteorological cloud field.*

On the nights of 14-15 January, the western filament of the *wishbone* drifted slightly toward the Somali coast, while its eastern filament remained stationary. By 17 January, the western/coastal filament had dissipated, while the eastern one protracted southwestward along the Somali coast, forming a narrow (20-50 km wide) filament that extended from 150 km offshore of the Somali/Kenyan border near (2.0° S, 42.7° E) northeastward to (4.2° N, 53.7° E)—a total linear distance of ~1400 km. This filament persisted through 19 January, when a patch of increased luminosity developed offshore, to the northeast—commencing Phase 2 of this event.


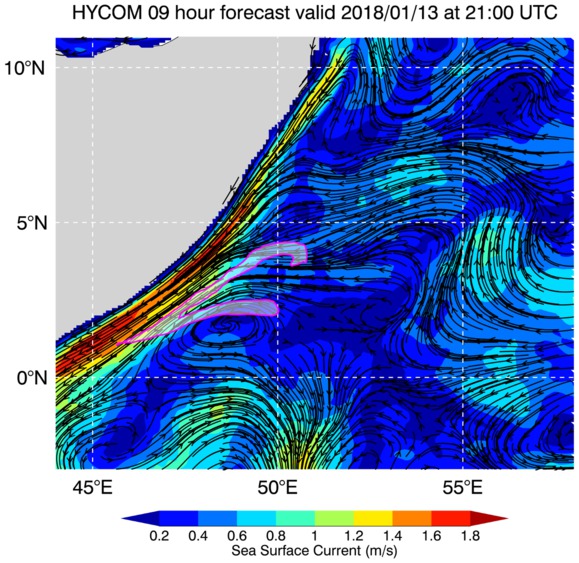


*Figure S2.7.2* *HYCOM sea surface currents analysis valid at 2100 UTC on 13 January 2018.*

Comparing the DNB imagery against HYCOM data (Figure S2.7.2), the Phase 1 luminous body coincided with the eastern boundary of strong surface currents (~1.8 m/s) flowing southwest along the Somali coast (per the Winter Monsoon circulation pattern). It appears that each arm of the *wishbone* flowed southwestward, channeled by the coastal flow and an offshore eddy centered near (1° N, 49° E) on 13 January.


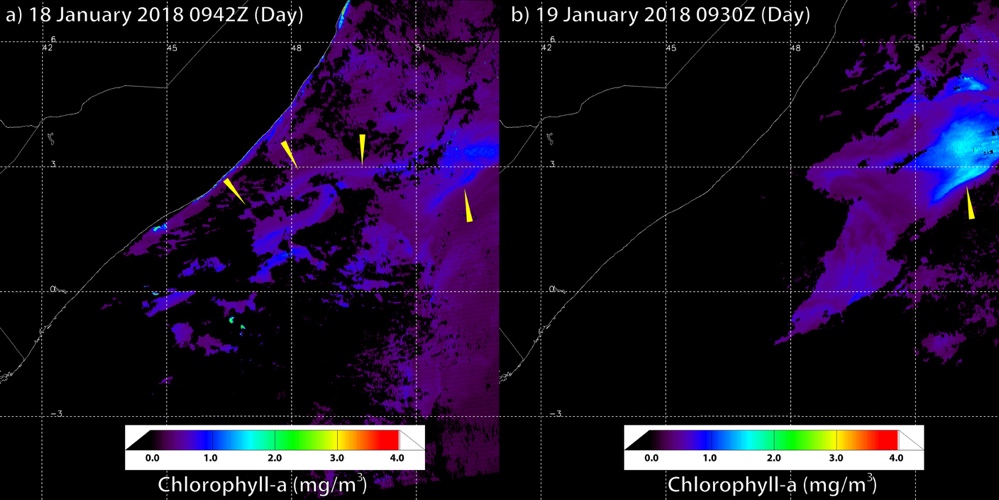


*Figure S2.7.3. Retrievals of Chla for 18 January (a) and 19 January (b) of the 2018 Somali Sea milky sea, Phase 1. In (a), the three leftmost yellow pointers denote a filament that aligned with the DNB-observed luminous body in Fig. S2.7.1. A region of increasing Chla offshore (right-most yellow pointer in both (a) and (b)) corresponds to the development zone of the Phase 2 milky sea event.*

Extensive daytime cloud cover over the region limited clear-sky views of the ocean surface, needed for Chla retrievals, during most of the Phase 1 period. While no remarkable structures were seen in the SST retrievals during Phase 1, there were indications on 18 January of filamentary structures in the Chla field in the same region as the DNB-observed luminous body (Figure S2.7.3), and on 19 January the skies remained clear enough to reveal more pronounced Chla feature emerging offshore, corresponding to the region of Phase 2 formation.

## 7.2 Phase 2: 19-23 January 2018

The main paper describes Phase 2 of the January 2018 event, which developed out of the northeast portion of Phase 1. HYCOM currents (e.g., S.2.7.4) showed that the Phase 2 luminous body was associated with the southeastern border of a counter-clockwise rotating eddy. The feature itself was positioned on the cool side of an oceanic front, with sharp radiance gradients on its eastern side and weak gradients on its western side.


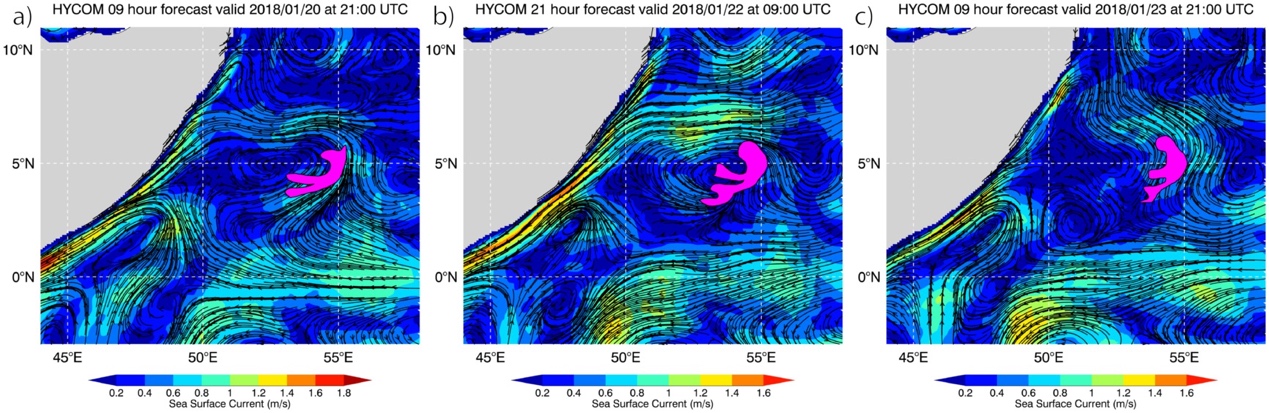


*Figure S2.7.4. DNB-identified luminous body (magenta object) overlaid upon HYCOM sea surface currents for three nights of the 2018 Somali Sea case Phase 2 event.*

As the January 2018 Somalia event was detected in real-time, we attempted to contact the crews of commercial vessels passing near it, using Global Positioning System (GPS) information obtained from Marine Traffic (marinetraffic.com). Two ships responded to our inquiries, their tracks shown in Figure S2.7.5. The first, *Anangel Maritime Service* (MMSI 240980000), crossed just to the east of the luminous body over the period 19 January 1655 Z to 20 January 0220 Z. Its crew reported that their ship produced a long (multi-km) trail of disturbed water (dinoflagellate variety) bioluminescence in the wake of the vessel while in the vicinity of the DNB-observed milky sea, but they observed no steady brightness in the waters surrounding their ship (*Capt. Babis Kouvakas, personal communication*). The second ship, the *African Puffin* (MMSI 357578000), crossed to the northwest of the feature on 21 January 2355 UTC to 22 January 0215 UTC. Its crew did not log any entries concerning bioluminescence en route (*Capt. Hitendra Chauhan, personal communication*). It is not clear whether the radiance levels of this event would have been sufficient for human detection.


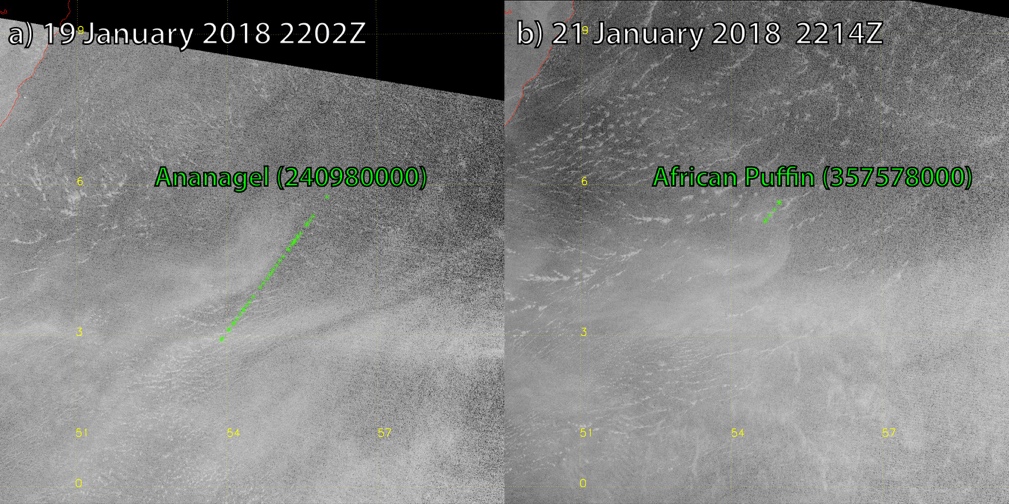


*Figure S2.7.5. Tracks of responsive cargo ships that crossed near the DNB-observed 2018 Somali Sea milky sea, according to marine traffic GPS data. The Anangel (a) reported their ship producing a bioluminescent wake, but they did not observe the milky sea along their route. The African Puffin (b) did not record unusual bioluminescence in its ship logs for this date.*

In addition to these near-misses, Marine Traffic data indicated that several ships may have indeed crossed directly over the brightest portion of the 2018 Somali Sea luminous body. Those included *ANGEL 206* (MMSI 356893000) which crossed the body on 20 January, the *Venture Harmony* (MMSI 636016892) on 21 January, and the *Lemessos Lion* (MMSI 210944000) and *UNI FLORIDA* (MMSI 357786000) on 22 January. Figure S2.7.6 shows the tracks for these four ships, plotted atop DNB imagery of the luminous body on those respective nights. We were unsuccessful in our attempts to connect with crew from these vessels.

**
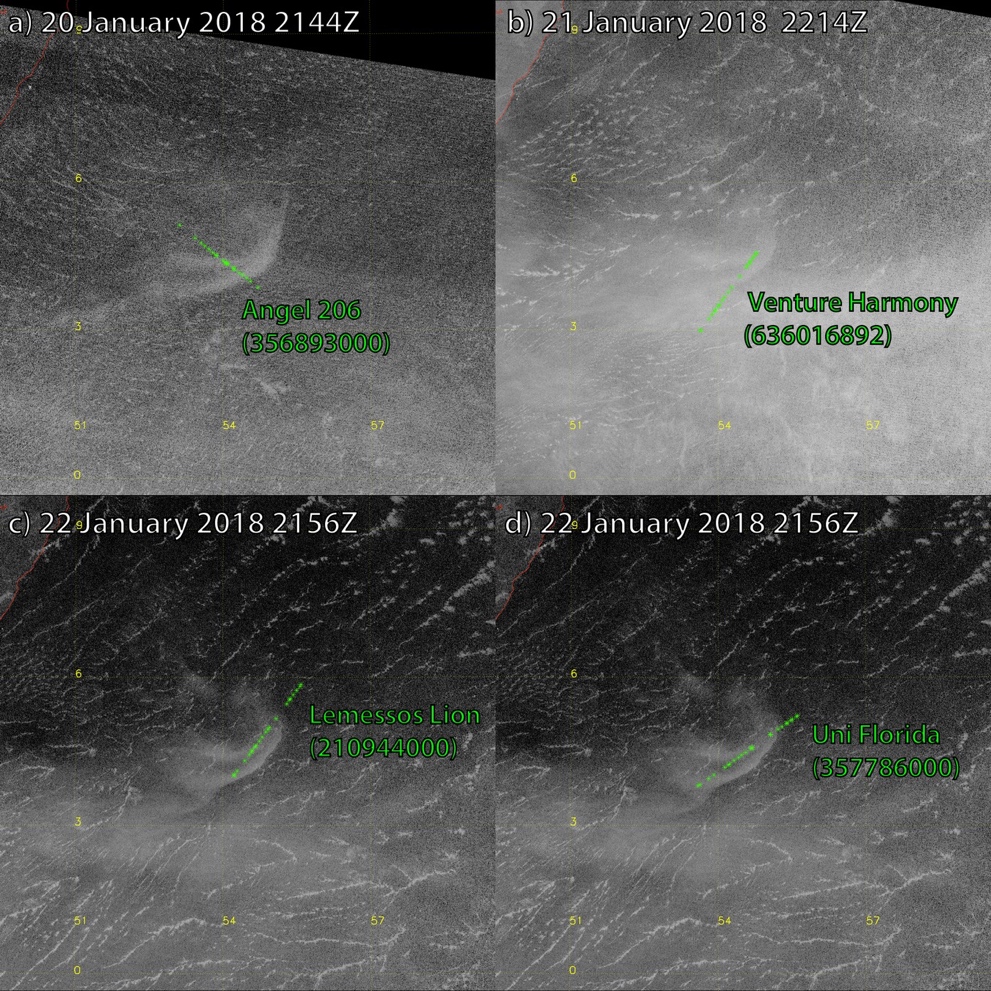
**

*Figure S2.7.6. Tracks of commercial ships that crossed directly atop the DNB-observed 2018 Somali Sea milky sea, according to Marine Traffic GPS data: a) Angel 206 on 20 January, b) Venture Harmony on 21 January, and Lemessos Lion (c) and Uni Florida (d) on 22 January.*

# 8. Somali Sea, 28 January – 7 February 2019

The onset of this case bore strong resemblance to the initial structures of the 2015, 2017, and 2018 January Somali Sea cases—suggesting a recurrent process of milky sea development in this area. While this case involves both near-coast and offshore development, the features were present on the first night of available observability in the moon-free portion of the lunar cycle for the DNB. As such, it is not clear whether two-phase sequential development occurred as it did in the 2015 and 2018 cases.


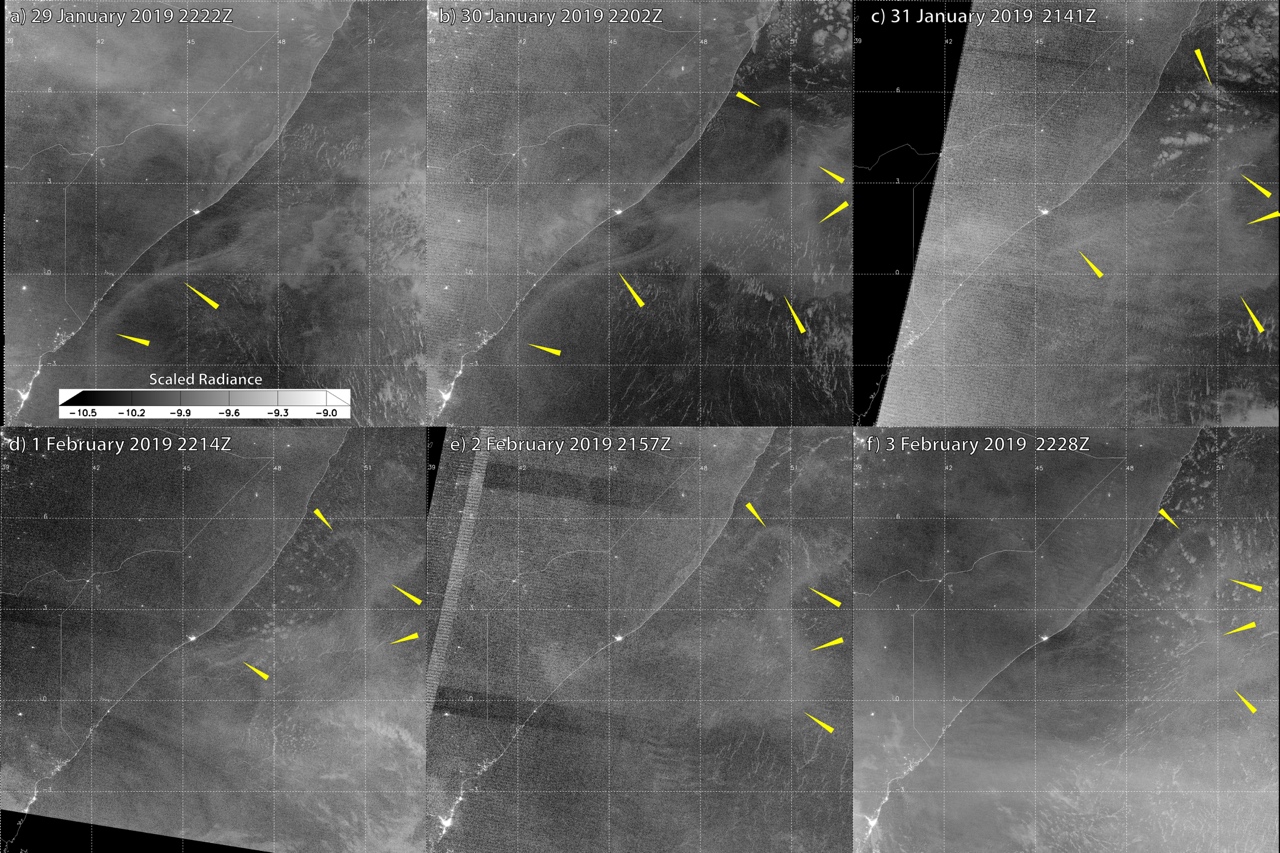


*Figure S2.8.1. A 6-consecutive night sequence of Day/Night Band imagery shows a large, persistent luminous body offshore of Somalia (yellow pointers) which did not correspond to the meteorological cloud field.*

Figure S2.8.1 shows selected imagery from a 6-night sequence of this case. On 28 January, the first night of DNB observability, a linear ~40 km wide luminous body which did not correlate with the cloud field appeared in the DNB imagery. Its southern extremity was located ~70 km offshore of the Lamu Archipelago, Kenya. Progressing northward, the body curved clockwise toward the Somali coast, approaching 40-50 km of shore before paralleling the coast for ~100 km. Near (0.5° S, 43.0° E) the body veered offshore to the northeast and terminated near (2° N, 48.5° E). The estimated area of this body was 55,000 km^2^. On the nights of 29 January – 1 February, it propagated slowly west/northwest, toward the coast, and may have reached the Somali/Kenyan shoreline as a faint luminous filament by 1 February.

This coastal luminous body connected into a much larger body offshore and to the north, which appeared in the shape of a “*hook*” at its northern extremity. On its southern end this offshore body broadened, and its exact boundaries were more difficult to identify. This large offshore structure persisted for the full duration of the event, and developed on its southern end a ~750 km long narrow tail that extended to the southwest and terminated near (2.5° S, 47.0° E). Its total area approached ~100,000 km^2^. Both the coastal and offshore luminous bodies were no longer discernible after 7 February due to several nights of strong airglow and cloud cover, followed by onset of the moonlit portion of the lunar cycle.


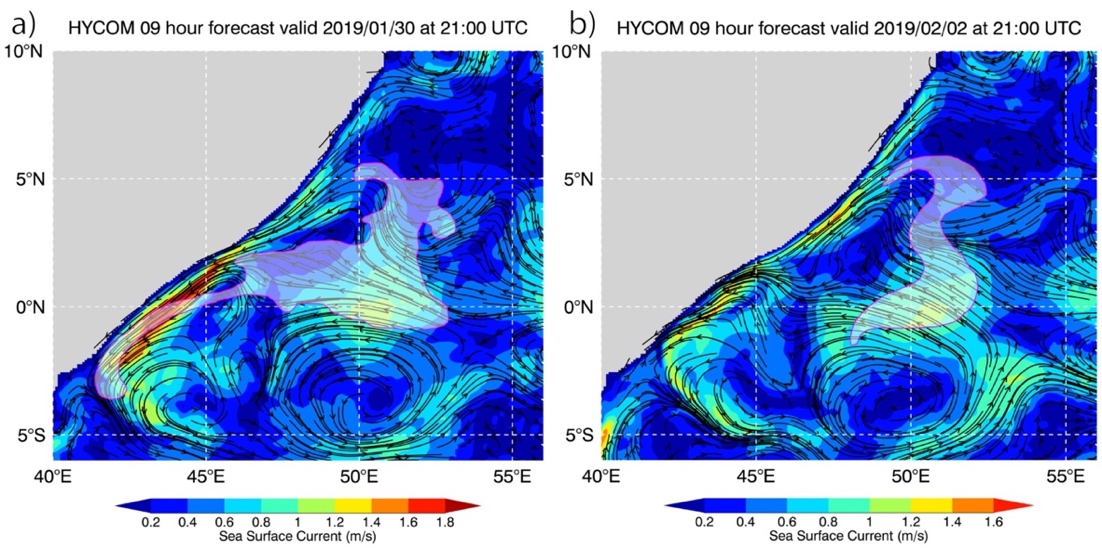


Figure S2.8.2 *HYCOM sea surface currents analysis valid at 2100 UTC on 30 January (a) and 3 February (b) 2019.*

HYCOM currents data (e.g., Figure S2.8.2) showed strong (1.5 – 2.0 m/s) southwest-flow along the coast. The linear coastal luminous body resided on the eastern boundary of this current. A broad region of the easterly equatorial current, present immediately to the east of the luminous body, could explain the body’s observed drift toward the Somali/Kenyan coast over 29 January – 1 February. The general structure of HYCOM currents in the region of the larger offshore luminous body remained mostly unchanged over the 1-7 February period. The counterclockwise swirl of currents near (5° N, 50° E) was consistent with the location and orientation of the northern *hook*-shaped luminous body.


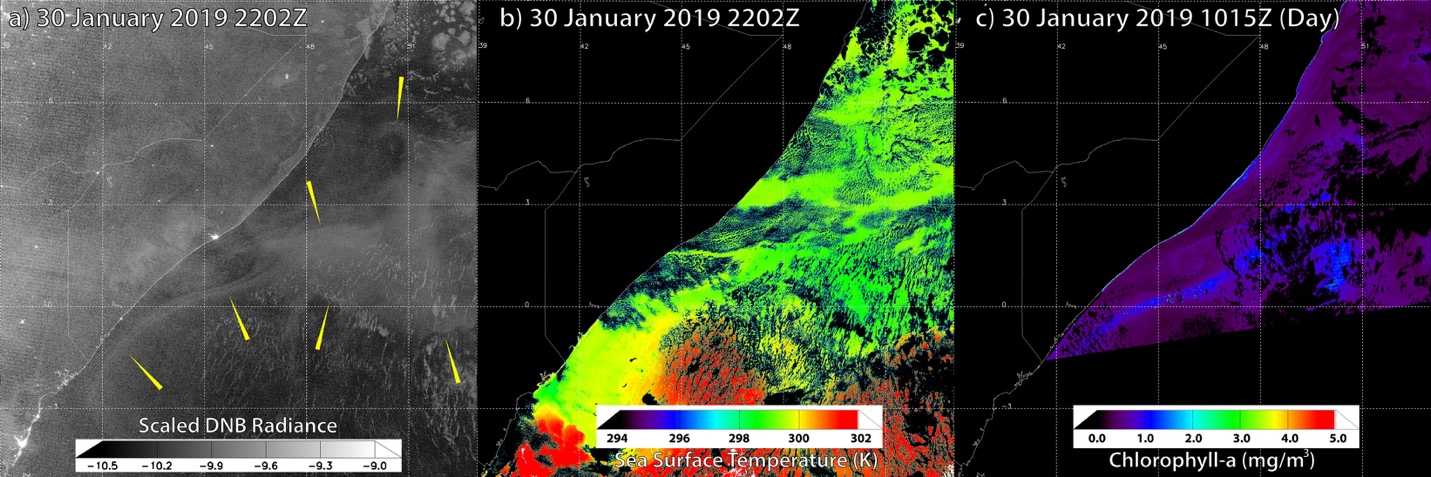


*Figure S2.8.3. Comparison of DNB imagery (a) with matching retrievals of SST (b) and Chla (c; daytime) for the 2019 Somalia milky sea on 30 January. The luminous body seen in DNB imagery (yellow pointers) shared structural similarities to a tongue of relatively cooler SST and slightly elevated values of Chla.*

SST and Chla analyses (Figure S2.8.3) show structure similar to that of the luminous body. As with other cases analyzed here, the body coincided with SST near 298 K and slightly elevated values of Chla in the 0.5-1.0 mg/m^3^ range. Once again, the brightest portions of the luminous body were adjacent to, not atop, areas of elevated Chla. This adjacency was particularly evident in the northern portion of the offshore *hook*-shaped luminous body, where elevated Chla formed a swirl along the inner boundary of the body. The southeastern portion of the offshore luminous body coincided with a sharp SST gradient of ~4 to 5 K (i.e., a possible oceanic front).

This event was detected in real time, and efforts were made to obtain eye-witness sightings. In this case, we reached out to NASA Johnson Space Center, who communicated the description and coordinates of this milky sea to the crew of the International Space Station. Unfortunately the orientation of ISS orbital tracks precluded nighttime viewing of this region during the period of the event.

# 9. Java Sea, July-August 2019

## 9.1 Java Sea Phase 1: 25 July – 9 August 2019

Phase 1 of the 2019 Java milky sea is described in the main paper. Additional analyses of this case, by far the most significant of those encountered in this study, are provided here. According to HYCOM ocean surface currents, the luminous body resided in a doldrums between westbound coastal currents along the Java coast to its north and a pair of counter-clockwise (warm-core in the southern hemisphere) eddies straddling to its southwest and southeast. These eddies drifted slowly (~0.2 m s^-1^) westward with the South Equatorial Current over the 2-week observation window, with the luminous body drifting along between them. On 29 July, a southern appendage detached from the main luminous body. It was entrained in stronger north-westerly currents of the western eddy, accelerated, and stretched into a narrow filament. Over the period 6 - 9 August, a sinusoidal wave pattern developed on the northwest extremity of the luminous body.

1. *Parallax Analysis*

Multi-angle views from the S-NPP and NOAA-20 satellites assisted in differentiating the luminous body from ephemeral airglow emissions and clouds reflecting airglow. These dual views also enabled a parallax-shift analysis, useful in assigning the luminous body to the ocean surface and distinguishing it from clouds/airglow. Figure S2.9.1 compares a pair of opposing perspectives from S-NPP and NOAA-20 of the region containing the luminous body on 3 August 2019. These DNB imagery were separated by ~50 min. Percent differences between the two images show zero change in location for the luminous body, in contrast to obvious shifts in the moving/evolving cloud field as well as changes in airglow brightness, which increases toward scan-edge due to the van Rhijn effect. The zero-parallax shift of the luminous body assigns it geometrically to the ocean surface.


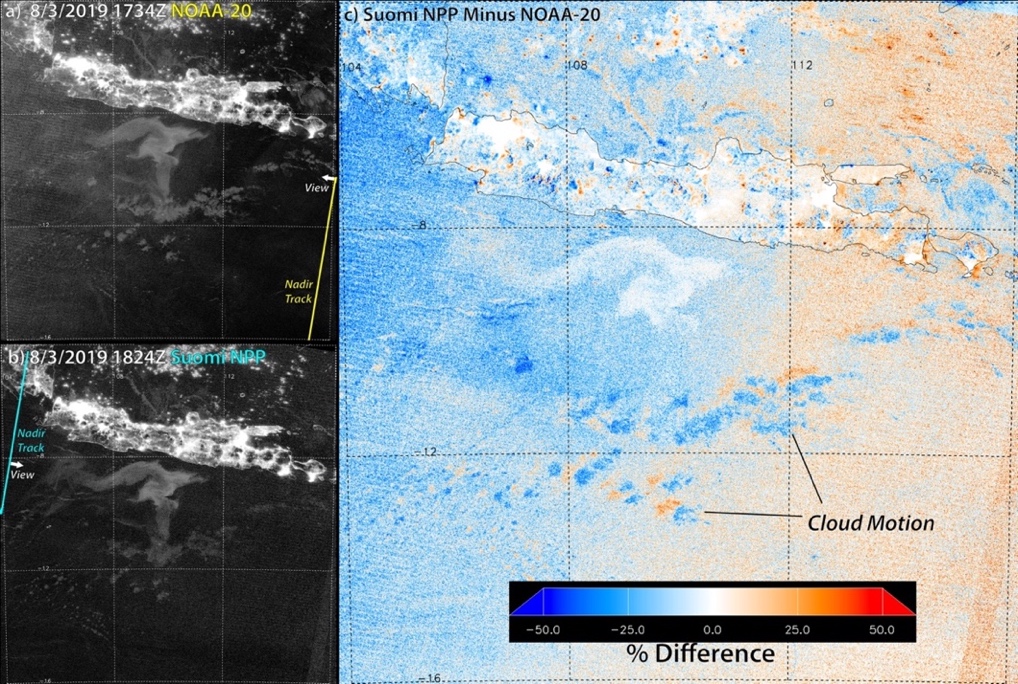


*Figure S2.9.1. Dual views of the Java Phase 1 milky sea on 3 August 2019 from a) NOAA-20 (1734 Z), b) Suomi NPP 50 min later (1824 Z), with satellite nadir ground tracks shown, and c) the percent difference in scaled brightness between the two images. Positive/negative changes between the two images appear as red/blue, and zero change is shown in white. Whereas clouds display significant shifts due to both parallax shift and motion, the luminous body produces zero shift, including at its edges—confirming that it is a surface-based feature. Negative and positive biases on the left/right of the image are due to the van Rhijn effect of airglow.*

1. *Ship Lights Analysis*

During Phase 1, DNB imagery showed that multiple vessels, identifiable by their lights (which appear as bright points in both the *Suomi*-NPP and NOAA-20 DNB imagery) resided within and around various parts of the luminous body on several nights. In particular, one vessel was observed to move slowly westward amidst the luminous swirl over three consecutive nights from 29 July (8.80° S, 108.90° E) to 1 August (8.77° S, 108.73° E). A second vessel appeared on 2 August (8.89° S, 108.67° E) – 3 August (8.98° S, 108.49° E). The two boats straddled the interface of a sharply-defined boundary between the milky sea and darker, non-luminous waters. This positioning could signal their awareness of the luminosity either directly (visually) or indirectly by proxy of favorable fishing conditions near its boundary (e.g., the fish-attracting properties of the glowing waters).

The ship lights (Figure S2.9.2) provide evidence of the luminous body being tied to the ocean surface and not being a lower-atmospheric cloud feature. As described in *Methods*, under clear-sky conditions ship lights appear in nighttime DNB imagery as distinct points of bright light, indicative of minimal atmospheric scattering. However, in cases where a ship light resides beneath a cloud layer or within fog, its upwelling light will be scattered and diffused by interaction with the cloud particles, imparting a blurred effect to the point source as observed in DNB imagery. On numerous nights of Phase 1, ship lights residing within the luminous body appeared as distinct points of bright light, despite the surrounding water brightness being consistent with an optically thick cloud deck that would have presumably diffused those lights considerably. Specific ship lights illustrating this effect are denoted with turquoise arrows in Fig. S2.9.2. Ship lights that were in fact beneath clouds are shown at the red-arrowed locations. The lack of blurring for the former light suggests that the brightness seen around these vessels originated from the ocean surface.


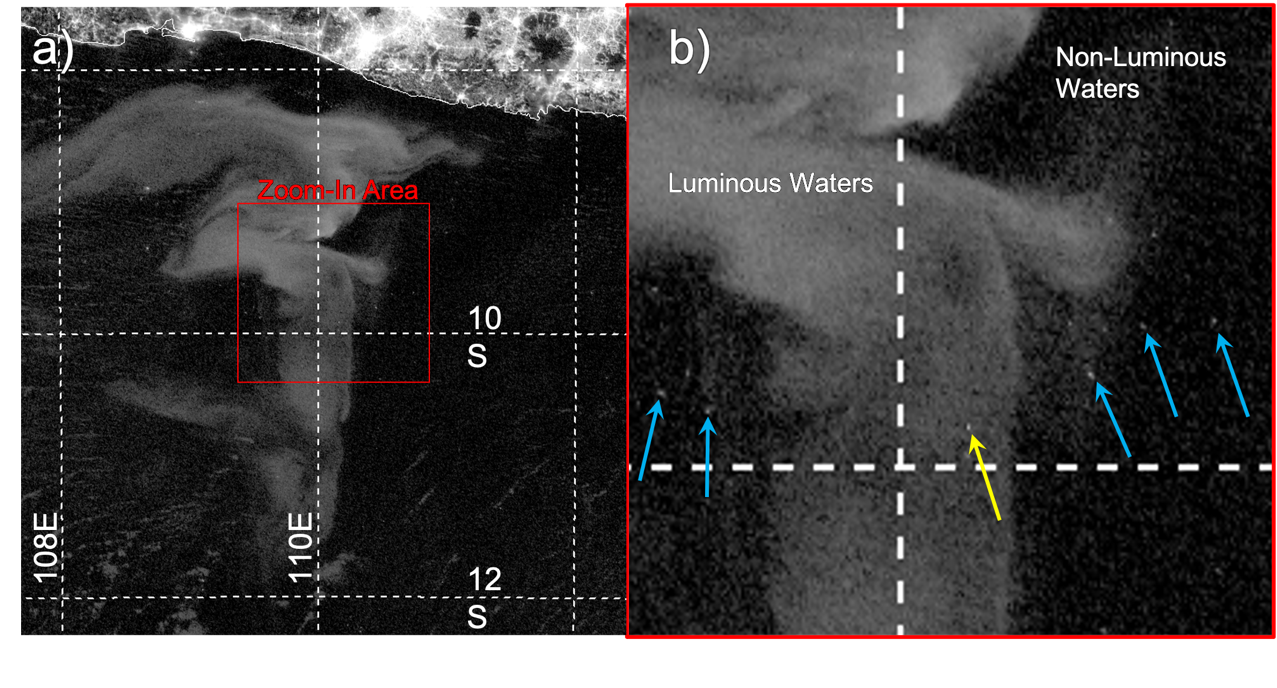


*Figure S2.9.2 View of the 2019 Java Phase 1 milky sea on 2 August 2019 1753 UTC (a) and inset zoomed-in area (b), where arrows denote the locations of selected individual ship lights. Turquoise arrows identify ship lights under clear sky conditions, removed from the luminous waters. Yellow arrow denotes a ship light amidst the luminous waters, remaining as a distinct point source.*

1. *Radiometric and Ocean Surface Properties*

Over 29-31 July, a ~500 km^2^ sub-region of the luminous body near (9.05° S, 110.30° E) attained particularly high radiance values compared to the airglow and starlight-reflecting clouds. Close inspection of this bright sub-region revealed two small, isolated cumulus clouds (identified in IR imagery), appearing in *dark relief* against the brighter surrounding luminous background (Figure S2.9.3). Other clouds in the scene, reflecting downwelling airglow and starlight, appear relatively bright against the darker ocean away from the luminous feature, or are similar brightness to the luminous surface and thus produce small/indiscernible contrast. The example gives further evidence that the light source is coming from the ocean surface. The illumination of clouds from below contributes to the ‘*photo-negative*’ effect described in some milky sea encounters. This was the only case encountered in this study where the surface was of sufficient brightness to produce this effect.


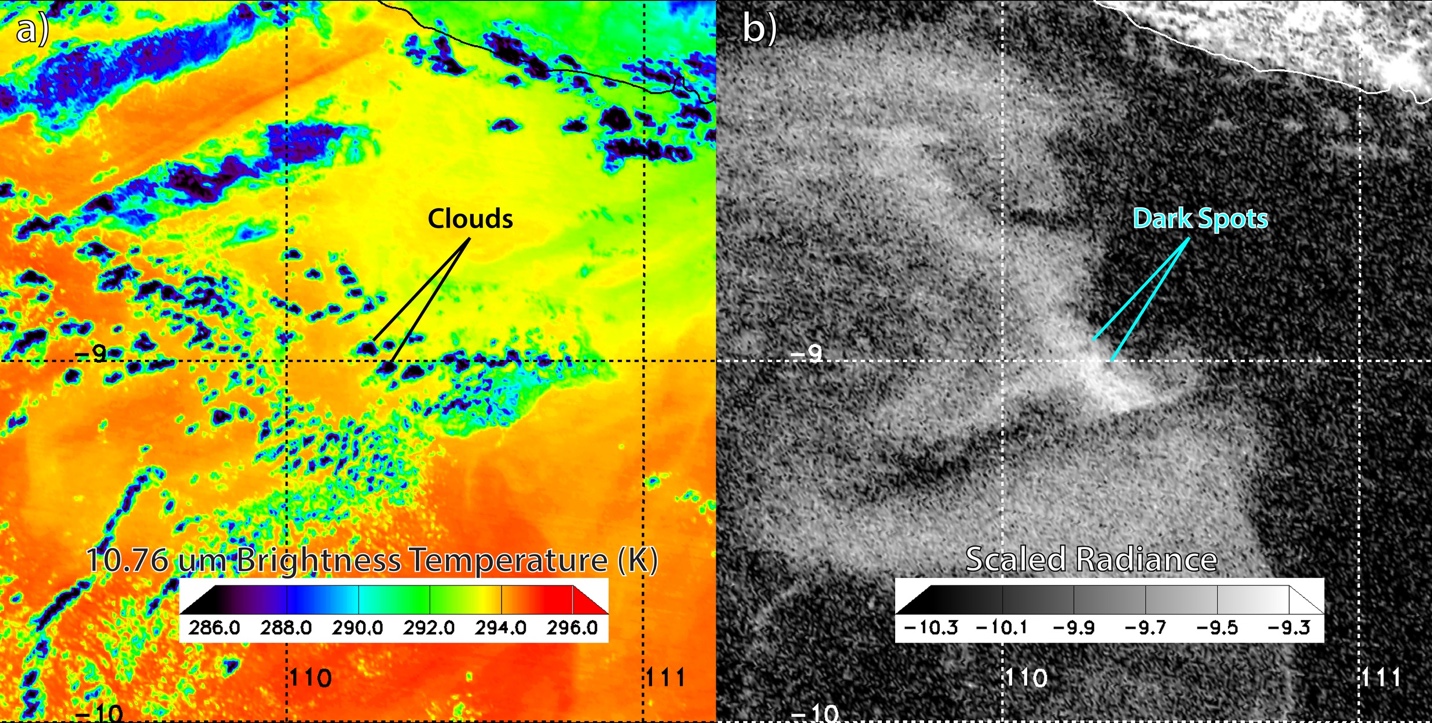


*Figure S2.9.3. VIIRS imagery from the 2019 Java Phase 1 milky sea on 30 July. Panel (a) shows clouds as seen in thermal infrared imagery, which correspond to dark spots in Day/Night Band log_10_-scaled (W cm^-2^ sr^-1^) radiance in Panel (b). These clouds attenuated the relatively bright upwelling light from below.*

In many of the cases documented here, the luminous bodies remained partially detectable even in the presence of overriding clouds—producing a slightly elevated brightening of the cloud field in a pattern matching the body on adjacent clear-sky evenings. This detectability is due to the conservatively scattering nature of clouds at visible-light wavelengths, allowing for diffuse upwelling transmission. City lights also appear regularly in nighttime DNB imagery in overcast conditions for this same reason; their light diffusing through all but the most optically thick of clouds. Cirrus clouds are optically thin at visible wavelengths and highly forward scattering, rendering them nearly transparent to surface-based light sources.


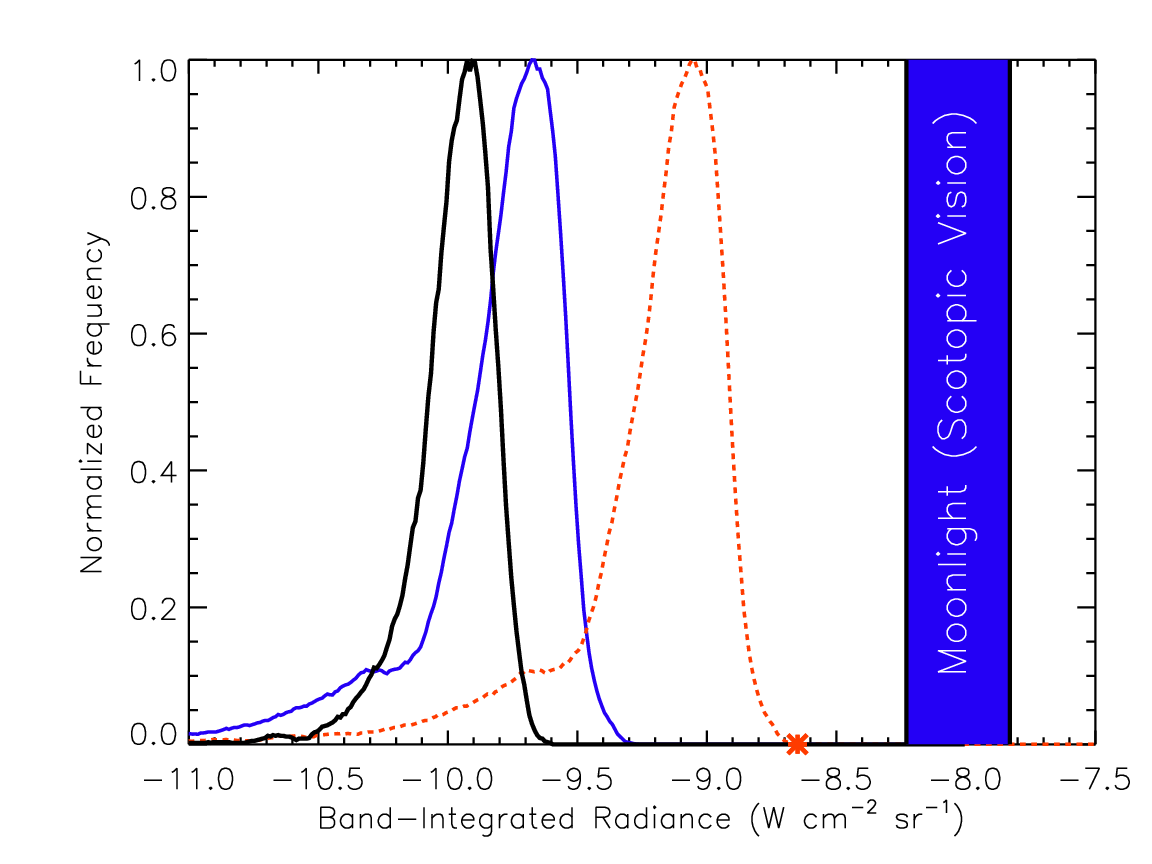


*Figure S2.9.4. DNB log_10_-scaled radiance distribution of airglow-reflecting clouds (black) and a bright sub-region of the Java Phase 1 luminous body (blue; corresponding to the sub-region highlighted in Fig. S2.9.3). A radiance distribution adjusted to scotopic-vision (based on a 4.15 multiplier) is shown as a red-dashed curve, and the lunar disk radiance to human scotopic vision is shown in the blue box, for reference. The red asterisk denotes the brightest (= -8.65) pixel of the luminous body in scotopic-adjusted response—corresponding to 2.24 × 10^-9^ W cm^-2^ sr^-1^ in scotopic response when assuming a V. fischeri luminous bacteria source.*

Corresponding distributions of radiance for the brightest sub-region of Fig. S2.9.3, as well as the surrounding cloud field, are shown in Figure S2.9.4. When adjusted for human scotopic vision response, the brightest portions of the luminous body sub-region (where the photo-negative effect was discerned in Fig. S2.9.3) attained values ~38% of a full moon’s brightness. Under astronomically dark conditions, the ocean surface at such emission levels may have appeared noticeably bright to dark-adapted human vision—perhaps even achieving the classic ‘*snow field’* descriptions of milky sea eye-witness accounts detailed in *Supplementary Discussion 1*.


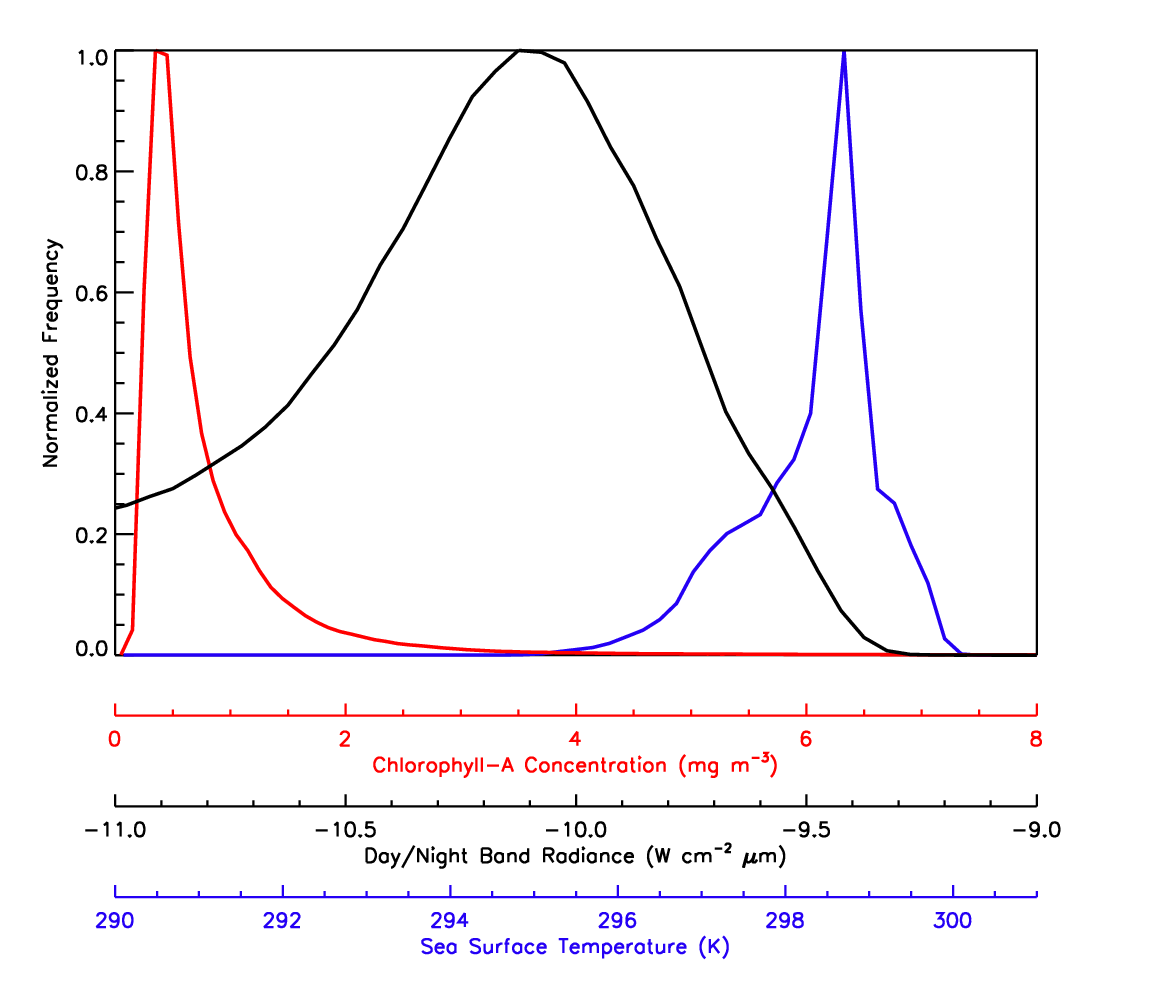


*Figure S2.9.5. Normalized distributions of Chla, SST, and DNB log_10_-scaled radiance for the 2019 Java Phase 1 (25 Jul – 9 Aug 2019) milky sea.*

Figure S2.9.5 shows distributions of Chla, DNB Radiance, and SST for a box centered on the entire Java Phase 1 luminous body for the full duration of its observation (25 July – 9 August). Characteristic ranges of SST and Chla over the domain were [298 – 299 K] and [0.5 - 1.5 mg/m^3^], respectively, and were correlated inversely—lower SST (and upwelling, nutrient-rich waters) corresponding to higher Chla. In comparison to Fig. S2.9.4, the main radiance contributions from the luminous feature comprise the right-tail of the DNB radiance distribution.

1. *Radiance, SST, and Chla Parameter Cross Sections*

The spatial distribution of DNB radiance structure vis-à-vis SST and Chla, while complex, contained some distinct correlations giving insight to the characteristic environment of milky seas. Figure S2.9.6 shows cross sections through the center of the 2019 Java Phase 1 luminous body on 2 August, which reveal a general relationship between higher DNB radiances and higher values of Chla, and decreasing SST. The meridional (cyan) cross section traverses part of Java between 7-8° S and its artificial lights, showing how a brighter portion of the luminous body near 9° S rivaled the radiance level of those lights. The zonal (yellow) cross section, cutting through the northern flank of the luminous body, showed strong positive correlation between DNB radiance and Chla, and a weaker inverse correlation with SST. Animations of these meridional and zonal cross sections, traversing the full domain of Fig. S2.9.6, are provided as Supplementary Movies 13 and 14.


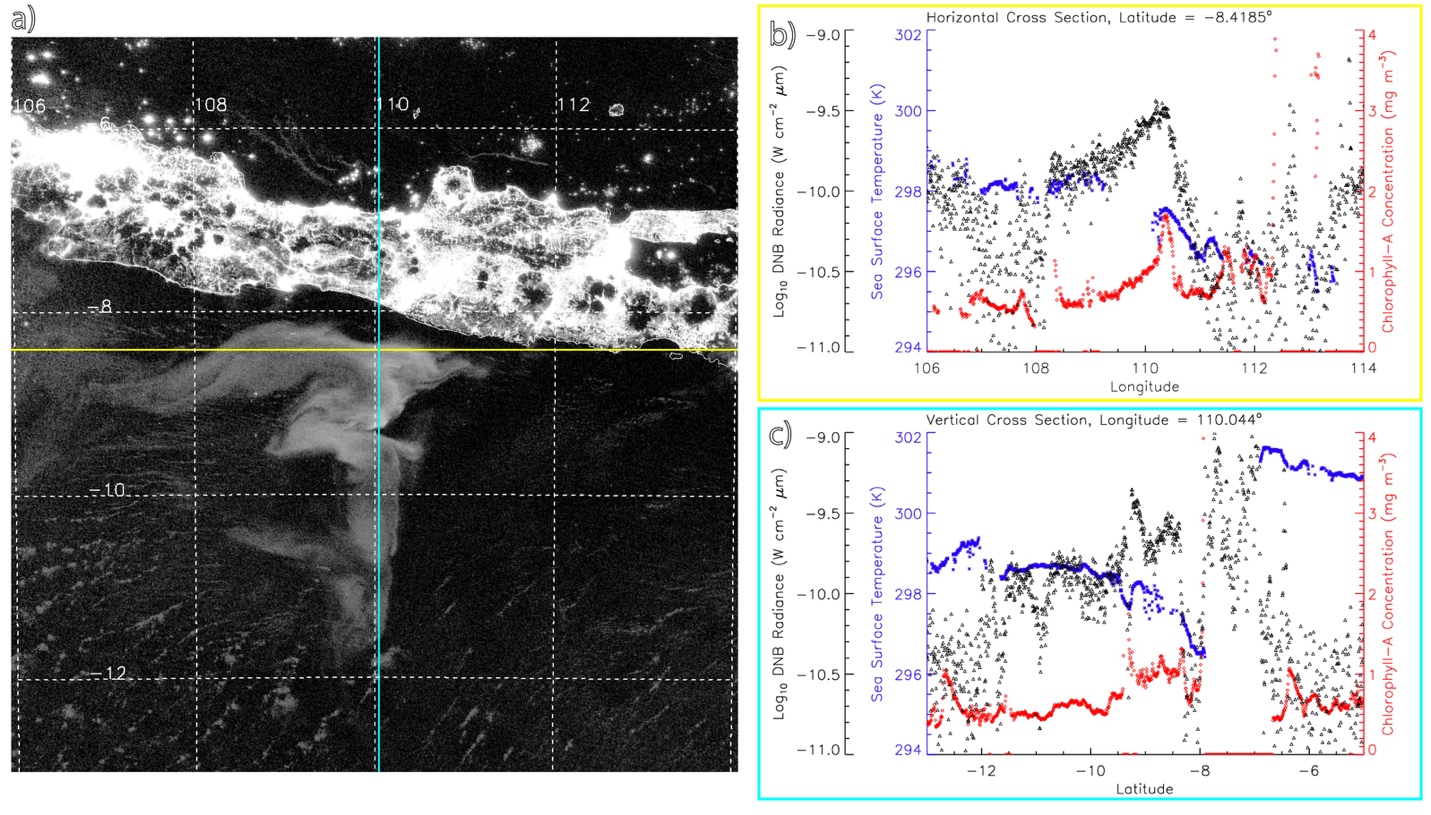


*Figure S2.9.6. a) 2019 Java Phase 1 milky sea on 2 August at 1753 UTC, with colored lines denoting zonel (b) and meridional (c) cross sections of DNB radiance, SST, and Chla. Animations of these cross sections, spanning the entire scene of panel (a), are provided as Supplemental Movies SM2.9.1 and SM2.9.2.*

1. *Salinity*

As mentioned in the main paper, the growth rate of the luminous bacterium *V. harveyi* is negatively correlated to sea surface salinity and positively correlated to SST. Sea surface salinity fields from HYCOM were also used to analyze the water state for the 2019 Java Phase 1 milky sea. Figure S2.9.7 shows an example of luminous body match-up with the sea surface salinity for 2 August 2019. The values within the luminous body waters fell in the 34.2 - 34.3 practical salinity unit (PSU; equivalent to parts of salt per thousand parts of water), which is lower the globally averaged ocean salinity of 35.5 PSU whose range spans from 15 near river mouths to 45 in the Dead Sea salt lake. Animations of these salinity data over the entire Phase 1 event showed that the luminous body tracked within a water mass having little variation in sea surface salinity. The uniformity offers support to the natural flask hypothesis postulated in the main paper.


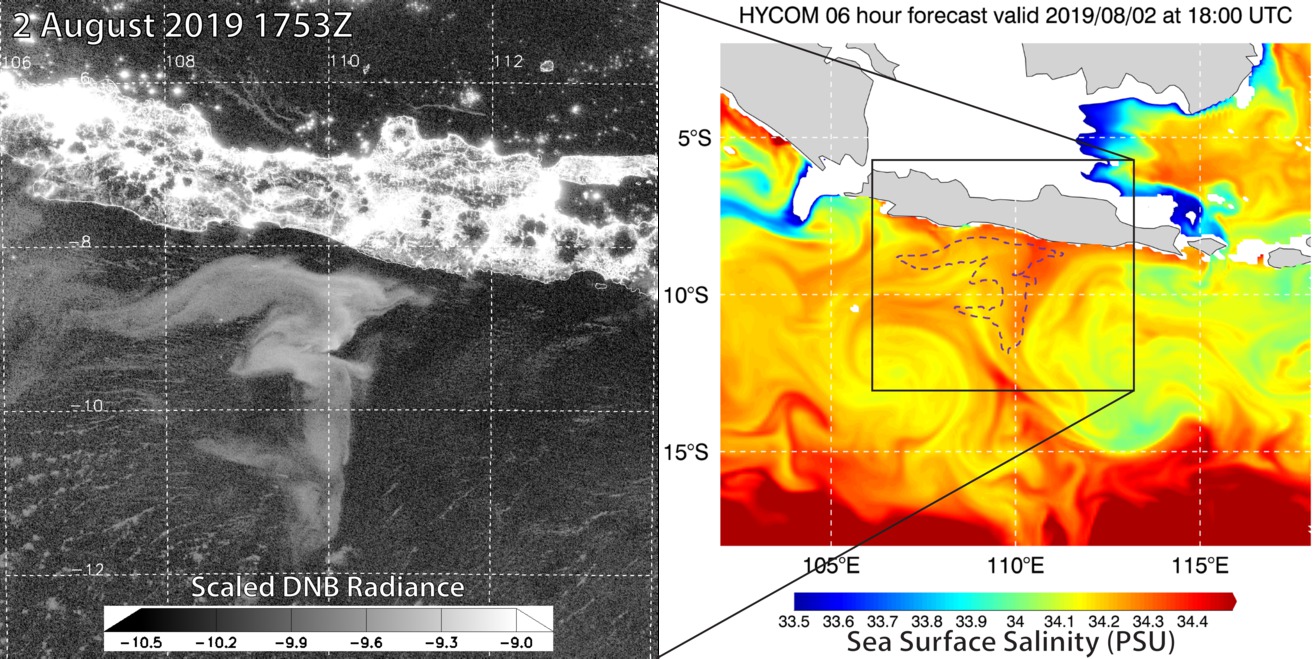


*Figure S2.9.7. Comparison of 2019 Java milky sea DNB imagery (left) and HYCOM sea surface salinity (right) for ~1800 UTC of 2 August 2019. The approximate perimeter of the luminous body seen in DNB imagery is overlaid upon the salinity field.*

The Java and Bali Seas are characterized by fresh waters from local rain runoff from Kalimantan (Indonesian part of Borneo island). These waters enter the eastern Indian Ocean, near the area of where the 2019 Java milky sea was observed, through the Sunda and Bali straits west and east of Java, respectively. Additional fresh waters from further east in the Maritime Continent, including the Banda Sea, move southward via the Indonesian Throughflow (ITF) via the Lombok, Ombai, and Timor passages. These waters then flow east-to-west and south of Java. While the PSU values for the luminous body were not anomalously low (which might promote faster bacterial growth), additional study of the history of these waters is necessary. The potential role of freshwater-stimulated luminous bacterial growth in the 2019 Java event is a subject of ongoing research, as part of a broader study into milky sea formation mechanisms in general, and those of the Maritime Continent in particular.

1. *Ocean Surface Deformation Fields*

Figure S2.9.8 contains results of a deformation field analysis (following Equations 1-6 from Section 5.2 above), showing that for the central part of the Java Phase 1 luminous body the total strain (which drives deformation), effective Coriolis parameter (which drives rotation), and current speeds were all small—indicating that these waters were quiescent. The boundaries of the luminous body, particularly on the eastern edge, abutted with enhanced total strain and effective Coriolis parameter, implying the shape of luminous body would vary over time through tilting and stretching, as was observed. Also matching to the DNB-observed structure was a wave train (best seen in Fig. S2.9.8c-d) aligning with the northwestern sinusoidal pattern seen on 6 - 9 August 2019 in the DNB imagery. Despite its complex distribution and peripheral influences, the main quiescent region of luminous waters was largely isolated from the surrounding flow patterns, such that lateral mixing was inhibited. The deformation fields support the idea of a natural flask as a possible formation environment for this milky sea.


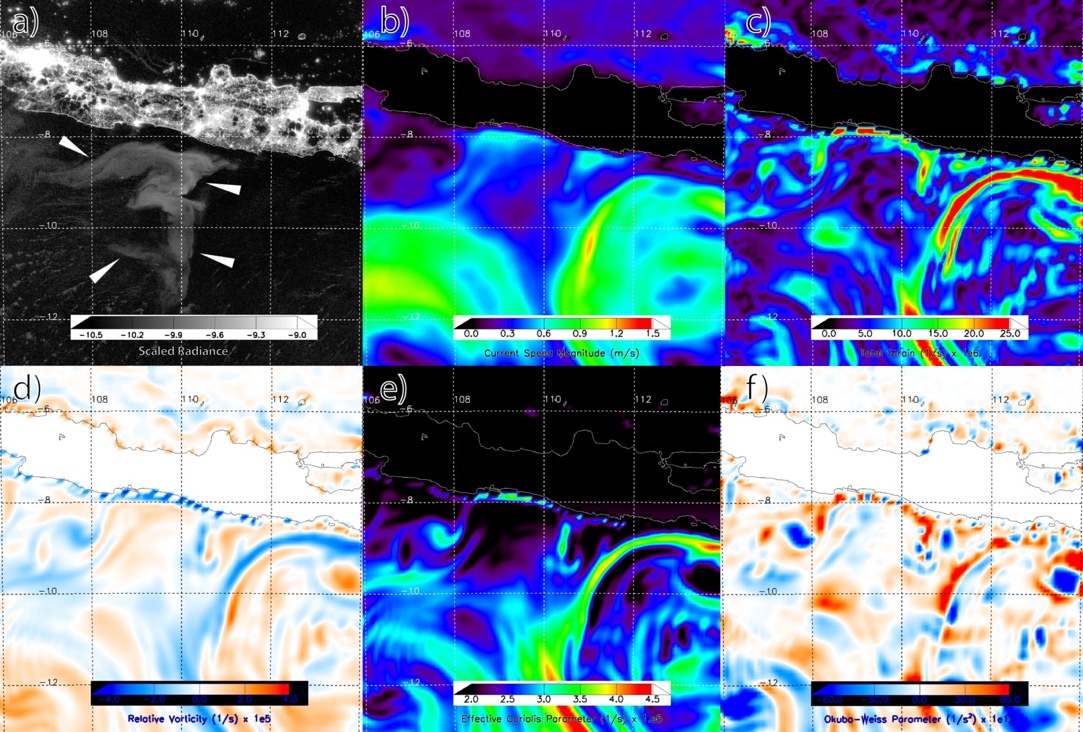


*Figure S2.9.8. VIIRS Day/Night Band imagery from 2 August 2019, 1753Z (a; white pointers denote the location of the luminous body) Java Phase 1 milky sea, along with b) HYCOM analyzed surface current speed, c) total strain, d) relative vorticity, e) effective Coriolis frequency, and f) the Okubo-Weiss parameter.*

1. *DNB vs. OLS Low-Light Visible Imagery—Tying Past to Present*

Given the previously confirmed ability of DMSP/OLS to detect a visibly bright (to human observers) milky sea, albeit at the very fringe of the instrument’s sensitivity, an especially germane question to the current study was how legacy OLS imagery compared against these DNB-detections. The 1995 *S.S. Lima* case provides a direct linkage between the appearance of OLS imagery vis-à-vis a milky sea that appeared significantly bright to dark-adapted (scotopic) human vision. Fortunately, comparisons between DNB and OLS were still possible at the time of the 2019 Java event, as the last OLS sensors continue to operate on the soon-to-be-decommissioned DMSP satellite constellation.

OLS data for several of the cases were extracted from Naval Research Labortory-Monterey archives with the assistance of Dr. Richard Bankert, and remapped to the same domain as the DNB, allowing for direct intercomparison. Following the same procedure as the *S.S. Lima* study, the uncalibrated OLS data (provided as digital numbers; DN) were scaled over a minimum/maximum DN range of [4, 20] to enhance the lower-third of the limited 6-bit OLS range. The OLS utilizes a dynamically varying gain, which operates in its maximum gain stage on the moonless nights of the lunar cycle. It produces imagery that is significantly coarser in spatial resolution (~5 km effective resolution reported on a 3 km grid) compared to the DNB’s 0.742 km resolution, and contains higher noise (a signal to noise ratio close to 1 where the DNB provides a value of 9). Despite these limitations, OLS data have demonstrated (via the *S.S. Lima* study) a crude ability to identify strong milky seas by the standards of human detectability. Examining the OLS-perspective on the 2019 Java event provides important context that is traceable to the human perception of milky seas.


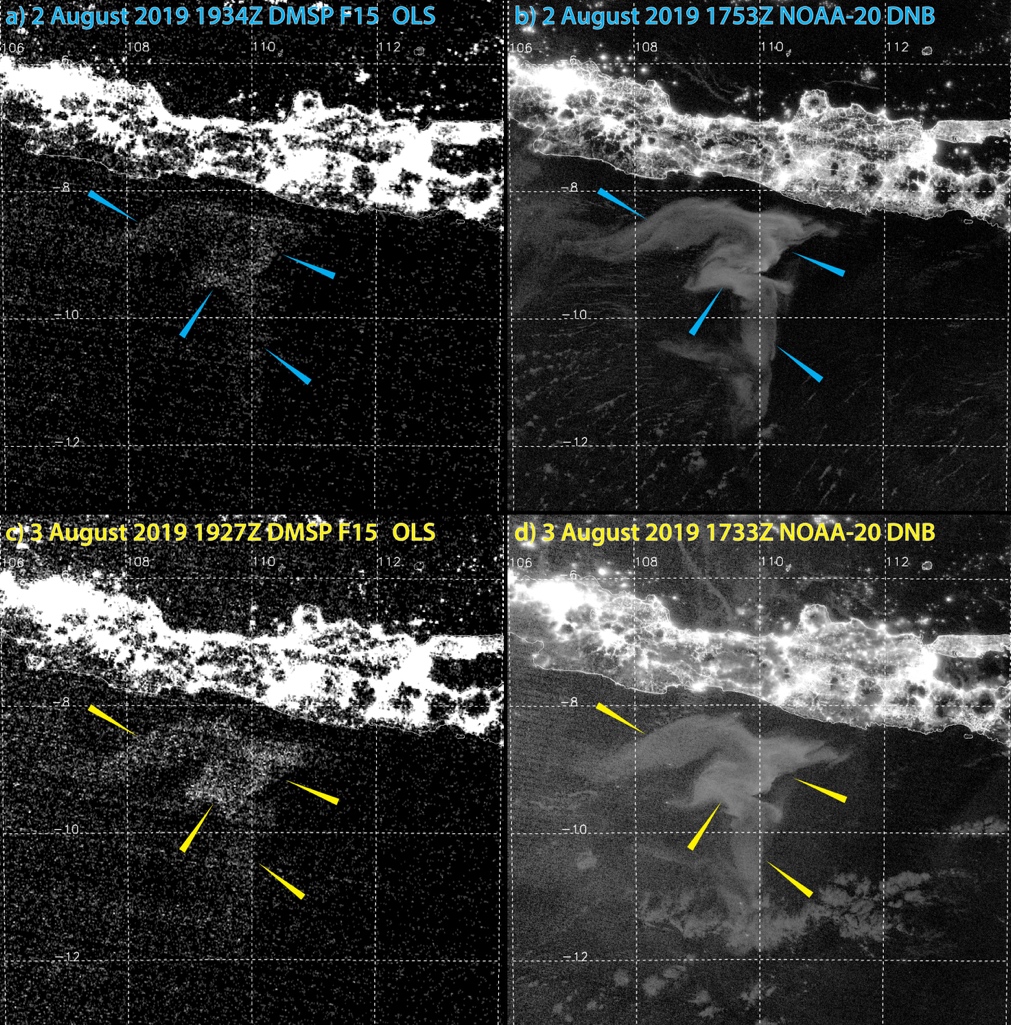


*Figure S2.9.9. Comparison of OLS and DNB enhanced low-light imagery for 2 August (a,b) and 3 August (c,d) of the 2019 Java milky sea, Phase 1. Pointers mark common locations in the imagery pairs. The OLS luminous body detection seen here is similar in digital number brightness to that of the 1995 S.S. Lima milky sea event, in which case the ocean surface appeared as a “snow field” to human observers under dark-adapted conditions.*

In the other DNB-detected cases, the very low SNR of the OLS data resulted in noise-dominated imagery, precluding its identification of luminous features. However, for the prominent 2019 Java Phase 1 milky sea event, the OLS data successfully detected a persistent feature matching the central and northern portions of the luminous body. Examples from the nights of 2-3 August 2019 are shown in Figure S2.9.9. Signals associated with the OLS-detection occurred very close to its radiometric noise floor, but were of sufficient quality to detect the luminous body and observe it evolving in the same way as the much higher-quality DNB imagery. By association with the *S. S. Lima* milky sea, which appeared at a similar OLS digital count level near the instrument noise floor, the current comparison suggests that large portions of the 2019 Java luminous body may indeed have appeared sufficiently bright to the human eye to appear as a “*field of snow*” to any observers *in situ*. This cross-reference of new-generation DNB to legacy-system OLS imagery supports human vision detectability inferences made with respect to Fig. S2.9.4, and offers a unique calibration transfer and historical connection between the 1995 and 2019 milky sea events.

## 9.2 Java Sea Phase 2: 25 August – 7 September 2019

1. *DNB Imagery*

On the night of 25 August, when the moon-free portion of the lunar cycle resumed, a thin luminous filament (~30 km wide) reappeared in the DNB imagery over the same general area as Java Phase 1. Its southern extent was located ~150 km east of Christmas Island (10.45° S, 105.69° E), and it arced in a clockwise sense northeastward toward the Java coast. DNB imagery from the night of 26 August were of lower quality due to cloud cover, but a relatively clear view on the 27^th^ revealed that the filament paralleled the coastline eastward for more than 600 km (possibly reaching the shore near 8.25° S, 111.0° E) before gradually broadening to a ~90 km width on its far-eastern end near (9° S, 113° E). By 30 August, this broadened eastern region of the coastal luminous body had evolved into a sinusoidal pattern reminiscent of the Phase 1 body’s northwestern extremity, and another narrow filament on its east side trailed offshore to the southwest for ~300 km.

The full structure of the luminous body as seen on 31 August (Figure S2.9.10) spanned a linear distance of ~1400 km and covered ~50,000 km^2^; about half the area of Java Phase 1. Its DNB-measured radiances were weaker than Phase 1 as well, but it was still of comparable intensity to the airglow-reflecting cloud field, and significantly brighter than the non-luminous adjacent waters. Thus, it was readily detectable and trackable as a contiguous luminous body over multiple nights.


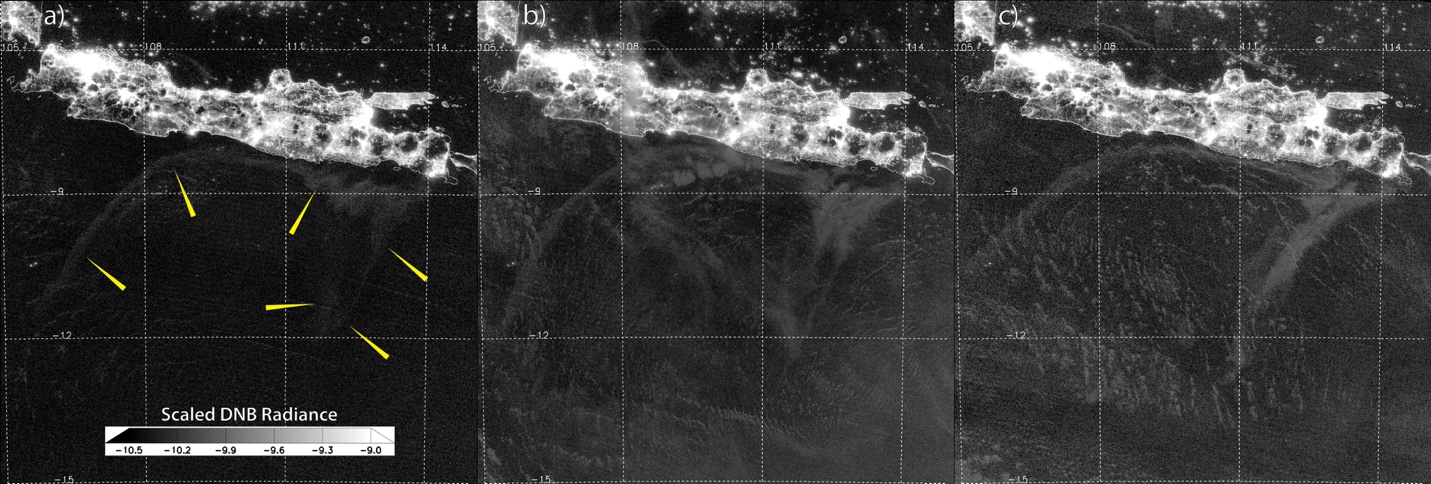


*Figure S2.9.10. Suomi NPP Day/Night Band view of the 2019 Java milky sea (denoted by yellow pointers), during Phase 2 of the event, on a) 31 August at ~1800 UTC, b) 3 September at ~1752 UTC, and c) 6 September at ~1748 UTC.*

Over the period of 1 - 6 September, the western and eastern luminous filaments drifted slowly westward while the broader northeastern area remained largely fixed in position. Based on examination of HYCOM sea surface currents, and comparisons between the Phase 2 and the Phase 1 luminous bodies, it appears that the northeastern portion of Phase 1 may have drifted eastward along the Javan coast during the unobservable (moonlight contaminated) gap between Phase 1 and Phase 2, pooling along the zone of 3° S between 111-114 ° E.

1. *Sea Surface Currents*

As in Phase 1 of this 2019 Java event, HYCOM currents (Figure S2.9.11) showed that the dominant features governing the Phase 2 body’s structure and motion were two counterclockwise-rotating eddies. The western of these was in fact the original eastern eddy of Phase 1, which propagated westward during the lunar obscuration period and by 30 August was now centered at (13.0° S, 107.5° E). A new eastern eddy in the train was positioned at (14.0° S, 116.0° E). As in Phase 1, the dual eddies drifted in unison steadily westward over the course of Phase 2 at a rate of ~18 km/day (~0.21 m/s). The western luminous filament contoured atop the northwest streamlines of the western eddy, the northern part aligned with easterly currents along the Javan coast, and the broadened northeastern luminous area resided in a doldrums of weak and variable currents. The eastern edge of this latter area became entrained into currents associated with the eastern eddy, directing its luminous waters southwestward to form into another narrow filament that mirrored the western filament (Fig. S2.9.11c), explaining the structure in Fig. S2.9.10c.


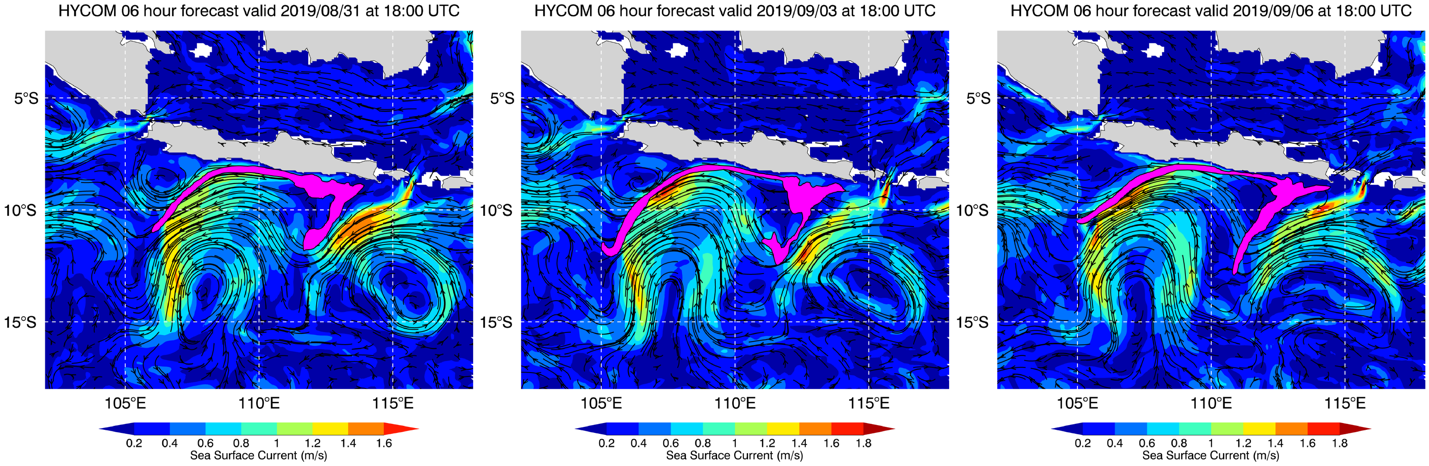


*Figure S2.9.11. HYCOM current analysis with 2019 Java Phase 2 luminous body overlaid in magenta for 31 August, 3 September, and 6 September 2019 at 1800 UTC. The southward-extending luminous filaments aligned with the northwestern quadrants of two counterclockwise-rotating warm-core eddies.*

1. *Ocean Surface Properties*

As in Phase 1, satellite retrievals of ocean surface properties (Figure S2.9.12) linked the Java Phase 2 luminous body with cooler waters and slightly elevated Chla, apparently drawn from upwelling coastal waters along the Java coast by the dual eddies. Also as in Phase 1, the luminous body itself was confined to a relatively narrow range of SST over 298 ± 1 K. And, in accord with many other cases reported on here, its most radiant waters were spatially decoupled from the highest Chla regions while maintaining adjacency and structural similarity.


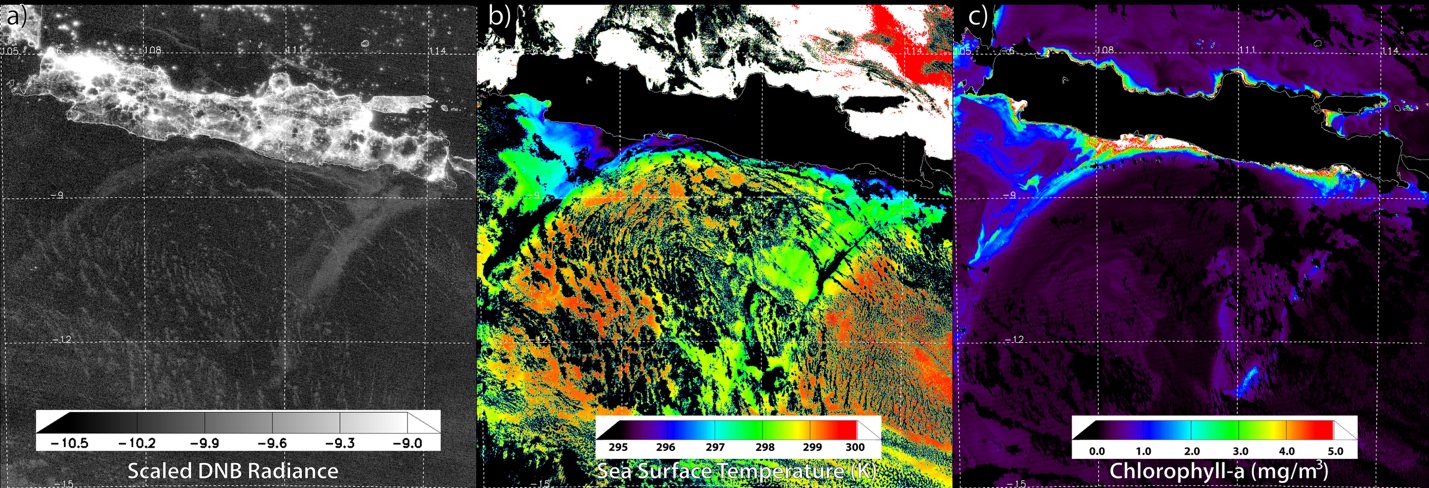


*Figure S2.9.12. Comparison on 6 September 2019 of a) DNB (1748 UTC), b) SST (K; 1748 UTC), and c) daytime Chla (mg/m^3^; 0640 UTC) retrievals, showing the association of the luminous body offshore of Java with cooler SST and moderately elevated Chla.*

1. *Chance Encounter with Island Obstacle*

A serendipitous natural experiment helped to connect the 2019 Java Phase 2 luminous body to the ocean surface. Over 4 – 5 September, the western filament intercepted Christmas Island (10.45° S, 105.69° E) during its slow westward drift (Figure S2.9.13). Per the HYCOM currents, the flow of surface waters within the filament was toward the south/southwest at ~0.6 m/s, following streamlines along the outer perimeter of the eddy located to the filament’s southeast. By the night of 5 September, the filament had already intercepted and advected past the Island. DNB imagery on this night revealed a ~50 km clear (non-luminous) break in its otherwise contiguous filamentary structure to the south of the island, with the southern tip having drifted southwest. This break was due, ostensibly, to the physical disruption of the north-to-south flow by Christmas Island, in which the filament acted as a tracer for the surface waters. The distance and orientation of the break south of the island could be explained by the speed and direction of analyzed currents when extrapolated over the 24-hr period between the two DNB images.


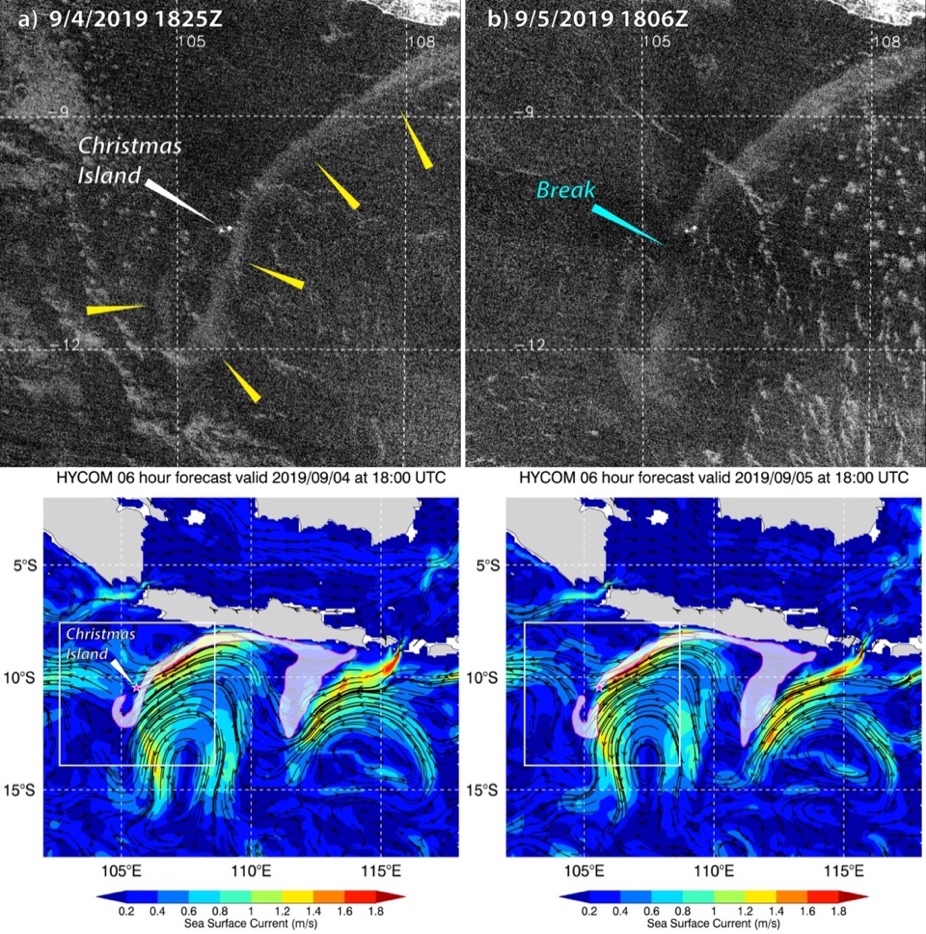


*Figure S2.9.13. The western luminous filament of the 2019 Java Phase 2 Sea intercepted Christmas Island (identifiable in DNB imagery by two artificial light areas on either side of the island) between the nights of 4 and 5 September as the filament drifted westward. The second night (b) shows a physical break in the filament to the south of the island. The flow of the water within the filament, based on HYCOM analysis (shown below each DNB image and matched within 30 min of the observation), was from northeast to southwest, with the luminous filament acting as a tracer to the barrier-disrupted flow.*

By 7 September 2019, moonlight had once again intruded upon the scene, rendering the DNB incapable of monitoring the event further on 8 September and beyond. The DNB did not detect any luminous structures in the subsequent moon-free period. Considering the significance and continuity of features observed during both Phases 1 and 2, it is likely that they in fact comprised a single extraordinary event—lasting more than 6 weeks.

# 10. Banda Sea , 26 July - 4 August 2019

This case initiated during the same time period as the major 2019 Java event described above and in the main paper. The concurrency of these two events, despite them being separated by a distance of ~2000 km, may point to a macroscale connection of environmental conditions associated with the IOD, as postulated in the main paper. The DMI in July 2019 was 0.693, and increased to 1.12 by October 2019. No events were observed concurrently in the milky sea high-frequency of occurrence areas on the western side of the Indian Ocean basin (Arabian or Somali Seas) during this same period.


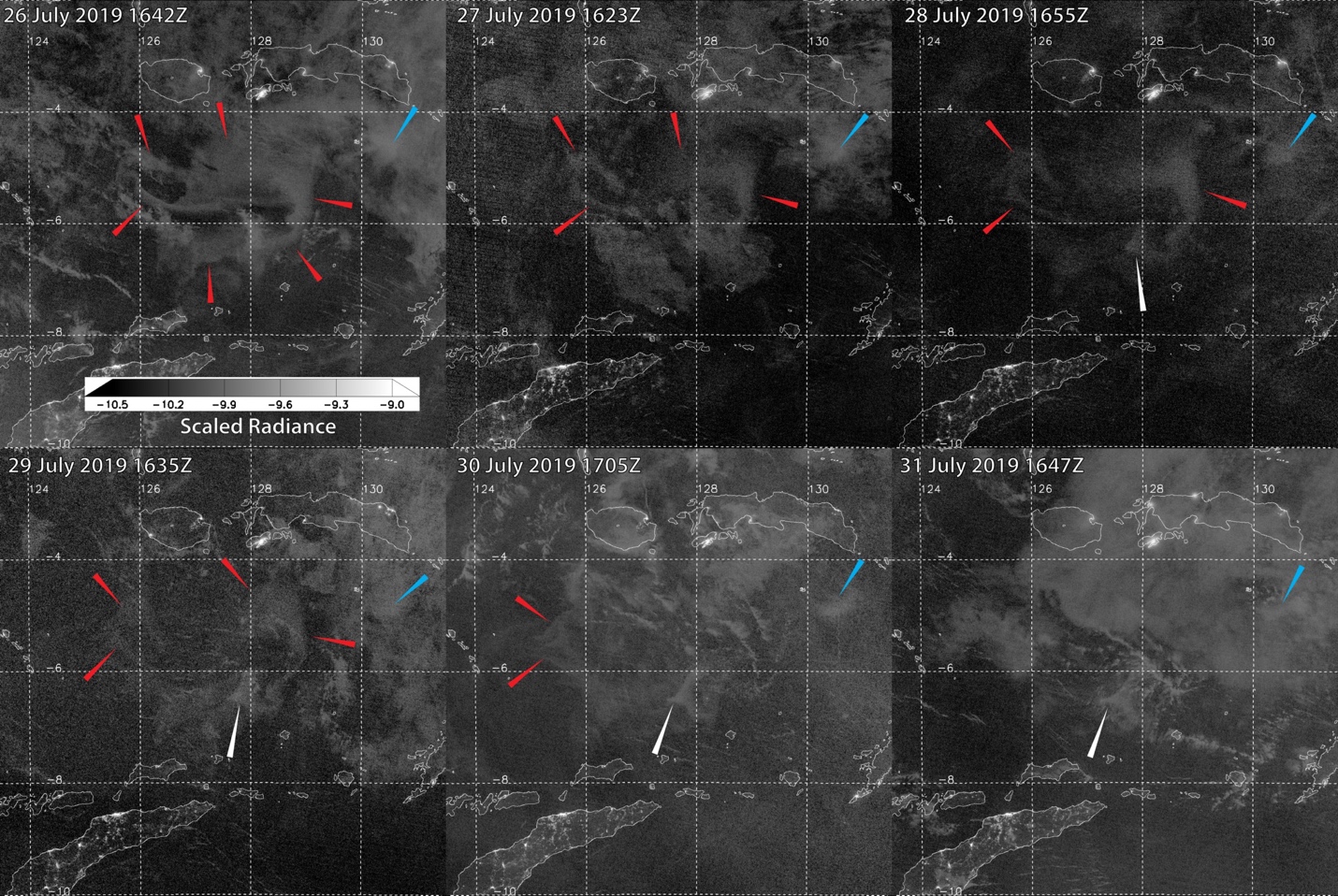


*Figure S2.10.1. Day/Night Band imagery for 26-31 July over the Banda Sea, showing several luminous features that persisted and evolved slowly over multiple successive nights amidst ephemeral cloud cover. Red, White, and Blue markers point to the heart-, arrowpoint-, and oval-shaped features (respectively) mentioned in text.*

On 27 July at ~1713 UTC, the Suomi NPP DNB captured a complex-structured luminous body in the Banda Sea (Figure S2.10.1). Notable among the persistent luminous features was a dual-arched structure that formed a ‘*heart*’ shape centered at ~(5.5° S, 127.0° E) covering ~60,000 km^2^. A second, smaller 3000 km^2^ ‘*oval*’ shaped body resided near (4.9° S, 130.9° E), about 75 km southeast of the Banda Islands. Looped DNB imagery over the period 27-29 July showed a weak clockwise rotation of the western side of the *heart*, and counterclockwise rotation of its eastern side. The *oval* remained stationary through 30 July. Despite being mostly low-cloud-covered, this feature was perceptible by the DNB via diffusion of its light through the cloud decks, as seen in other cases documented here. Beginning on 28 July, within the southeast portion of the *heart* near (6.5° S, 127.7° E), an ‘*arrowpoint*’ shape emerged, following a westward course which continued through 3 August. On 4 August and thereafter, all luminous bodies were lost due to widespread, optically thick cloud cover.


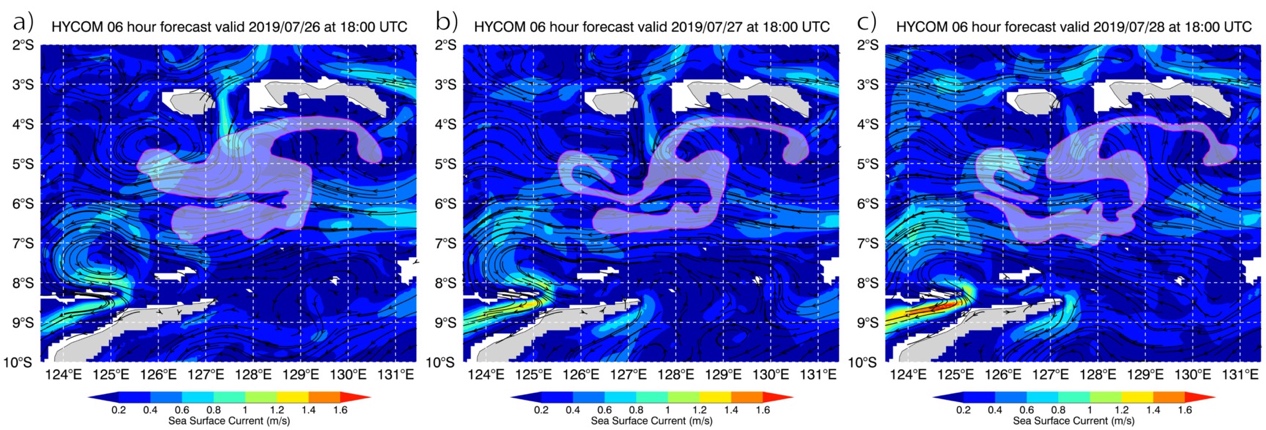


*Figure S2.10.2. HYCOM current analysis during the July-August 2019 Banda Sea case study, with approximate location of luminous body (derived from DNB imagery) overlaid as semi-transparent shape for the consecutive nights of 26-28 August 2019 at 1800 UTC.*

HYCOM currents (Figure S2.10.2) within the Banda Sea at the time were generally weak (< 0.3 m/s), with an east-to-west flow. A narrow stream of relatively stronger currents (~0.5 m/s) entered the Banda Sea from the southeast between the Indonesian islands of Yamdena and Trangan. This current meandered north by northwest toward Buru, Maluku, and exited the Banda Sea northward toward the Molucca Sea. A small counter-clockwise swirl, centered at (5° S, 127.5° E), aligned roughly with the center of the *heart* feature. The *arrowpoint* was located near the southwestward bow of the meandering pass-through current. The smaller *oval*, which remained stationary throughout the period, resided in doldrums (< 0.2 m/s, in weak clockwise circulation) east of Banda Island. Thus, the surface currents field could help to explain the shape and motion of the luminous bodies.


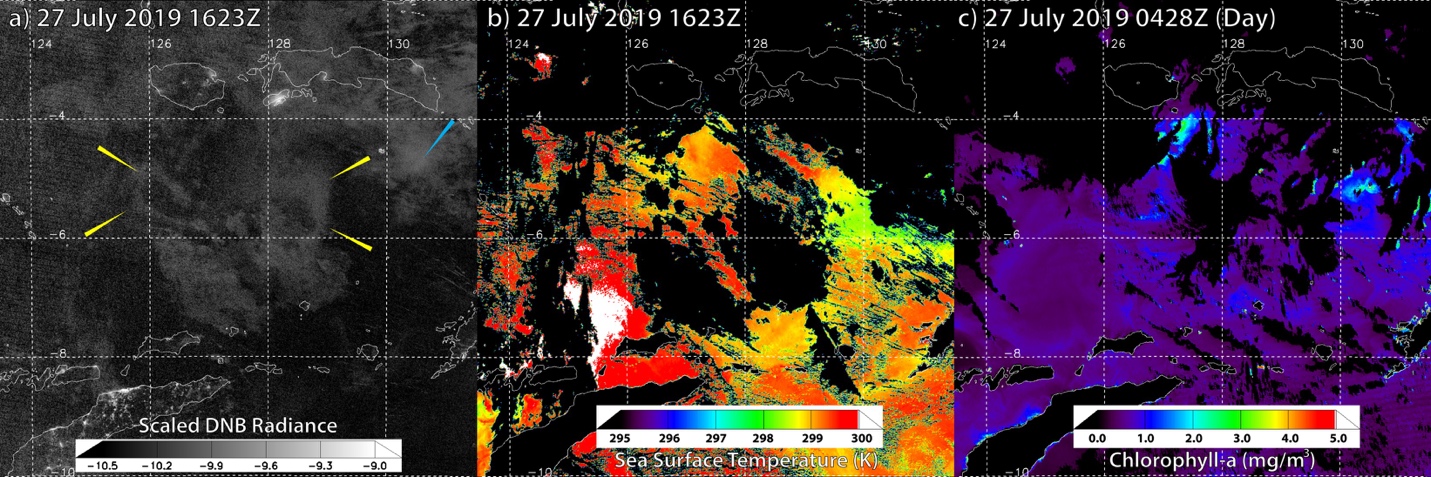


*Figure S2.10.3. Comparison of DNB scaled radiances (a) with matching retrievals of SST (b) and Chla (c; daytime) for the 2019 Banda Sea event on 27 July. Luminous bodies in DNB imagery denoted by yellow and blue pointers.*

Figure S2.10.3 compares the DNB-observed luminous body and retrievals of SST and Chla on 27 July 2019. While the region experienced heavy cloud cover over the period, the night of 27 July was relatively clear. While no significant relationships between the luminous body and SST/Chla fields appeared, an inverse correlation existed between the western-lobe of the *heart* feature and Chla, with the brightest parts of the luminous body occurring within Chla of ~0.5 mg m^-3^ which was embedded within a background of Chla in the 0.75 – 1.0 mg m^-3^ range. This analysis took into account the Chla feature’s westward drift of 22 km due to ~0.5 m s^-1^ surface currents over the 12 hr period between the nighttime DNB observation and the daytime Chla retrieval.

# 11. Socotra and Somali Sea , 7-22 January 2021

On 7 January, the first observable night of the lunar cycle, the DNB detected a “*U*”-shaped luminous body at (11.5° N, 58° E). This body drifted very slowly southwest at ~0.18 m/s over the next several nights, centred at (11.25° N, 57.5° E) by 11 January. On the next two nights it was mostly cloud-obscured, with some indications of light diffusion through the clouds, but on 14 January the skies cleared and for the next four nights the body was again well-observed. Three nights of this prime-viewing period are shown in Figure S2.11.1, during which time the isolated luminous body underwent a slow clockwise rotation. On the night of 17 January, the luminous body’s area was approximately 10,000 km^2^. The nights of 18-19 January were again cloud obscured with evidence of the feature through light diffusion, and on the 20-22 January it could again be observed amidst broken clouds, continuing in its slow west/southwestern drift.


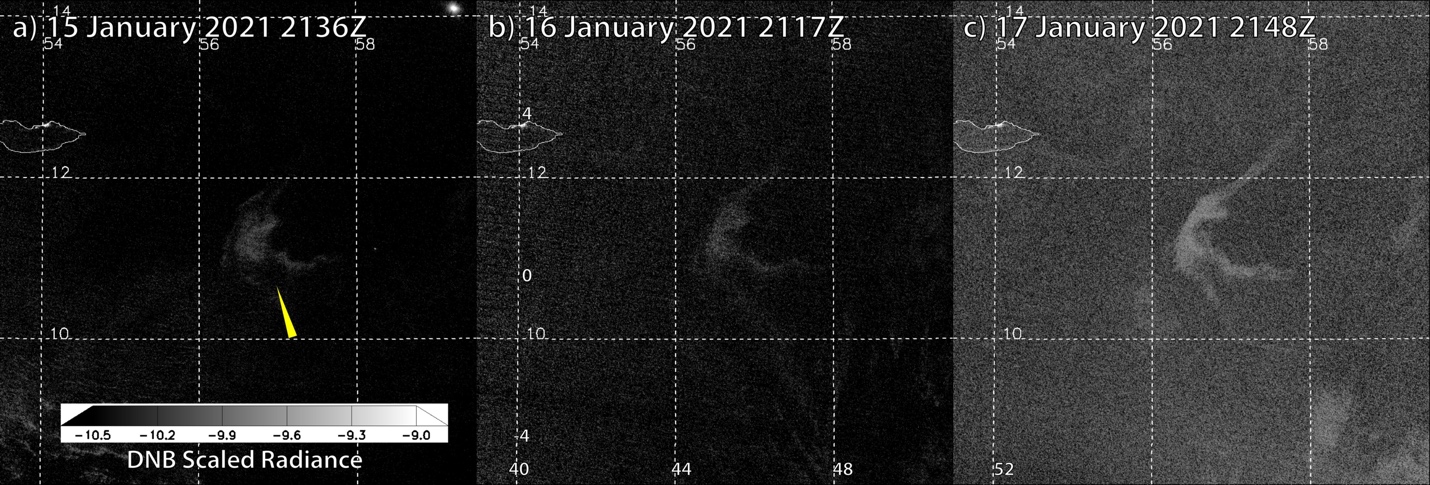


*Figure S2.11.1. A 3-consecutive-night (15-17 January 2021) sequence of Day/Night Band imagery for the September 2021 Socotra milky sea, showing a persistent luminous body ~250 km southeast of Socotra which did not correspond to the meteorological cloud field.*

HYCOM surface currents could explain the observed motion and rotation of the Socotra luminous body. Figure S2.11.2 shows the location of the body on two consecutive nights, placed atop corresponding HYCOM fields of current. The main body was situated in a weak counter-clockwise rotating doldrums situated between a clockwise eddy to its southwest and counter-clockwise eddy to its northeast. The adjacent eddies each shed some of the luminous material away from the core body, forming well-defined filaments on 17 January. The general drift and current speeds were consistent with the DNB-observed motion.


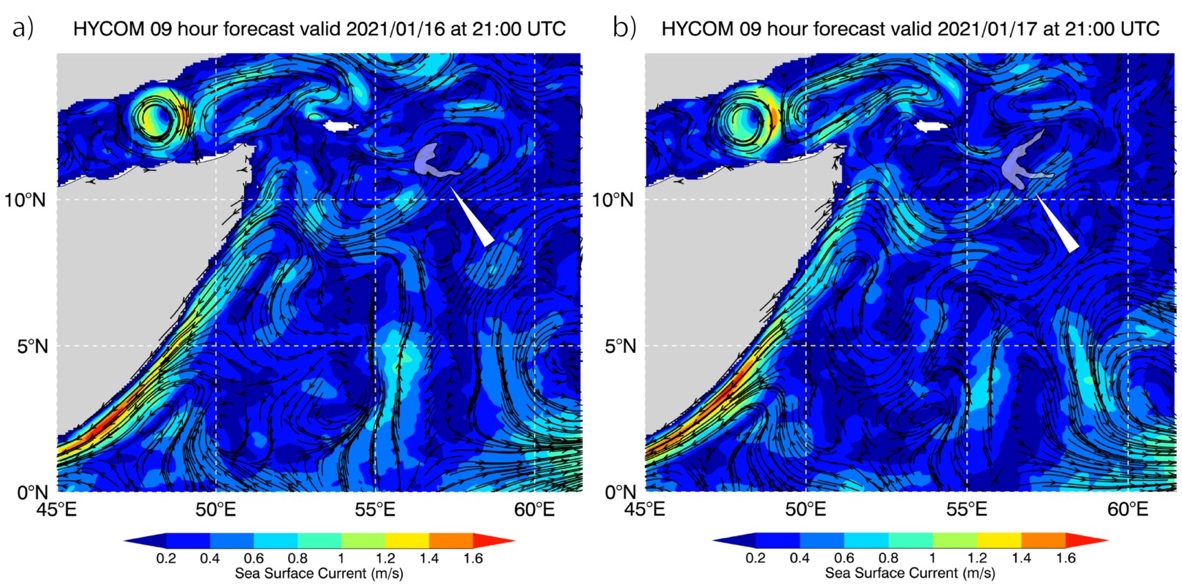


*Figure S2.11.2. HYCOM current analysis during the January 2021 Socotra case study, with approximate location of luminous body (derived from DNB imagery) overlaid as semi-transparent shape for the nights of 16-17 January at 2100 UTC—within 1 hr of the DNB image collections.*


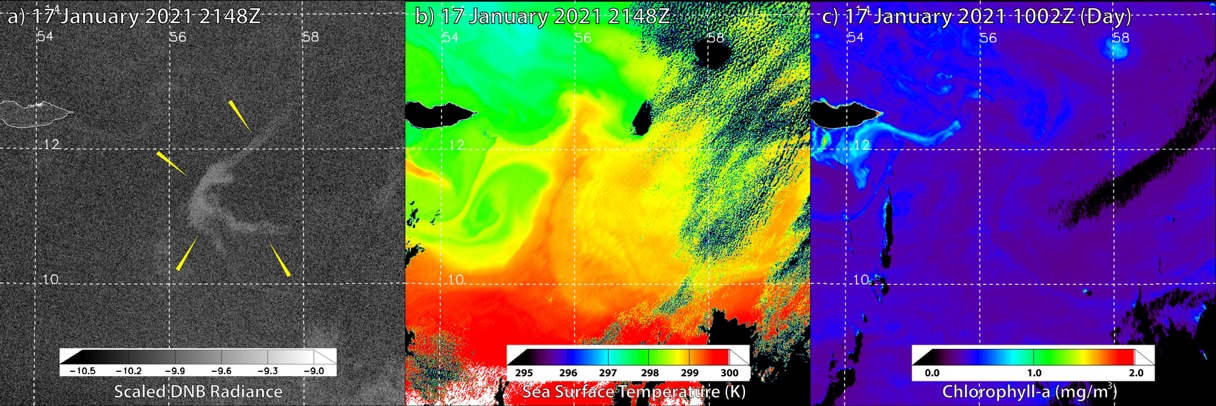


*Figure S2.11.3. Comparison of DNB log_10_-scaled radiances (a) with matching retrievals of SST (b) and Chla (c; daytime) for the 2019 Somalia event on 30 January. The luminous body in DNB imagery (yellow pointers) shares structural similarities to the SST and slightly elevated values of Chla.*

The luminous body continued in a slow westward drift for multiple ensuing nights beneath varying amounts of cloud cover. The night of 17 January DNB imagery offered a clear and near-nadir view of the event, centred at (11.2° N, 56.5° E). Figure S2.11.3 compares the DNB, SST, and Chla. The luminous body was embedded within a moderately cool oceanic front, with the brightest waters corresponding to SST values of 298.5-299 K. The daytime Chla analysis showed slightly elevated values around 0.5 mg/m^3^ that matched the body’s structure. However, elevated Chla structures not associated with luminous waters were prevalent throughout the region. A luminous filament occurred just offshore south-eastern Socotra, matching the stronger Chla structure seen in this same area. This filament was significantly less bright in DNB imagery than the main structure roughly 250 km offshore and to the southeast.


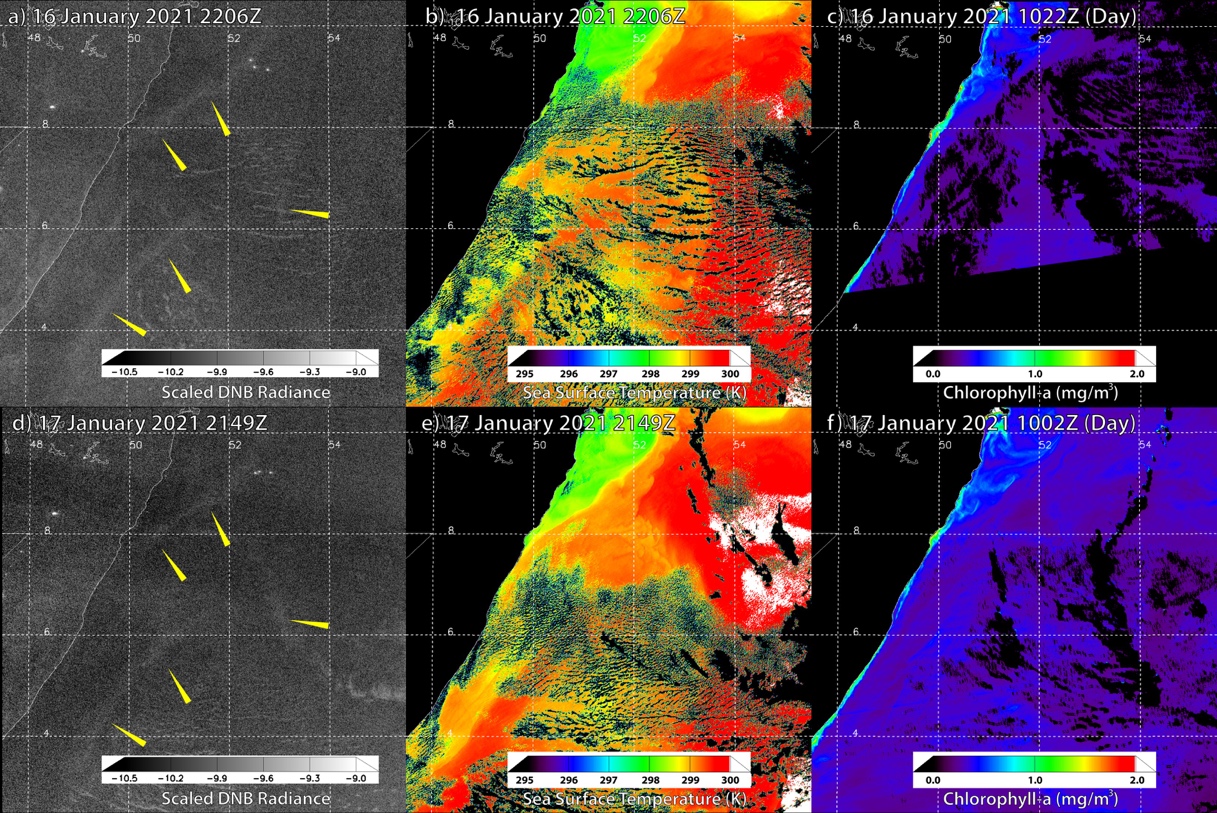


*Figure S2.11.4. Comparison of DNB scaled radiances (a,d) with matching retrievals of SST (b,e) and Chla (c,f; daytime) for the 2019 Somalia event on 16 January (top row) and 17 January (bottom row). The luminous body in DNB imagery (yellow pointers) corresponds to weak boundaries in SST and Chla.*

Concurrent to the Socotra-area event was additional milky sea activity near the Somali coast. On 15 January 2019, two luminous filaments appeared along the boundary of cooler upwelling coastal waters and warmer waters offshore. On 16 January a third filament was detected further offshore. Figure S2.11.4 shows these three-filaments were contained within a ± 4° box centred on (7° N, 52° E). These features all occurred in surface doldrums, with the two bodies closer to the coast being adjacent to stronger sea surface currents along the coastline. Compared to the concurrent Socotra luminous body, these filaments offshore of Somalia were relatively faint and had less well-defined boundaries. Over the period 15-18 January, the three filaments moved in various directions: the northern coastal body drifted southeast, the southern coastal body drifted northwest toward the coast, and the offshore body moved southwest.

# 12. Socotra, 7 - 20 February 2021

At the nearing of this current study’s completion, yet another round of winter-Monsoon mode activity in the Somali Sea was ongoing. The initial feature was spotted on 5 February—a weak-brightness filament in the Somali Sea near (0.5° N, 50.5° E). It drifted north/northwest over the course of 3 nights before fading from view on 8 February. A second luminous body was first detected on 7 February, appearing as a ~6000 km^2^ luminous patch near (7.5° N, 56.3° E), about 500 km southeast of Socotra. We focus here on the latter, more prominent event.

*
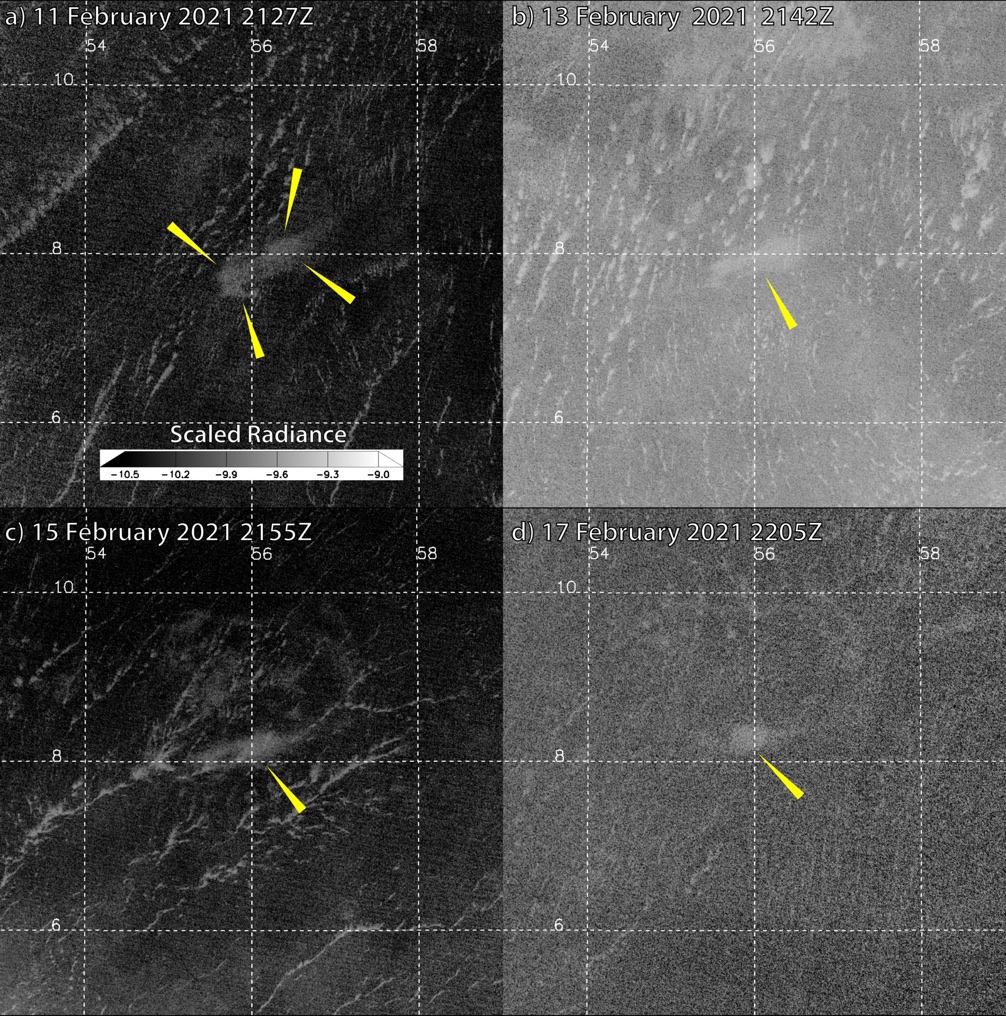
*

*Figure S2.12.1. Selected nights of Day/Night Band log_10_-scaled radiance imagery for the February 2021 Socotra milky sea, showing a luminous body near (8° N, 56° E) that persisted and drifted slowly to the northwest over an observable period of 2 weeks. Anomalous brightness in (b, d) is due to increased airglow on these nights.*

Figure S2.12.1 shows selected nights of the luminous body southeast of Socotra. Appearing prominently above the background in DNB radiance imagery, and maintaining well-defined boundaries, this body drifted slowly (~0.1 m/s) north/northwest to (8.1° N, 56.1° E) by 15 February. It continued on this track for multiple nights, keeping its oblate shape but contracting to ~2000 km by 19 February, before being in DNB imagery lost to moonlight contamination on 20 February. The body’s slow drift could be explained by HYCOM surface currents (Figure S2.12.2), showing that it was situated in a weak current zone flanked on all sides by eddies.


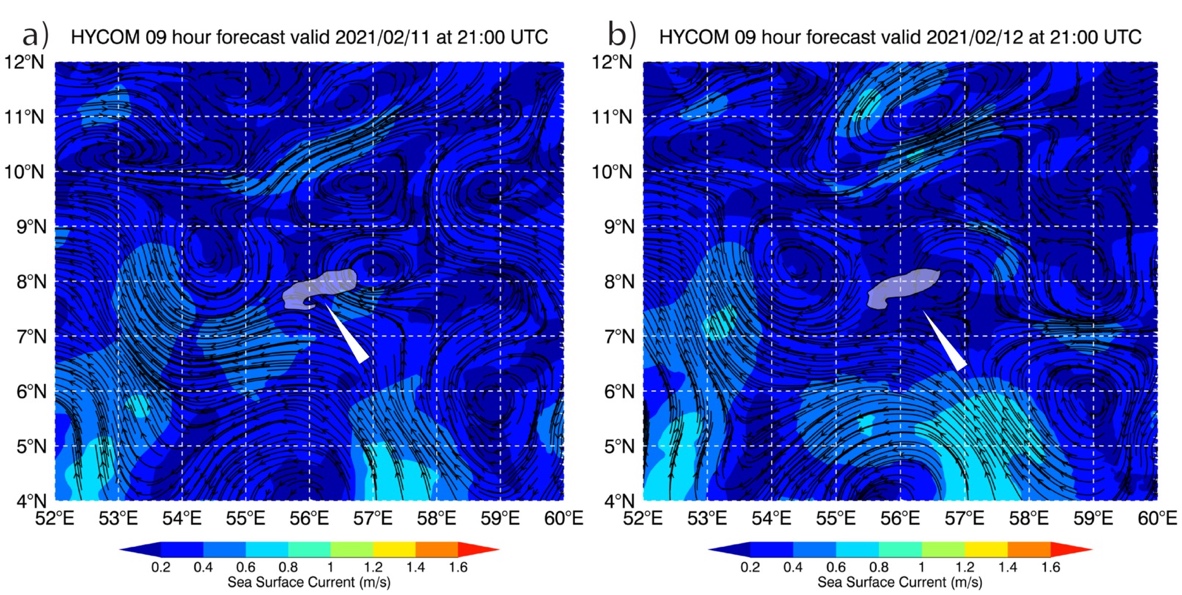


*Figure S2.12.2. HYCOM current analysis on 11-12 February 2021, 2100Z, for the Socotra case study, with approximate location of luminous body (derived from DNB imagery) overlaid for these two nights.*

*
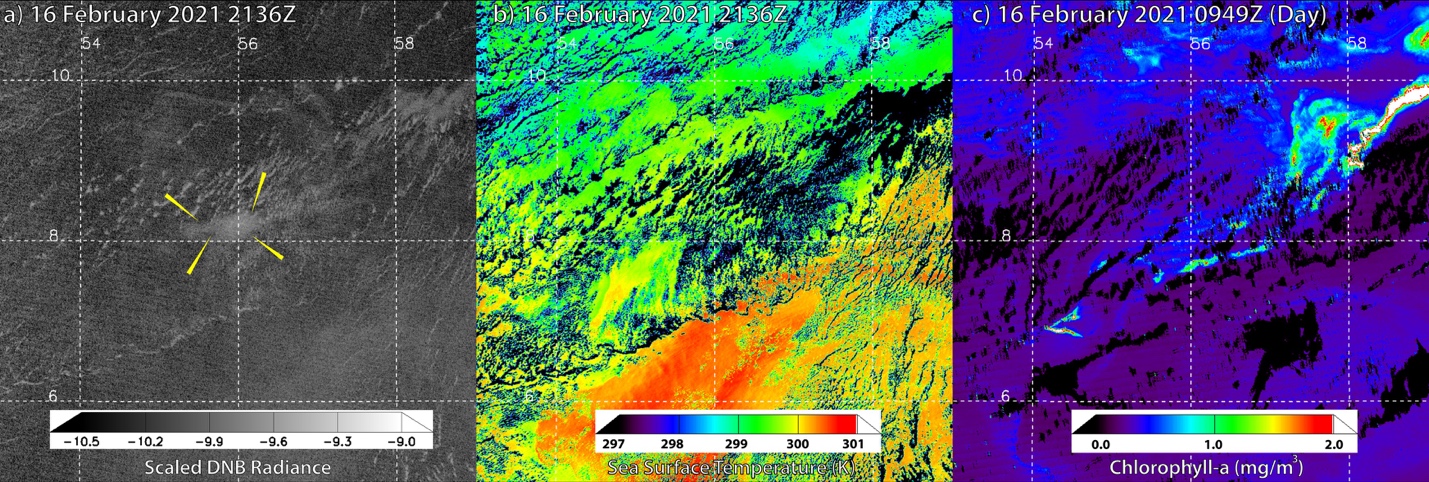
*

*Figure S2.12.3. Comparison of DNB scaled radiances (a) with matching retrievals of SST (b) and Chla (c; daytime) for the 2021 Socotra event on 16 February. The luminous body in DNB imagery is denoted by the yellow pointers.*

Analyses of ocean surface properties revealed that the luminous body resided on the cool-side of an oceanic front (Figure S2.12.3), within SST of 299 K and Chla values of ~0.5 mg / m^3^. A narrow filament of elevated Chla values, exceeding 1.0 mg / m^3^, existed along this oceanic frontal boundary, displaced roughly 50 km to the south of the luminous patch. Otherwise, there were no obvious spatial correlations between SST and Chla and the luminous body.

# 13. Summary

Over the period of December 2012 through January 2021, 12 distinct milky sea candidates were identified in the north-western Indian Ocean and Indonesian region. Among the 8 cases detected in the Somali and Arabian Seas, 2 occurred in the July-September (Summer Monsoon) period and the remaining 6 occurred in the January-February period (Winter Monsoon). Despite the dominance of summertime surface reports and the stronger upwelling conditions during the Summer Monsoon mode, the Winter Monsoon mode cases occurred with more regularity. However, it is possible that additional events did occur over this 2012-2021 period, but occurred during the ~2 week moonlight contamination periods. Several of these winter mode cases shared striking similarities in structure, timing, and locations of formation—both amongst each other and with the historical sighting record (Supplementary Discussion 1). It appears that the rhythm of the Winter Monsoon provides a robust space/time target, which when combined with satellite information bodes well for the prospects of future excursions to sample and document milky seas *in situ*.

Whereas the eastern Indian Ocean (Indonesia / Maritime Continent) proved less predictable in terms of a cadence to the observed events, all cases documented in this study occurred during the winter season (July-September in the southern hemisphere). With the exception of the weak 2014 Banda Sea event, the remaining 3 Indonesia-region events corresponded to positive phases of the IOD. As the IOD does not exhibit a regular oscillation like the Indian Monsoon on the opposite end of the basin, this may explain in part the aperiodic nature and relative infrequency of events. Additional events, combined with a retrospective analysis of existing ship reports, will be necessary to better understand the key factors governing milky seas of the Maritime Continent. We have observed by the 2019 Java Sea case that despite the lack of regular forcing, when the ingredients do come together the Maritime Continent region is capable of generating the strongest milky sea displays in the world.

Considering the limits of satellite-based remote sensing, the sense is that we have but scratched the surface of milky sea understanding in both the literal and figurative sense. Detailed water profile information within, outside of, and across the lateral boundaries of active milky seas will be necessary to learn more. While there does appear to be a consistent instantaneous relationship between luminous waters and narrow windows of SST and Chla, these parameters do not provide sufficiently unique information for isolating and targeting a specific location. However, analysis of water mass histories, and particularly flow-following of Chla fields, may contain additional clues. Until we learn more, the hunt for milky seas will thus continue to rely on DNB detections of active events with hopes of unlocking further knowledge about this intriguing and evasive wonder of nature.
