# Supplementary material for: Honing in on bioluminescent milky seas from space
Source: Sci Rep. 2021 Jul 29;11:15443. doi: 10.1038/s41598-021-94823-z (PMC8322353; doi:10.1038/s41598-021-94823-z)
Supplement: Supplementary file 3 — Supplementary Information 3. [file 41598_2021_94823_MOESM3_ESM.docx]

Supplementary Figures

*
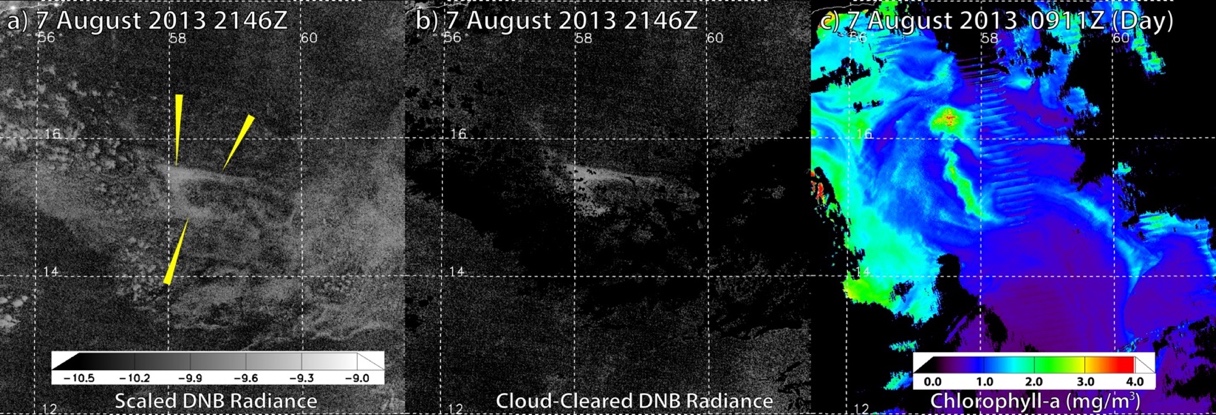
*

**Supplementary Figure 1.**  2013 Somali Sea milky sea on 7 August. **a)** DNB scaled radiances (with luminous body denoted by yellow pointers. **b)** same as **(a)** but with infrared-identified cloud cover masked to black. **c)** VIIRS-retrieved Chla from ~12 hr earlier in that day, showing a similarly curved structure close to the luminous body.


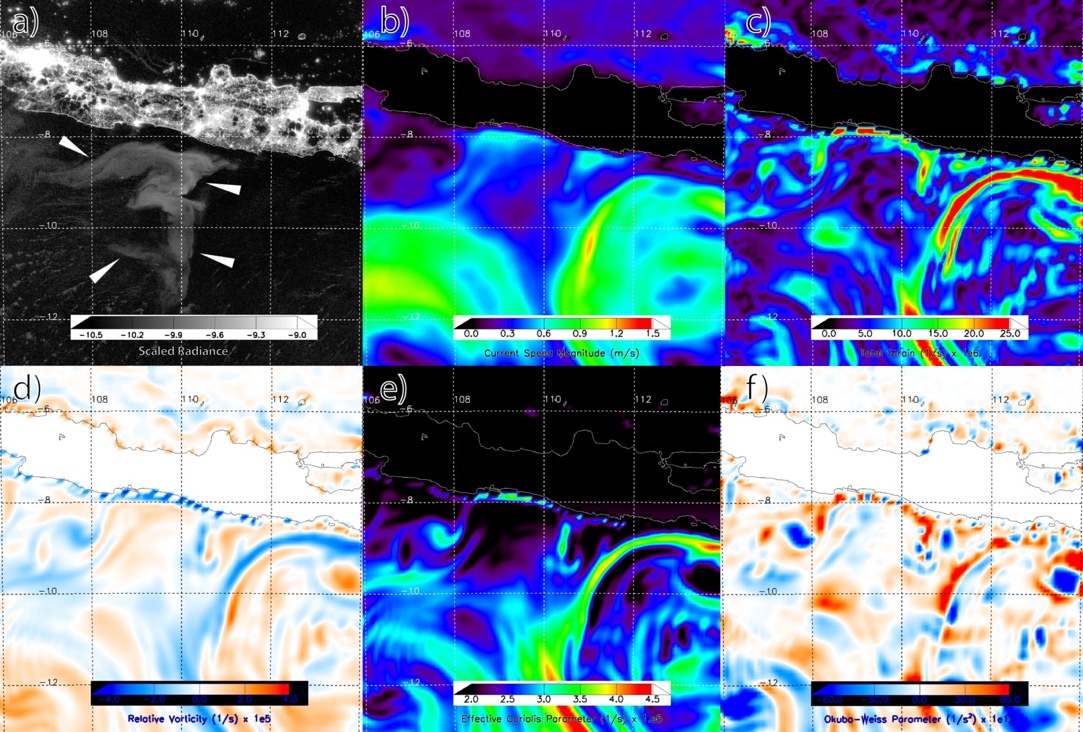


**Supplementary Figure 2.** VIIRS Day/Night Band imagery from 2 August 2019, 1753Z (**a**; white pointers denote the location of the luminous body) for the 2019 Java milky sea, along with HYCOM analysed surface current speed **(b)**, total strain **(c)**, relative vorticity **(d)**, effective Coriolis frequency **(e)**, and Okubo-Weiss parameter **(f)**. Additional details provided in *Supplementary Discussion 2*.


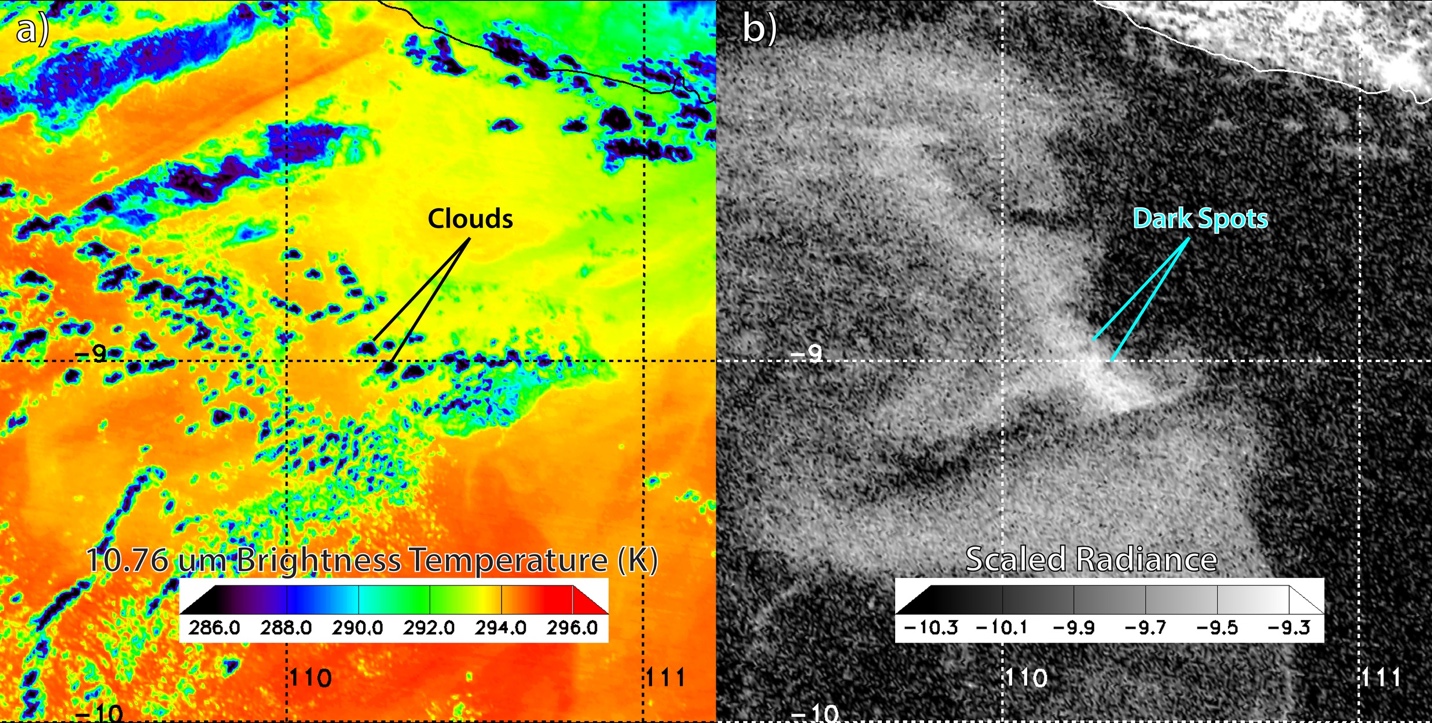


**Supplementary Figure 3.** VIIRS imagery from the 2019 Java milky sea on 30 July. Panel **(a)** shows clouds as seen in thermal infrared imagery, which correspond to relatively dark spots in Day/Night Band log_10_-scaled (W cm^-2^ sr^-1^) radiance in corresponding locations of panel **(b)**. These clouds attenuated the relatively larger upwelling radiance from below (i.e., the bioluminescent light-emitting ocean surface). Additional details provided in Supplementary Discussion 2.


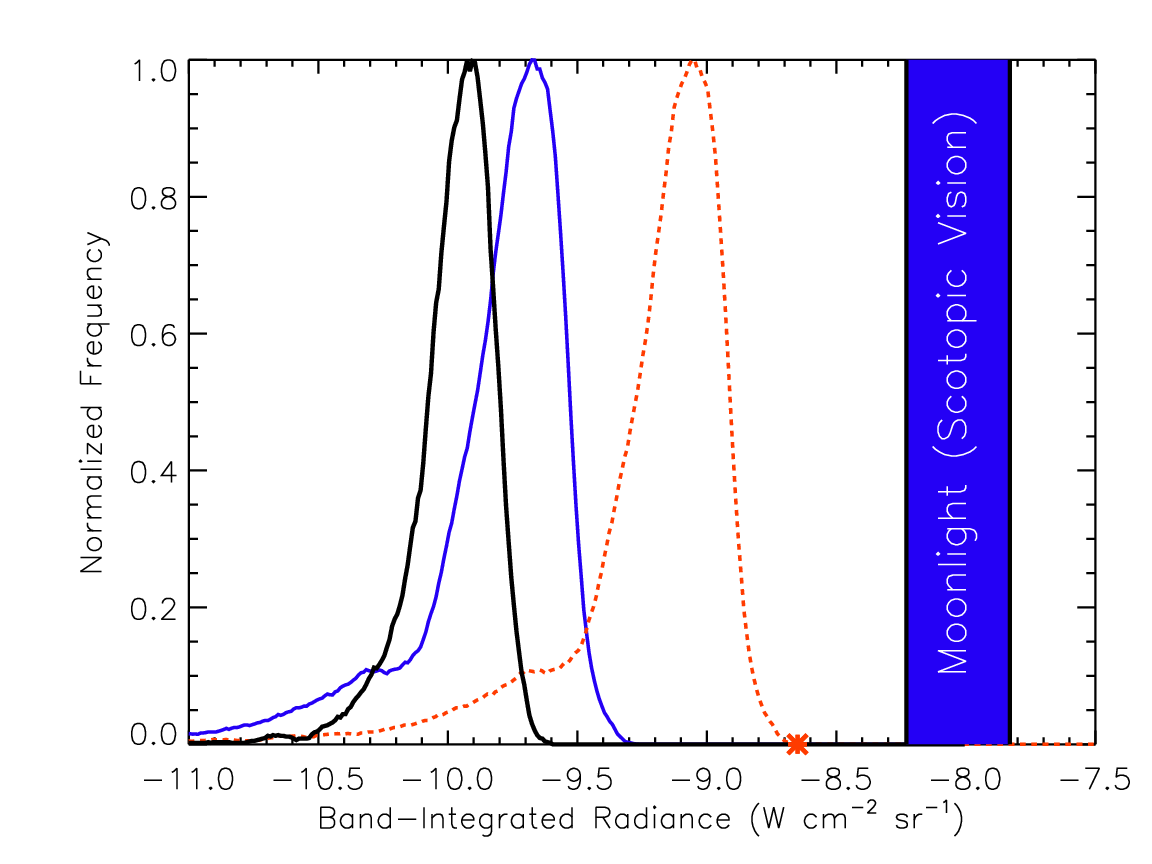


**Supplementary Figure 4.** DNB log_10_-scaled radiance distribution of clouds (reflecting airglow; black) and bright sub-region of the Java milky sea on 30 July 2019 (blue). A radiance distribution for the milky sea distribution, adjusted to scotopic-vision (4.15 multiplier, per *Methods*), is shown as a red-dashed curve. The red asterisk corresponds to the brightest scotopic-adjusted DNB log_10_-scaled radiance (-8.65 in log_10_ units, or 2.24 × 10^-9^ W cm^-2^ sr^-1^ in radiance units) of the milky sea, assuming a *V. fischeri* luminous bacteria source. A range of lunar disk radiance for human scotopic vision, assuming a 100% albedo reflective surface, is shown in the blue-filled box.


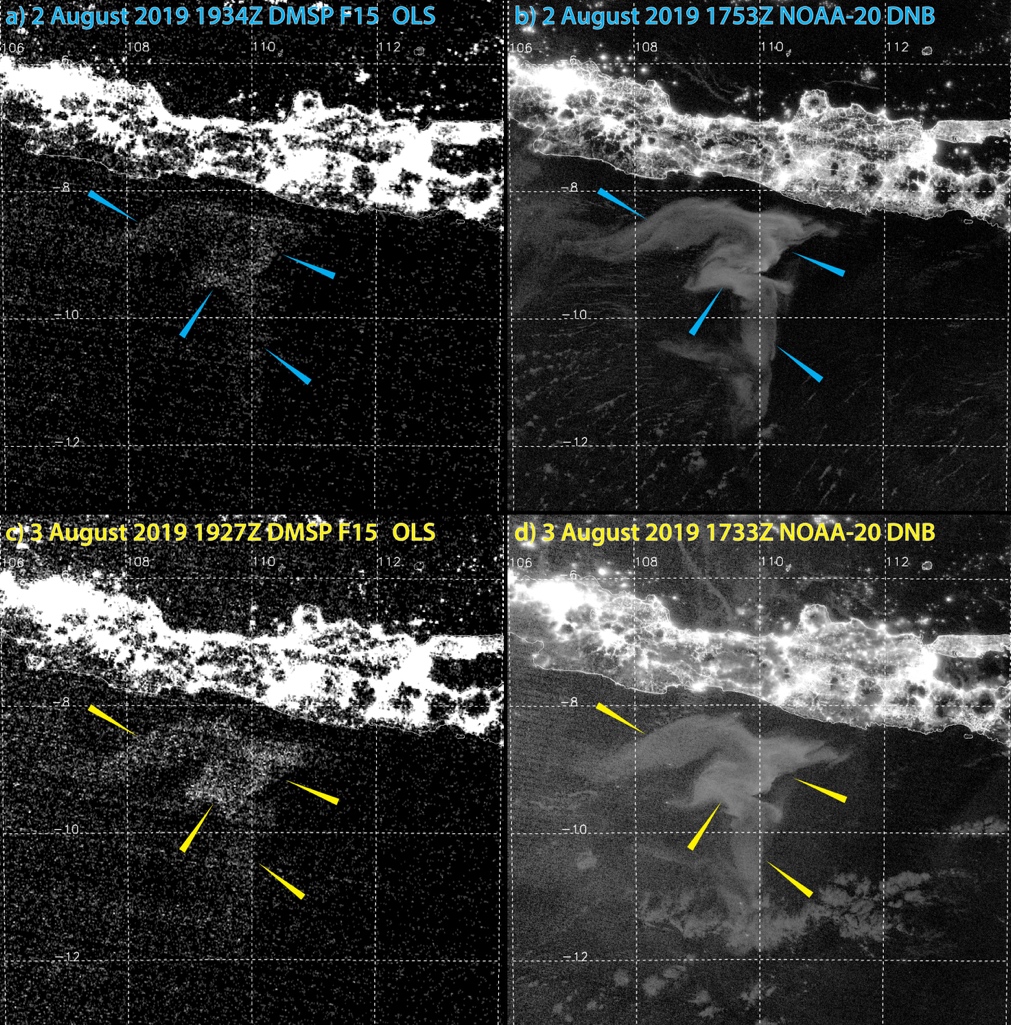


**Supplementary Figure 5.** Comparison of OLS and DNB enhanced low-light imagery of the 2019 Java milky sea, Phase 1. **(a, b)** imagery comparisons on 2 August and **(c, d)** 3 August. Pointers mark common locations in the imagery pairs. The OLS milky detection seen here is similar in brightness to that of the 1995 S.S. Lima milky sea, in which case the ocean surface reportedly appeared as a ‘snow field’ to human observers under dark-adapted conditions. The DNB provides approximately a factor of ten improved signal to noise quality, enabling improved detection. Additional details provided in Supplementary Discussion 2.


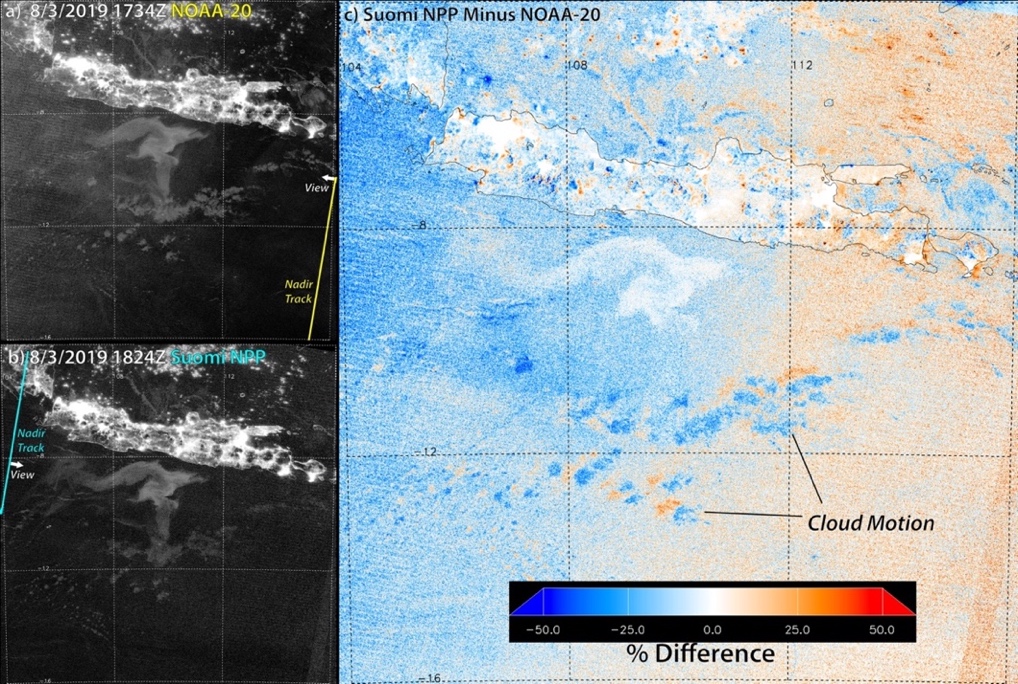


**Supplementary Figure 6**. Dual views of the Java milky sea on 3 August 2019 from **(a)** NOAA-20 (1734 Z), **(b)** Suomi NPP 50 min later (1824 Z), with satellite nadir ground tracks shown, and **(c)** the percent difference in scaled brightness between the two images. Positive/negative changes between the two images appear as red/blue, and zero change is shown in white. Whereas clouds display significant shifts due to both parallax shift and motion, the luminous body produces zero shift, including at its edges—confirming that it is a surface-based feature. Negative and positive biases on the left/right of the image are due to the van Rhijn effect of airglow. Additional details provided in Supplementary Discussion 2.


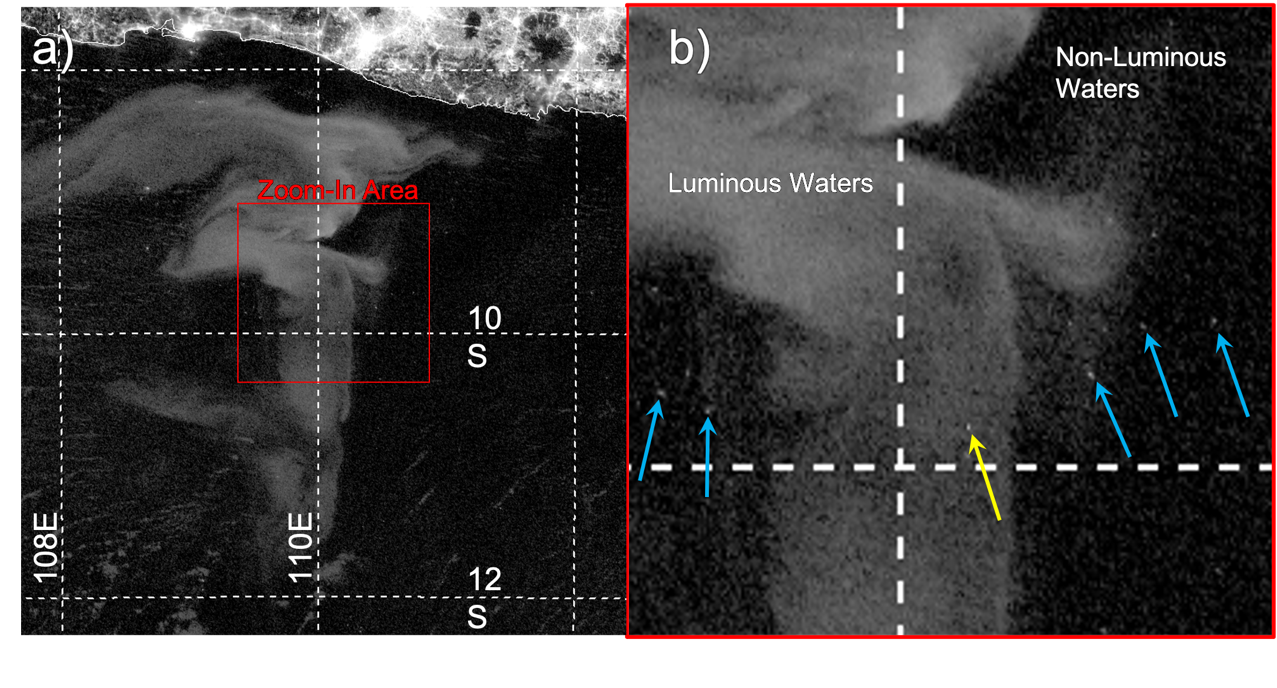


**Supplementary Figure 7.** View of the 2019 Java milky sea on 2 August 2019 1753 **UTC (a)** and inset zoomed-in area **(b)**. Arrows denote the locations of selected individual ship lights under clear skies. Turquoise arrows identify ship lights removed from the luminous waters. Yellow arrow denotes a ship light atop the luminous waters, which remains as a distinct point source. Additional details provided in Supplementary Discussion 2.
